# Immunomodulating effects of the single bacterial strain therapy *EDP1815* on innate and adaptive immune challenge responses — a randomized, placebo-controlled clinical trial

**DOI:** 10.1007/s12026-024-09484-7

**Published:** 2024-05-15

**Authors:** Boukje C. Eveleens Maarse, Micha N. Ronner, Manon A. A. Jansen, Tessa Niemeyer-van der Kolk, Aliede E. in ’t Veld, Erica S. Klaassen, Saira Ahmad, Andrea Itano, Duncan McHale, Matthijs Moerland

**Affiliations:** 1 Centre for Human Drug Research, Zernikedreef 8, 2333 CL Leiden, The Netherlands; 2 Leiden University Medical Centre, Albinusdreef 2, 2333 ZA Leiden, The Netherlands; 3 Evelo Biosciences Inc., One Kendall Square, Building 600/700, Suite 7-201, Cambridge, MA USA; 4 Veramed, 5th Floor Regal House, 70 London Road, Twickenham, TW1 3QS UK

**Keywords:** Microbiome, Autoimmune diseases, Immune challenges, Keyhole limpet haemocyanin, Imiquimod Immunodermatology

## Abstract

The gut microbiome can modulate systemic inflammation and is therefore target for immunomodulation. Immunomodulating effects of EDP1815, a bacterial commensal strain of *Prevotella histicola*, were studied in healthy participants. Effects on adaptive immunity were evaluated by a neo-antigen challenge with keyhole limpet haemocyanin (KLH), while effects on innate immunity were evaluated by topical toll-like receptor 7 (TLR7) agonist imiquimod. Capsules with two enteric coating levels (EC1, EC2) were compared. Thirty-six healthy participants were included and received a daily dose of 8 × 10^10^ cells EDP1815-EC1, EDP1815-EC2 or placebo (randomization 1:1:1) for 60 days. They received KLH vaccinations at days 8, 24 and 36, with intradermal skin challenge at day 57. KLH challenge outcomes were antibody levels, and skin blood flow and erythema after skin challenge, measured by imaging techniques. Imiquimod administration started at day 57, for 72 h. Outcomes consisted of imaging measurements similar to the KLH challenge, and the influx of inflammatory cells and cytokines in blister fluid. There was no effect of EDP1815 treatment on the KLH challenge, neither on the imaging outcomes of the imiquimod challenge. There was a consistently lower influx of inflammatory cells in the blister fluid of EDP1815-treated participants (neutrophils, *p* = 0.016; granulocytes, *p* = 0.024), more pronounced in EC1. There was a lower influx of interleukin [IL]-1β, IL-6, IL-8, IL-10, interferon [IFN]-γ and tumour necrosis factor in blister fluid of EDP1815-treated participants. EDP1815 had immunomodulatory effects on the innate immune response driven by imiquimod, but no effect on the KLH challenge was observed. Trial registration number: NCT05682222; date: 22 July 2022.

**Supplementary Information:**

The online version contains supplementary material available at 10.1007/s12026-024-09484-7

## Introduction

The gut exerts important functions in the prevention of pathogen invasion of the body: not only as physical barrier, but also as immunological barrier. The immunological barrier of the gut is formed by lymphoid tissue, known as gut-associated lymphoid tissue (GALT), that interacts with antigens of bacteria in the gut lumen [1]. Interactions between bacteria of the gut microbiome and the gut immune system can modulate systemic immune reactions [2]. Dysbiosis of the gut has been associated with inflammatory diseases [3], and there is increasing evidence that systemic inflammation may be related to gut microbiome composition [4–7], making the gut microbiome a potential target for systemic immunomodulation. One way of modulating the gut microbiome composition, thereby potentially inducing systemic immunomodulatory effects, is by probiotic strain supplementation: supplementation of live bacteria of a specific bacterial strain with beneficial properties [8]. Recently, skin diseases such as atopic dermatitis and psoriasis have been shown to be affected by probiotics treatment [9, 10].

EDP1815 is a pharmaceutical preparation of a single strain of the commensal bacterium *Prevotella histicola* (*P. histicola*), originating from the duodenum of a patient with celiac disease in remission, and has been shown to have immunomodulatory properties in preclinical and clinical studies. Contrary to probiotics, EDP1815 contains lyophilized bacteria, that are non-living and exert their function via direct interaction with immune cells. In *in vitro* studies, EDP1815 stimulated secretion of anti-inflammatory cytokines such as interleukin (IL)-10 and IL-27, with minimal induction of pro-inflammatory cytokines (data on file at Evelo Biosciences Inc.). *In vivo*, in different murine disease models, including experimental acute encephalomyelitis (EAE) and collagen-induced arthritis (CIA), treatment with *P. histicola* induced downregulation of inflammatory cytokines and induced regulatory T-cells [11–15]. In a mouse model of delayed-type hypersensitivity (DTH), consisting of immunization and subsequent intradermal skin challenge with the neo-antigen keyhole limpet haemocyanin (KLH), EDP1815 suppressed inflammation [16]. In another mouse model of skin inflammation, induced by the topical toll-like receptor (TLR) 7 agonist imiquimod, again inflammation was reduced in EDP1815-treated mice compared to placebo-treated mice [16]. In clinical studies in patients with psoriasis and atopic dermatitis, EDP1815 treatment was safe and tolerable, and gave reduction in various clinical scores disease scores [16].

Evaluating the effects of new immunomodulatory drugs on the immune system in humans is challenging as constitutively activated biomarkers are lacking. Immune challenges activating specific targets in the immune system are used to quantify the effects of new drugs on pharmacodynamic biomarkers in humans. In this study, immunomodulatory effects of EDP1815 in humans were investigated by two immune challenges, the aforementioned KLH and imiquimod models. For evaluating immunomodulatory effects on the adaptive immune system, systemic immunization and intradermal skin challenge with the neo-antigen KLH is already a widely used model [17–19]. Immunization with KLH induces the adaptive immune system, measured by antibody responses, while intradermal skin challenge induces a DTH reaction, quantified by imaging of the skin [19]. For evaluating effects on the innate immune response, the skin inflammation challenge model of topical imiquimod was used [20–22]. In this model, topical imiquimod administration results in a local psoriasis-like skin inflammation driven by nuclear factor kappa B (NFƙB) and interferon regulatory factor 7 (IRF7) pathways [23].

In a previous placebo-controlled study investigating immunomodulation by EDP1815 on the KLH challenge in healthy volunteers, EDP1815-treated participants showed a lower antibody response after KLH immunization and less inflammation of the skin after the intradermal skin challenge. However, statistical significance was not reached, possibly due to a too small sample size [16]. Therefore, the current study again investigated immunomodulation by EDP1815 in healthy volunteers, with the following adjustments: (1) capsules with a thinner enteric coating were compared to capsules with coating similar to the previous study, hypothesizing that a thinner coating would lead to earlier release and thereby more exposure of EDP1815 to gut lymphocytes, (2) the KLH regimen was changed, with 3 instead of 1 KLH vaccinations leading to stronger immune stimulation and, (3) the imiquimod challenge was added to evaluate the impact of EDP1815 on TLR7-mediated responses. It was hypothesized that EPD1815 treatment would have inhibiting effects on the innate and adaptive immune responses, with a stronger effect of the capsules with a thinner enteric coating. Taken together, this study aimed to demonstrate proof-of-mechanism for effectiveness of EDP1815 in the treatment of auto-immune skin conditions, such as eczema and psoriasis.

## Materials and methods

### Study design

This was a single-centre, randomized, double-blind, placebo-controlled trial including 38 healthy volunteers aged between 18 and 45 years, conducted at the Centre for Human Drug Research, Leiden, The Netherlands between June and October 2022. Recruitment of participants took place between 27 May and 6 July 2022. All participants gave written informed consent according to the Declaration of Helsinki recommendations prior to any study-related activity. The study was approved by the Independent Ethics Committee of the Foundation ‘Evaluation of Ethics in Biomedical Research’ (Stichting Beoordeling Ethiek Biomedisch Onderzoek), Assen, The Netherlands, was performed according to Good Clinical Practice (GCP) and was registered in the clinicaltrials.gov register under number NCT05682222. All participants underwent medical screening including medical history, physical examination, 12-lead electrocardiography and safety chemistry and haematology blood sampling. The key exclusion criteria were an active or recurrent infection, gastrointestinal tract disease, previous exposure to KLH or diagnosis with psoriasis or eczema. A complete list of the inclusion and exclusion criteria can be found in the Supplementary material.

### EDP1815

EDP1815 and placebo capsules were provided by Evelo Biosciences Inc. (Cambridge, MA, USA) and produced by Cambrex (East Rutherford, NJ, USA). Capsules contained lyophilized, thus non-living, cells of a specific bacterial strain of *P. Histicola*. The selected dose of 8 × 10^10^ cells per day was well-tolerated, safe and showed clinical efficacy in a prior phase 2 study [16]. The duration of treatment (i.e. 60 days) was based on providing sufficient inhibition of the KLH challenge by already starting treatment 7 days before the first KLH immunization. The two enteric coatings (ECs), EC1 and EC2, had different polymer coating levels, with approximately 58 mg dry-weight enteric polymer coating for EC1, and 14 mg dry-weight enteric polymer coating for EC2.

### Treatments and randomization

**Fig. 1:**
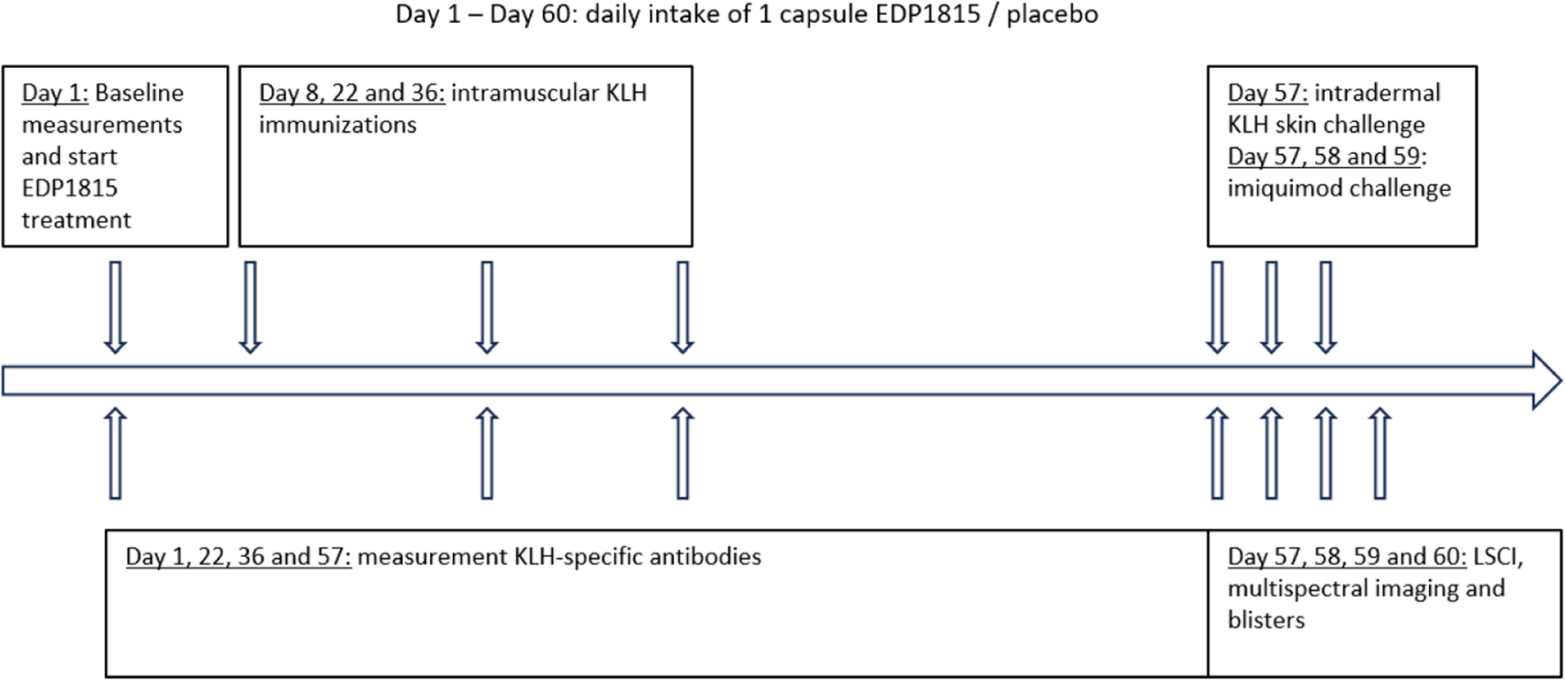
Schematic study overview. KLH, keyhole limpet haemocyanin; LSCI, laser speckle contrast imaging

Participants were studied in 2 cohorts of 18 participants. In each cohort, participants were randomized 2:1 to receive active or placebo treatment, respectively. Placebo participants of both cohorts were pooled in the analysis, resulting in a 1:1 randomization. The randomization code was generated by a study-independent statistician and was made available for data analysis after study completion and database lock. EDP1815 capsules and placebo capsules were identical in appearance and packaging, and were distributed according to the randomization numbers, ensuring concealment of treatment allocation. A schematic overview of the study timeline is shown in Fig. 1. Participants started EDP1815 or placebo treatment (Cohort 1: EC1, Cohort 2: EC2) at day 1 and took one capsule per day for 60 days. Treatment compliance of EDP1815 was measured by paper diaries that were filled in by participants and by counting the remaining capsules that participants handed in.

For the KLH challenge, participants received intramuscular KLH immunizations containing 0.1 mg Immucothel® adsorbed into Alhydrogel® containing 1.32 mg Al(OH)_3_ on days 8, 22 and 36, and received an intradermal skin challenge injection containing 0.001 mg Immucothel® in 0.1 mL NaCl in the arm at day 57. For the imiquimod challenge, participants received topical applications of 100 mg Aldara®, each containing 5 mg imiquimod, under occlusion by a 12-mm Finn chamber (Bipharma, Almere, The Netherlands) at 3 different areas on the back, starting at day 57. There was one area with 24 h of imiquimod exposure (1 application), one area with 48 h of imiquimod exposure (2 applications) and one area with 72 h of imiquimod exposure (3 applications). To ensure sufficient imiquimod delivery through the skin barrier, tape stripping of the skin was conducted before the first application of imiquimod, as described previously [20].

### Pharmacodynamic outcomes — imaging-based endpoints

Skin responses were quantified by measuring cutaneous blood perfusion and erythema at 4, 24, 48 and 72 h after the intradermal KLH challenge, and at the same timepoints (except for 4 h) during the topical imiquimod challenge. Cutaneous blood perfusion was measured by laser speckle contrast imaging (LSCI) (PeriCam PSI System, Perimed AB, Järfälla, Sweden), and erythema was measured by multispectral imaging (Antera 3D®, Miravex, Dublin, Ireland), as described earlier [17]. Cutaneous blood perfusion, i.e. basal flow, and homogeneity of cutaneous blood perfusion, i.e. flare, were expressed in arbitrary units (AUs). Erythema was measured using the CIELab a* Antera 3D® software modalities. The CIELab a* value expressed colour as a numerical value on a green-red colour scale, also measured in AUs.

### Pharmacodynamic outcomes — humoral immune response to KLH

The specific B-cell response to KLH immunization was measured by anti-KLH IgM and IgG serum titres. Serum samples were obtained by venapuncture in serum clot activator tubes (Vacutainer®) at days 1, 22, 36 and 57, centrifuged at 2000g for 10 min at a temperature of 2–8 °C and aliquoted. Aliquots were stored at a temperature of <−40 °C until shipment and analysis. Antibody levels were measured by quantitative enzyme-linked immunosorbent assay (ELISA) by Ardena Bioanalytical Laboratory (Assen, The Netherlands) and were expressed as relative ratios to the mean optical density of baseline samples.

### Pharmacodynamic outcomes — cells and cytokines in blister fluid on imiquimod-treated skin

On the imiquimod-treated skin, suction blisters were induced as described previously [24] at baseline and after respectively 24, 48 and 72 h of imiquimod application. Blister fluid (including blister cells) was collected in a V-bottom plate containing 50 μL 3% sodium citrate (Sigma–Aldrich, St. Louis, MO, USA) in phosphate-buffered saline (PBS; Gibco) and kept on ice. Within 1 h after fluid collection, the fluid was centrifuged to separate the supernatant from the cells in the blister. After centrifuging, the supernatant was collected, and the remaining cell pellet was resuspended in FACS buffer. The cells were stained for flow cytometry analysis with the antibodies listed in Table S1. After staining, the cells were washed and analysed with a MACSQuant 16 analyser (Miltenyi Biotec). The gating strategy of the blister cells is shown in Fig. S1. All flow cytometry data was analysed using absolute cell numbers acquired after staining the cells.

The collected supernatant was weighed to calculate the total amount of fluid per blister and frozen at −80 °C. Cytokine concentrations in the supernatant (tumour necrosis factor [TNF], interferon [IFN]-α, IFN-γ, interleukin [IL]-1β, IL-4, IL-6, IL-8, IL-10, IL-13, IL-33 and CXC-motif chemokine ligand 10 [CXCL-10]) were quantified by an immunoassay using Meso Scale Discovery Vplex-2 method (Rockville, MD, USA) by Ardena Bioanalytical Laboratory (Assen, The Netherlands).

### Outcome measures

The primary outcome of the study was basal flow 24 h after the intradermal KLH skin challenge. Other pharmacodynamic outcomes of the study were the other imaging outcomes of the KLH and imiquimod challenge as described above, specific B-cell response to KLH immunizations measured by anti-KLH IgM and IgG levels, and influx of cells and cytokines in blister fluid during the imiquimod challenge. Safety and tolerability were monitored by physical examination, assessment of vital signs, laboratory parameters (i.e. full blood count, biochemistry and urine analysis) and ECG data from 12-lead ECGs at regular intervals. Participants were monitored continuously for adverse events (AEs).

### Statistics

The sample size was based on the primary endpoint, basal flow (measured by LSCI) 24 h after intradermal KLH skin challenge that showed a standard deviation of 10.3 AU in previous studies. It was calculated that a sample size of 12 in each group would result in a power of 0.80 to detect a difference in means of 12.3 AU, using a two-sample *t*-test with a 0.05 two-sided significance level. Continuous values of baseline characteristics were summarized by mean and standard deviation; qualitative baseline characteristics by counts and percentages. To detect significant treatment effects, all repeated measured pharmacodynamic (PD) parameters were analysed by a mixed model analysis of covariance (ANCOVA) with treatment, time and treatment by time as fixed factors, the participant as random factor and the (average) baseline measurement as covariate. For all outcome measures, treatment effects were determined for three contrasts: EDP1815-EC1 vs placebo, EDP1815-EC2 vs placebo and EDP1815 overall vs placebo, with each contrast having a separate p-value. Anti-KLH antibodies were analysed without baseline measurement as covariate. Cytokine concentrations in blister fluid were corrected for the volume of each blister and the dilution with 50 μL of PBS sodium citrate. The treatment effects (EDP1815-EC1 vs placebo, EDP1815-EC2 vs placebo, and EDP1815 overall vs placebo) were reported with the estimated difference and the 95% confidence interval, the least square mean estimates (LSM) and the *p*-values. For PD values below the limit of quantification, a value of half the lower limit of quantification was used. Missing data or assessments were not imputed. *p*-values < 0.05 were considered statistically significant. All statistical analyses were performed using SAS for Windows version 9.4 (SAS Institute, Inc., Cary, NC, USA). All figures were created using GraphPad Prism version 9 (GraphPad Software, Boston, MA, USA).

## Results

### Safety and tolerability

Thirty-eight participants were enrolled in the study, of which two participants withdrew during study participation (Table 1: Baseline characteristics; Fig. S2: Flowchart). Thirty-six participants completed the study and were included in the analyses. For all participants included in the analyses, treatment compliance of EDP1815 was > 80% (Table S2). EDP1815 was safe and well-tolerated. As shown in Table S3, no serious or severe adverse events were reported, and all reported adverse events (AEs) were mild. An overview of all observed AEs, also classified by possible treatment (i.e. EDP1815) relatedness, is shown in Table S4 and S5. One participant developed a skin abnormality diagnosed as erythema annulare at the inner left leg, possibly related to EDP1815 use. Treatment and study participation of this participant was discontinued because of this condition. It resolved spontaneously after 2 months. As shown in Table S6-S9, there were no abnormalities of interest in the other safety outcomes. EDP1815 treatment did not result in changes in laboratory results or have any effect on vital signs or ECG measurements.

**Table 1:** Baseline characteristics

	EDP1815-EC1 (*N* = 13)	EDP1815-EC2 (*N* = 13)	Placebo (*N* = 12)
Sex (*N* male, %)	6, 46.2%	7, 53.8%	10, 83.3%
Age, years (mean ± SD)	25.5 (5.2)	26.5 (7.1)	27.4 (5.9)
BMI, kg/m^2^ (mean ± SD)	24.32 (2.61)	22.98 (2.45)	23.75 (3.36)
Body weight, kg (mean ± SD)	75.64 (14.25)	73.18 (10.23)	78.13 (15.30)
Systolic blood pressure, mmHg (mean ± SD)	117 (9.3)	112 (9.3)	125 (11.1)
Diastolic blood pressure, mmHg (mean ± SD)	68 (6.9)	68 (6.7)	71 (7.4)
Heart rate, beats per minute (mean ± SD)	57 (6.2)	57 (7.7)	60 (8.7)
CRP, mg/L (mean ± SD)	1.69 (1.46)	1.29 (1.61)	1.35 (1.81)
AST, U/L (mean ± SD)	24 (4.7)	21 (7.9)	21 (6.7)
ALT, U/L (mean ± SD)	24 (11.0)	21 (11.1)	24 (12.6)
GGT, U/L (mean ± SD)	16 (7.6)	15 (7.5)	17 (10.9)
Fasted glucose, mmol/L (mean ± SD)	4.6 (0.23)	4.8 (0.38)	4.9 (0.37)
Sodium, mmol/L (mean ± SD)	139 (1.8)	140 (2.1)	140 (2.2)
Potassium, mmol/L (mean ± SD)	4.3 (0.23)	4.5 (0.48)	4.4 (0.37)
Triglycerides, mmol/L (mean ± SD)	0.82 (0.27)	1.09 (1.02)	1.04 (0.61)

*BMI* body mass index, *CRP* C-reactive protein, *AST* aspartate transaminase, *ALT* alanine transaminase, *GGT* gamma-glutamyl transferase, *SD* standard deviation

### KLH challenge

**Fig. 2:**
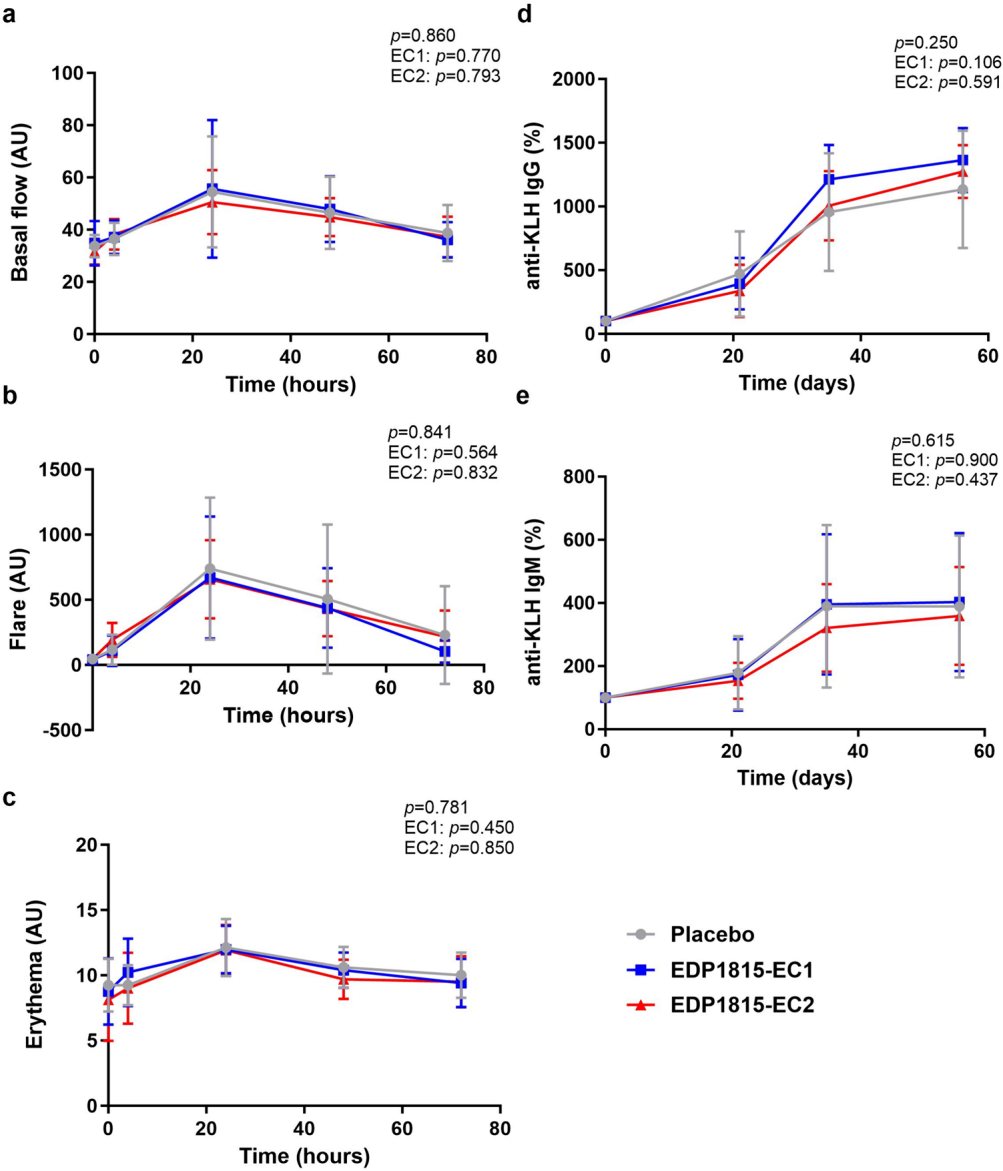
Treatment effect on keyhole limpet haemocyanin (KLH) challenge responses. Basal flow (AU) (**a**), flare (AU) (**b**) and erythema (AU) (**c**) of the skin over time after intradermal KLH skin challenge, measured by laser speckle contrast imaging (LSCI) and multispectral imaging. KLH-specific IgG antibodies (**d**) and KLH-specific IgM antibodies (**e**) over time during KLH immunizations, expressed as % compared to baseline. Participants were immunized at day 8, 22 and 36. Mean (standard deviation). AU arbitrary unit. *p*-values refer to the following contrasts: upper *p*-value EDP1815 overall vs placebo; EC1 EDP1815-EC1 vs placebo; EC2 EDP1815-EC2 vs placebo

In subjects who had received placebo, the intradermal KLH skin challenge resulted in an increase in basal flow, flare and erythema with a peak at 24 h after intradermal injection (Fig. 2). These indicators of skin blood flow normalized during the following days and returned to baseline levels after 72 h. In the placebo group, the levels of KLH antibodies increased with every KLH immunization, with the steepest increase after the second KLH immunization (Fig. 2). There was no difference between the participants who received EDP1815-EC1 or EDP1815-EC2 and participants who received a placebo in basal flow at 24 h after intradermal KLH skin challenge, neither at other timepoints (4 h, 48 h and 72 h) (EC1: *p* = 0.770; EC2, *p* = 0.793; EDP1815 overall, *p* = 0.860). There was also no treatment effect on flare at any of the timepoints after intradermal KLH skin challenge (EC1, *p* = 0.564; EC2, *p* = 0.832; overall* p = *0.841), neither on erythema (EC1, *p* = 0.450; EC2, *p = *0.850; overall* p = *0.781) (Fig. 2 and Table S10). EDP1815 treatment did not modulate the anti-KLH antibody responses over time, both for anti-KLH IgM and IgG antibodies (IgM: EC1, *p* = 0.900; EC2, *p =* 0.437; overall* p =* 0.615. IgG: EC1, *p* = 0.106; EC2, *p = *0.591; overall *p* = 0.250) (Fig. 2 and Table S10).

### Imiquimod challenge

**Fig. 3:**
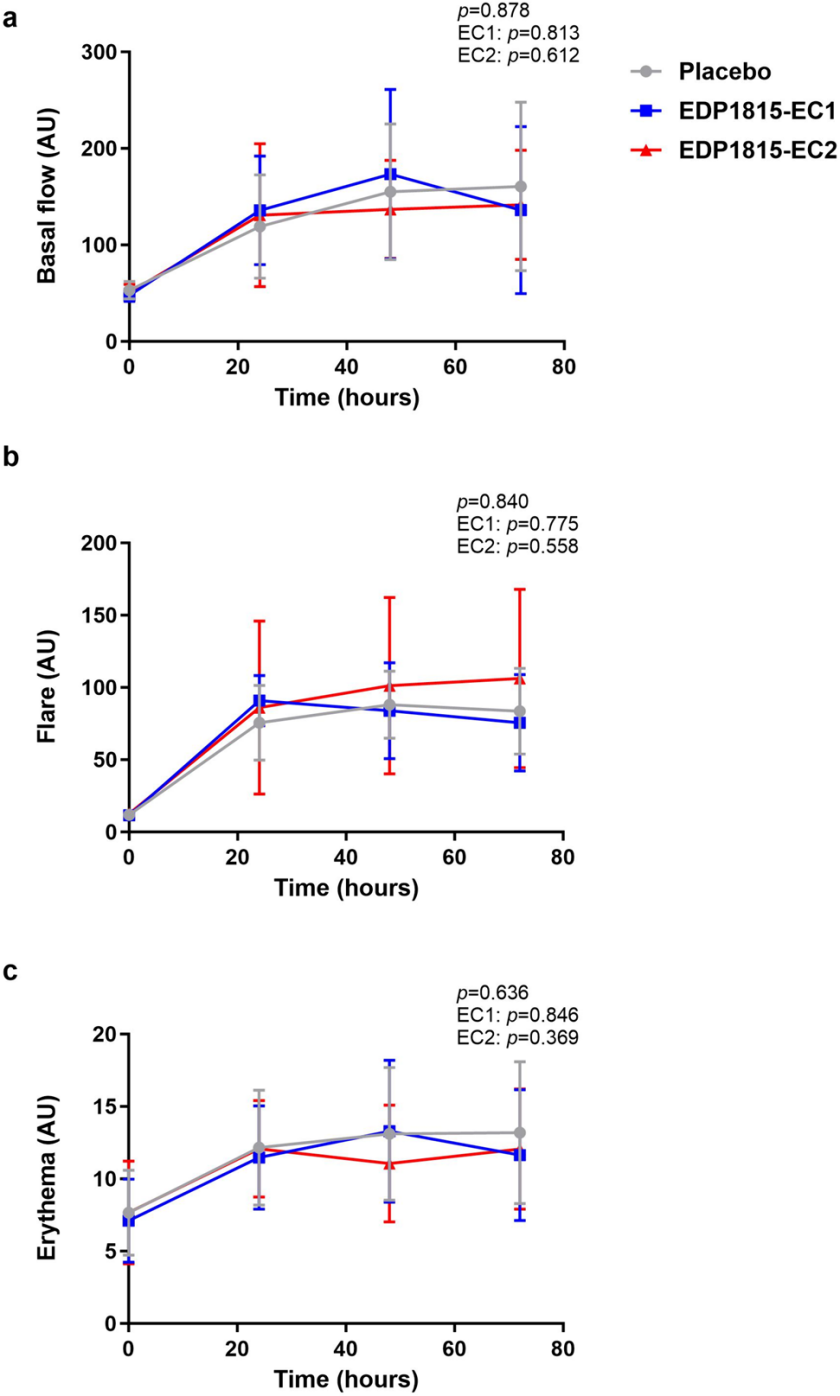
Treatment effect on imaging outcomes of imiquimod challenge. Basal flow (AU) (**a**), flare (AU) (**b**) and erythema (AU) (**c**) of imiquimod-treated skin during the imiquimod challenge, measured by laser speckle contrast imaging (LSCI) and multispectral imaging. Mean (standard deviation). AU arbitrary unit. *p*-values refer to the following contrasts: upper *p*-value EDP1815 overall vs placebo; EC1 EDP1815-EC1 vs placebo; EC2 EDP1815-EC2 vs placebo

**Fig. 4:**
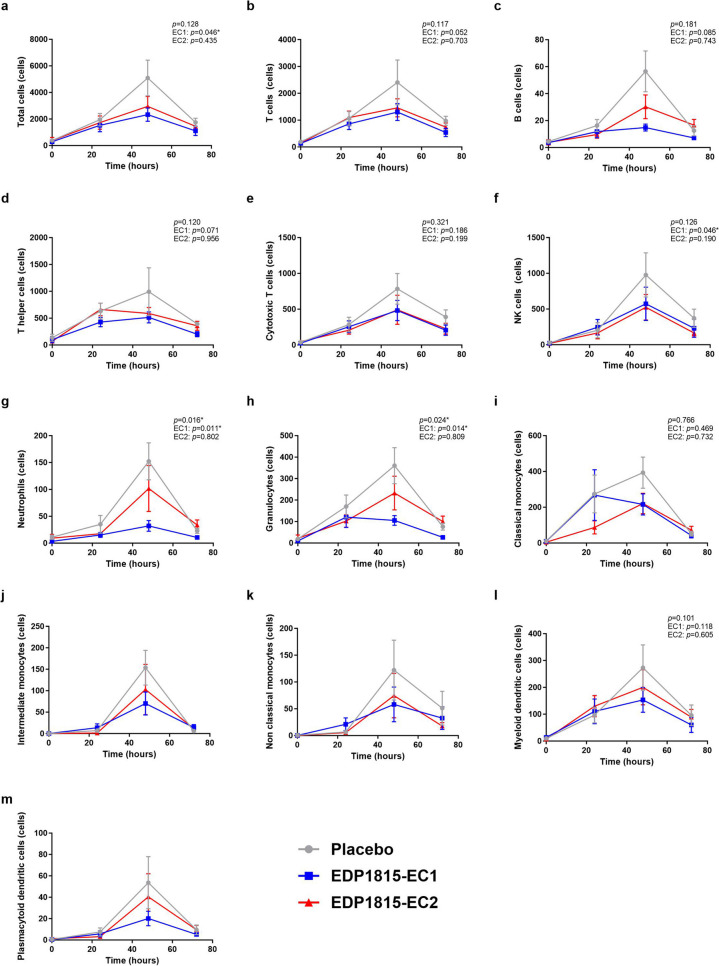
Treatment effect on influx of inflammatory cells in blister fluid during imiquimod challenge.** a**–**m **Absolute numbers of inflammatory cells in blister fluid of blisters induced on imiquimod-treated skin during the imiquimod challenge. Mean (standard deviation). *p*-values could not be calculated for intermediate monocytes, non-classical monocytes and plasmacytoid dendritic cells due to a non-normal distribution of measurements. *p*-values refer to the following contrasts: upper *p*-value EDP1815 overall vs placebo; EC1 EDP1815-EC1 vs placebo; EC2 EDP1815-EC2 vs placebo. *p < 0.05

**Fig. 5:**
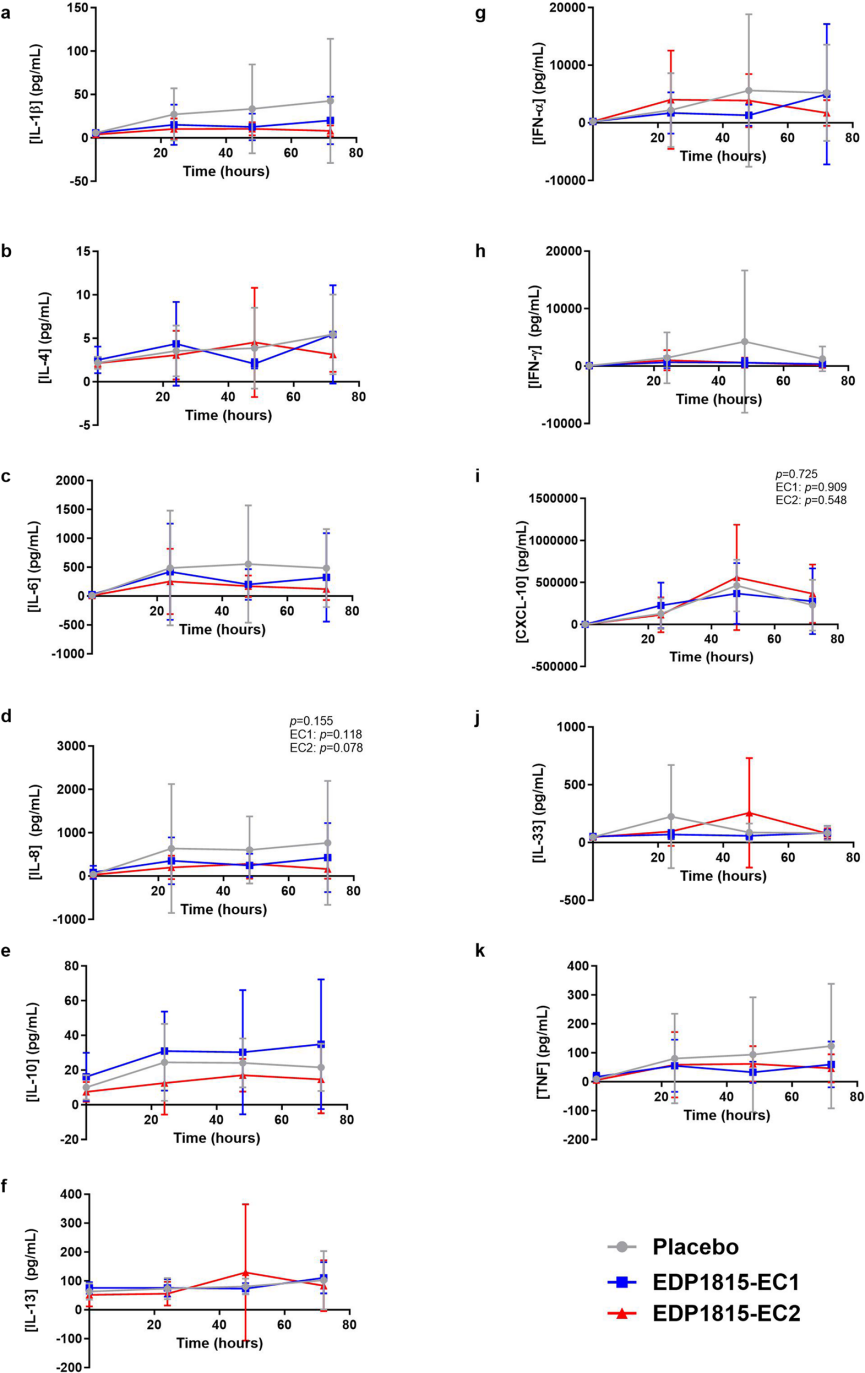
Treatment effect on cytokine influx in blister fluid during imiquimod challenge. **a**–**k **Cytokine concentrations in blister fluid of blisters induced on imiquimod-treated skin during the imiquimod challenge. CXCL-10, CXC-motif chemokine ligand 10; IFN, interferon; IL, interleukin; TNF, tumour necrosis factor. Mean (standard deviation). *p*-values refer to the following contrasts: upper *p*-value EDP1815 overall vs placebo; EC1 EDP1815-EC1 vs placebo; EC2 EDP1815-EC2 vs placebo

In subjects who had received placebo, the first imiquimod application resulted in an increase in basal flow, flare and erythema during the 24 h after application. The second and third imiquimod application (administered at the same place after 24 and 48 h) resulted also in a slight further increase in basal flow, flare and erythema, but the increase was much less steep than in the first 24 h. The flare even slightly decreased after 48 h of imiquimod application (Fig. 3). The volumes of collected blister fluid ranged from 0.0024 to 0.2079 mL per blister, with an average volume of 0.0618 mL per blister. In blister fluid, there was a consistent influx of inflammatory cells during imiquimod application in the placebo group, with a peak after 48 h of imiquimod application (Fig. 4). Accordingly, imiquimod application resulted in elevated blister cytokine levels in the placebo group. For IL-1β, IL-4, IL-6, IL-8, IL-10, IFN-α and TNF, this was a gradual increase over time. For IFN-γ, CXCL-10 and IL-33, there was a peak after 48 h of imiquimod application, similar as for the influx of inflammatory cells. For IL-13, there was only a very small increase in concentration after 72 h of imiquimod application (Fig. 5). There was no treatment effect on basal flow (EC1, *p* = 0.813; EC2, *p* = 0.612; overall* p* = 0.878), flare (EC1, *p* = 0.775; EC2, *p =* 0.558; overall* p =* 0.840) and erythema (EC1, *p* = 0.846; EC2, *p =* 0.369; overall* p* = 0.636) after 24 h, 48 h and 72 h of topical imiquimod exposure (Fig. 3 and Table S11). However, the imiquimod-induced immune reaction measured in suction blister fluid showed a pattern of EDP1815-dependent reduction in inflammatory cells over time in blister fluid. This pattern was consistent for all measured cell types: total cells, B cells, T cells, natural killer (NK) cells, cytotoxic T cells, T helper cells, monocytes and dendritic cells (Fig. 4 and Table S12). The treatment effect was significant for the number of neutrophils (EC1, *p* = 0.011; EC2, *p* = 0.802; overall* p* = 0.016) and granulocytes (EC1, *p* = 0.014; EC2: *p* = 0.809; overall* p* = 0.024). For EC1 compared to placebo, but not for EC2, the reduction in inflammatory cells was also significant in case of total cells (EC1, *p* = 0.046; EC2, *p* = 0.435; overall* p* = 0.128) and NK-cells (EC1, *p* = 0.046; EC2, *p* = 0.190; overall* p* = 0.128), and borderline significant for T cells (EC1, *p* = 0.052; EC2, *p* = 0.703; overall* p* = 0.117). The significance could not be calculated for intermediate monocytes, non classical monocytes and plasmacytoid dendritic cells due to a non-normal distribution of measurements. In the blister fluid of EDP1815-treated participants, imiquimod-induced cytokine levels were generally lower than in placebo-treated participants. This trend was visible for IL-1β, IL-6, IL-8, IL-10, IFN-γ and TNF (in case of IL-10 only for EDP1815 EC2-treated participants) but not for IL-4, IL-13, IL-33, IFN-α and CXCL-10 (Figs. 5 and S13). In EDP1815-EC1 treated subjects, there were higher levels of IL-10 in blister fluid than in the placebo group. Because of the high number of cytokine measurements below the limit of quantification, *p*-values could only be calculated for IL-8 (EC1, *p* = 0.118, EC2, *p* = 0.078; overall *p* = 0.155) and CXCL-10 (EC1, *p* = 0.909; EC2, *p* = 0.548; overall *p* = 0.725) (Fig. 5 and Table S13).

## Discussion

In this study, the immunomodulatory effects of EDP1815, a single-strain human commensal, were evaluated by means of two independent human immune challenge models. EDP1815 was safe and well-tolerated. Although no immunomodulatory effects on the KLH challenge were observed, EDP1815 had an immunomodulatory effect on the innate immune response as driven by the TLR7 agonist imiquimod.

A previous study hinted towards a potential modulatory effect of EDP1815 on the adaptive immune response, as investigated by KLH immunization and intradermal KLH skin challenge [16]. In this study, a trend of EDP1815-dependent inhibition of both the primary antigen response and DTH skin response was observed. The current study served to confirm or reject the potential immunomodulation by EDP1815 and to explore the impact of EDP1815 formulation. EDP1815 treatment did not alter KLH-specific antibody production or DTH response at any of the skin imaging endpoints. The immunization schedule and skin imaging in the current study differed from the approach used in the past study: volunteers were now immunized three times with KLH (instead of once), and KLH response monitoring of the skin was performed at 24 h after intradermal KLH injection (instead of after 48 h). These changes were implemented to improve the KLH response window but theoretically may have interfered in EDP1815 response detection, e.g. due to overstimulation by three immunizations compared to the mild immunosuppressive effect of EDP1815. However, the approach of the KLH challenge as used in the current study has been validated extensively, and results in the placebo group were as expected based on previous data (*to be published*) [17]. We conclude based on the generated data that EDP1815 does not interfere in the primary or DTH immune responses upon neoantigen exposure, independent of the formulation used. In general, it may be challenging to detect immunomodulatory effects of compounds like EDP1815 in humans, based on a neoantigen immunization. Firstly, baseline microbiome composition is variable between participants, hampering the detection of potentially mild immunomodulatory effects of a single-strain commensal such as EDP1815 [25]. Secondly, there is substantial interindividual variability in the immune response to neo-antigens like KLH [26]. Such sources of variability make it challenging to detect the effects of an immunomodulatory compound, even when compound responses in animal models are convincing, as was the case for EDP1815 in murine-based models [11–16].

As opposed to the results of the KLH challenge, reflecting adaptive immunity, EDP1815 showed a convincing immunomodulatory effect on TLR7-mediated innate immune responses, evaluated by inflammatory cells and cytokines in blister fluid on skin challenged with imiquimod. As demonstrated by the placebo group, responses to the imiquimod challenge were in accordance with previous research, except for an additional influx of neutrophils, TNF and IL-1β in blister fluid [21], probably related to differences in tape stripping procedures before imiquimod administration [20, 21]. Particularly formulation EDP1815-EC1 treatment consistently reduced the influx of inflammatory cells across all studied cell subsets. This effect reached a level of significance for total blister cells, NK cells, neutrophils and — as expected based on the results in neutrophils — granulocytes. The effects on T and B cells were borderline significant, but it should be taken into account that the study was not powered for imiquimod-based endpoints. The trend of reduced inflammation in EDP1815-treated participants (both EC1 and EC2) was observed for NFκB-driven cytokines IL-6, IL-8, IL-1β, IFN-γ and TNF, but not for differently regulated cytokines such as CXCL-10 or IFN-α. For the immunosuppressive cytokine IL-10, the results were different, as levels of this cytokine were lower in EDP1815-EC2 treated subjects compared to placebo, but increased in EDP1815-EC1 treated subjects. The difference in effect of EDP1815-EC1 and EDP1815-EC2 on IL-10 cannot be explained. However, it should be taken into account that in case of this specific cytokine, concentrations in blister fluid were relatively low compared to the concentrations of other cytokines. Taken together, these data suggest a suppression of imiquimod-driven skin inflammation by EDP1815 treatment. Suppression of skin inflammation in the same imiquimod challenge model was demonstrated earlier for prednisolone and an interleukin-1 receptor-associated kinase 4 (IRAK4) inhibitor [Ten Voorde *et al*, submitted for publication] [21].

While the invasive endpoints of the imiquimod challenge (cells, cytokines) showed a consistent immunomodulatory effect of EDP1815, there was no effect on imaging outcomes of this challenge (basal flow, flare, erythema). The discrepancy between EDP1815 effects based on the imaging and cellular outcomes following the imiquimod challenge is interesting and underlines the potential unrelatedness of TLR-driven vascular responses (probably iNOS- and eNOS-driven [27, 28]) and other immune responses. The results of an immunosuppressive effect of EDP1815 on the invasive endpoints of the imiquimod challenge are in line with the results of preclinical research showing a suppressive effect of EDP1815 on imiquimod-induced inflammation in mice [16]. In hindsight, powering the current study on the cellular endpoints of the imiquimod challenge instead of the imaging endpoints of the KLH challenge could have revealed more significant treatment effects. Additionally, measuring not only imaging outcomes but also invasive outcomes for the KLH challenge (skin cells, cytokines) could have revealed immunomodulatory effects of EDP1815 on adaptive immune responses, similar to the effects on the imiquimod challenge.

In this study, the difference in effect between two different enteric coatings was studied. It was hypothesized that the EC2 coating would have better effects than the EC1 coating, because of the earlier release and therefore higher exposure of EDP1815 to the gut epithelium by EC2 as compared to EC1. The majority of the leukocyte populations of the gut immune system are located in the duodenum and jejunum, hence earlier dissolution of the capsules should lead to more exposure to intra-epithelial lymphocytes (IELs) of the gut [29]. However, the results of our study were opposite and were not in line with this hypothesis, with a stronger effect of EC1 compared to EC2 on the influx of inflammatory cells in blister fluid during the imiquimod challenge, and comparable effects of EC1 and EC2 on cytokines. Possibly, there was still variation in the place of dissolution of the capsules within the EC1 and EC2 groups.

Although the efficacy of EDP1815 had been demonstrated in several preclinical studies [11–15], not all immunomodulatory effects could be reproduced in the current clinical study. This could be explained by several factors, including the inherent suboptimal translation of immune responses between mice and man, experimental conditions (preclinical studies are performed in a controlled environment, whereas human volunteers are constantly exposed to wide ranges of microorganisms), or a suboptimal EDP1815 dose evaluated in the clinical setting. Dose-response relationships of gut-targeting drugs are difficult to determine since monitoring of actual exposure is impossible. Given the observation that a dose of 8.0 × 10^11^ cells EDP1815 per day did result in a clinical effect in psoriasis patients, as opposed to the results of the current study, in hindsight, the used dose may have been too low to exert a significant effect on the KLH response.

Concluding, in this study an immunomodulatory effect of EDP1815, a single-strain preparation of *P. histicola*, was demonstrated. EDP1815, particularly as formulation EC1, caused reductions in imiquimod-induced inflammatory cells and cytokine responses in blister fluid, indicating an immunomodulatory effect on TLR7-driven innate immune reaction. These findings are in line with a recently conducted phase 2 clinical study in psoriasis patients that showed positive treatment effects of EDP1815 [Maslin et al., submitted for publication]. EDP1815 treatment also modulated imiquimod-driven T and B cell responses, suggesting a potential impact of the compound on adaptive immunity. EDP1815 treatment did not affect imiquimod-induced skin perfusion or erythema and had no effect on the adaptive immune response studied by the KLH challenge. Based on the observed immunomodulatory potential of EDP1815, the compound may be valuable for the long-term treatment of autoimmune diseases, such as psoriasis and eczema.

### Supplementary Information

Below is the link to the electronic supplementary material.

Supplementary file1 (DOCX 982 KB)

## Data Availability

No datasets were generated or analysed during the current study.
