# Supplementary material for: Immunomodulating effects of the single bacterial strain therapy EDP1815 on innate and adaptive immune challenge responses — a randomized, placebo-controlled clinical trial
Source: Immunol Res. 2024 May 15;72(4):776–87. doi: 10.1007/s12026-024-09484-7 (PMC11347467; doi:10.1007/s12026-024-09484-7)
Supplement: Supplementary file 1 — Supplementary file1 (DOCX 982 KB) [file 12026_2024_9484_MOESM1_ESM.docx]

**Supplementary Information** – ‘Immunomodulating effects of the single bacterial strain therapy *EDP1815* on innate and adaptive immune challenge responses – a randomized, placebo-controlled clinical trial’, *Immunologic Research*

Authors: Boukje C. Eveleens Maarse, Micha N. Ronner, Manon A.A. Jansen, Tessa Niemeyer-van der Kolk, Aliede E. in ’t Veld, Erica S. Klaassen, Saira Ahmad, Andrea Itano, Duncan McHale, Matthijs Moerland.

Corresponding author: M. Moerland, Centre for Human Drug Research, Zernikedreef 8, 2333 CL Leiden, The Netherlands. E-mail address: [mmoerland@chdr.nl](mailto:mmoerland@chdr.nl)

**List of inclusion and exclusion criteria**

Inclusion criteria

Participants are eligible to be included in the study only if all the following criteria apply:

1. Capable of giving signed informed consent which includes compliance with the requirements and restrictions listed in the informed consent form (ICF) and in this protocol. Obtained prior to any screening procedures and in accordance with national, local, institutional guidelines.
2. Age 18 to 45 years, inclusive.
3. Participant has a body mass index of 18 to 35 kg/m2, inclusive, at screening.
4. Contraception: a. Male participants: A male participant must agree to use contraception as indicated in Section 4.5.1 during their participation in this study and for a period of 90 days after the last dose and refrain from donating sperm during this period. b. Female participants: A female participant is eligible to participate if she is not pregnant, does not plan to become pregnant during the study, is not breastfeeding, and at least 1 of the following conditions applies: i. Not a woman of child-bearing potential (WOCBP) OR ii. A WOCBP who agrees to follow the contraceptive guidance during their participation in this study as indicated in Section 4.5.1 and for at least 30 days after last dose of Investigational Medicinal Product (IMP.)
5. CRP ≤ 10 (repeated testing is permitted).
6. The participant has clinical laboratory evaluations (including clinical chemistry, haematology,

and complete urine analysis) within the reference range for the testing laboratory, unless the results are deemed not to be clinically significant by the investigator (1 repeat test is permitted, unless more agreed with the sponsor medical monitor).

1. Fitzpatrick skin type I-III (Caucasian).
2. Participants who are overtly healthy as determined by medical evaluation including medical history, vital signs, physical examination, laboratory tests and ECGs at Screening and on Day -1.
3. The participant needs to have sufficient space in a refrigerator to store the IMP during the ambulant dosing phase.
4. The participant should be willing to take the IMP daily in the morning, to refrain from eating 2 hours before and 1 hour after dosing, and to refrain from drinking acidic drinks 1 hour before and 1 hour after dosing.
5. Participant can communicate well with the Investigator in the Dutch language and is willing to comply with the study restrictions.

Exclusion criteria:

1. Participant has an active infection (e.g. sepsis, pneumonia, abscess) or recurrent infection, or has had an infection requiring antibiotic treatment within 42 days prior to IMP administration.
2. Participant has active neoplastic disease or history of neoplastic disease within 5 years of Screening.
3. Impaired cardiac function or clinically significant cardiac diseases, including any of the following: a. Unstable angina or acute myocardial infarction within 6 months prior to Screening; b. Clinically significant heart disease (e.g. symptomatic congestive heart failure [e.g. >New York Heart Association [NYHA] Class 2]; uncontrolled arrhythmia or hypertension; history of labile hypertension or poor compliance with an antihypertensive regimen.
4. Participant with a positive screening result for hepatitis B surface antigen, anti-hepatitis B core, hepatitis C, or HIV.
5. Participants with gastrointestinal tract disease (e.g. short bowel syndrome, diarrhoea predominant irritable bowel syndrome [IBS], celiac disease) that could interfere with gastrointestinal delivery and/or transit time of the IMP.
6. Serious psychiatric or medical conditions that, in the opinion of the investigator, could interfere with treatment, compliance, or the ability to give consent.
7. The participant has taken any over-the-counter (OTC) medication (with the exception of paracetamol and anti-histamines) within 14 days prior to start of treatment (Day 1) or any prescription medications or nutraceuticals (e.g. supplements including high doses of probiotics and prebiotics, as usually found in capsules/tablets/powders) within 28 days prior to start of treatment (Day 1), or anticipates an inability to abstain from these products for the duration of the study period. Note that probiotic and prebiotic foods e.g. yoghurts that contain low doses are allowed.
8. Participant has received live attenuated vaccination within 42 days prior to Screening or intends to have vaccinations during the course of the study. Exception is SARS-CoV-2 vaccinations and boosters, which are allowed, with exception of day 50 until day 60.
9. The participant has used Aldara® (imiquimod cream) within 3 weeks prior to the baseline visit or plans to use it during the course of the study.
10. Previous known exposure to Immucothel® or KLH.
11. Participant has received any investigational drug or experimental procedure within 90 days or 5 half-lives, whichever is longer, prior to study intervention administration or participant was enrolled in an investigational drug or device study within 90 days prior to first IMP dosing.
12. The participant has a history of hypersensitivity or allergies to Prevotella (or Prevotella containing probiotics) including any associated excipients for EDP1815 or EDP2939, or has a history of hypersensitivity or allergies to placebo capsule/powder (magnesium stearate, microcrystalline cellulose, colloidal silicon dioxide, hydroxypropylmethylcellulose, or mannitol) or to the hard capsule shells (hydroxylpropylmethylcellulose and titanium dioxide), or has a known allergy against Alhydrogel®, or has a known allergy against Aldara® (imiquimod cream).
13. The participant has a hypersensitivity for dermatological marker at screening.
14. The participant has donated more than 400 mL of blood or blood products within 90 days prior to start of treatment (Day 1) or plans to donate blood during the study.
15. The participant has had an acute, clinically significant illness or major surgery within 30 days prior to screening.
16. The participant has any current and / or recurrent pathologically, clinically significant skin condition at the treatment area (i.e., atopic dermatitis), including tattoos. Treatment area includes the forearms and back.
17. Current (or within past 6 months) nicotine use in excess of 5 cigarettes per day, or unable not to smoke during the assessment visits.
18. History of abuse of addictive substances (alcohol, illegal substances) or current use of more than 14 units alcohol per week, drug abuse, or regular user of sedatives, hypnotics, tranquillisers, or any other addictive agent.
19. History of pathological scar formation (keloid, hypertrophic scar) or keloids or surgical scars in the target treatment area that in the opinion of the investigator, would limit or interfere with dosing and/or measurement in the trial.
20. Diagnosed with psoriasis.
21. History of skin cancer (i.e., basal cell carcinoma, squamous cell carcinoma, melanoma).
22. Tanning due to sunbathing, excessive sun exposure or a tanning booth within 3 weeks before start of treatment (Day 1) and for the duration of the study.
23. History of Schistosomiasis (infection with Schistosoma parasite).

**Table S1.** Flow cytometry antibody list.

| **Marker** | **Fluorophore** | **Supplier** | **Clone** |
| --- | --- | --- | --- |
| CD45 | BV570 | Biolegend | HI30 |
| CD3 | PerCP | Biolegend | OKT3 |
| CD4 | AF700 | Biolegend | OKT4 |
| CD8 | BV510 | Biolegend | SK1 |
| HLA-DR | KIRAVIA Blue | Biolegend | L243 |
| CD19 | PE-Cy7 | Biolegend | HIB19 |
| CD14 | BV605 | Biolegend | 63D3 |
| CD16 | APC-fire750 | Biolegend | 3G8 |
| CD66b | PE | Biolegend | 6/40C |
| CD56 | BV421 | Biolegend | HCD56 |
| CD123 | BV650 | Biolegend | HI30 |
| CD11c | APC | Biolegend | S-HCL-3 |
| Viability | PI | Miltenyi |  |

BV: brilliant violet; PE: phycoerythrin; PI: Propidium Iodide; APC: anti-allophycocyanin; PerCP: peridinin-chlorophyll protein; AF: AlexaFluor.

**
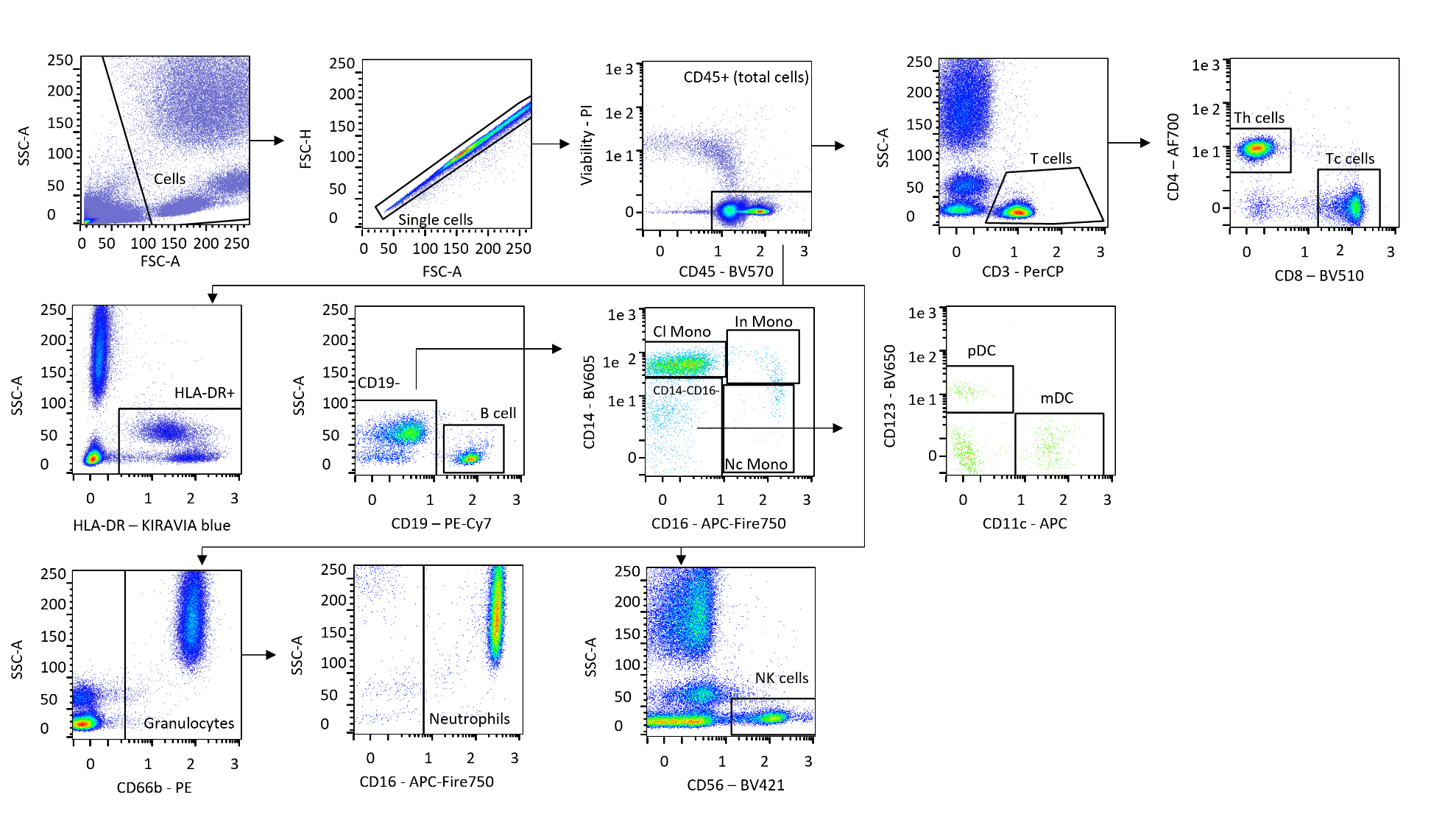
Figure S1.** Gating strategy of flow cytometry analysis of cells present in blister fluid. This gating strategy was created using a whole blood sample.

**Table S2.** Treatment compliance.

|  | EDP1815-EC1* (N=13) | EDP1815-EC2* (N=13) | Placebo (N=12) |
| --- | --- | --- | --- |
| Mean compliance (%) (SD) | 92.43 (26.78) | 92.95 (24.42) | 99.86 (0.49) |
| N ≥80% compliant | 12* | 12* | 12 |

SD: standard deviation. % of total doses (60) of EDP1815 or placebo taken by a participant.

*In both groups, one participant was excluded and treatment was discontinued from that timepoint onwards, resulting in one non-compliant participant per treatment group. Non-compliant participants were not included in pharmacodynamic analyses.

**Table S3. Adverse events.**

|  | EDP1815-EC1 (N=13) | EDP1815-EC2 (N=13) | | Placebo (N=12) |
| --- | --- | --- | --- | --- |
| All AEs | 12 (92.3) | | 12 (92.3) | 11 (91.7) |
| Treatment-related AEs | 10 (76.9) | | 6 (46.2) | 7 (58.3) |
| Severe AEs | 0 (0%) | | 0 (0%) | 0 (0%) |
| AEs leading to death | 0 (0%) | | 0 (0%) | 0 (0%) |
| SAEs | 0 (0%) | | 0 (0%) | 0 (0%) |
| Treatment-related SAEs | 0 (0%) | | 0 (0%) | 0 (0%) |
| AEs leading to discontinuation of study treatment | 1 (7.7%) | | 0 (0%) | 0 (0%) |
| AEs leading to discontinuation from study | 1 (7.7%) | | 0 (0%) | 0 (0%) |

AE: Adverse event. SAE: Serious adverse event.

**Table S4.** **Summary of adverse events.** Summary of number of subjects with treatment-emergent adverse events (TEAEs) by treatment, system organ class (SOC), preferred term (PT) and severity. Subjects with more than one TEAE within a SOC or PT are counted only once per SOC or PT.

|  | **EDP1815 EC1 (N=13)** | | | **EDP1815 EC2 (N=13)** | | | **Placebo (N=12)** | | |
| --- | --- | --- | --- | --- | --- | --- | --- | --- | --- |
| **System Organ Class/  Preferred Term** | **Mild N** | **Moderate N** | **Severe N** | **Mild N** | **Moderate N** | **Severe N** | **Mild N** | **Moderate N** | **Severe N** |
| ANY EVENTS | 12 | 1 | - | 12 | 1 | - | 11 | 1 | - |
|  |  |  |  |  |  |  |  |  |  |
| EAR AND LABYRINTH DISORDERS | - | - | - | 1 | - | - | - | - | - |
| External ear inflammation | - | - | - | 1 | - | - | - | - | - |
|  |  |  |  |  |  |  |  |  |  |
| EYE DISORDERS | - | - | - | 1 | - | - | - | - | - |
| Eye pruritus | - | - | - | 1 | - | - | - | - | - |
|  |  |  |  |  |  |  |  |  |  |
| GASTROINTESTINAL DISORDERS | 6 | 1 | - | 3 | - | - | 4 | - | - |
| Abdominal distension | 1 | - | - | - | - | - | 1 | - | - |
| Abdominal pain | - | - | - | 1 | - | - | - | - | - |
| Abdominal pain upper | 2 | - | - | 1 | - | - | - | - | - |
| Constipation | - | 1 | - | - | - | - | - | - | - |
| Diarrhoea | 3 | - | - | - | - | - | 2 | - | - |
| Eructation | - | - | - | - | - | - | 1 | - | - |
| Mouth ulceration | 1 | - | - | - | - | - | - | - | - |
| Nausea | 2 | - | - | 3 | - | - | 2 | - | - |
| Toothache | 1 | - | - | - | - | - | - | - | - |
|  |  |  |  |  |  |  |  |  |  |
| GENERAL DISORDERS AND ADMINISTRATION SITE CONDITIONS | 11 | - | - | 10 | - | - | 10 | - | - |
| Administration site pruritus | - | - | - | 1 | - | - | - | - | - |
| Application site pruritus | 1 | - | - | 2 | - | - | 3 | - | - |
| Application site warmth | - | - | - | 1 | - | - | - | - | - |
| Asthenia | - | - | - | 1 | - | - | - | - | - |
| Fatigue | 1 | - | - | 1 | - | - | 2 | - | - |
| Feeling cold | - | - | - | - | - | - | 1 | - | - |
| Feeling hot | - | - | - | 1 | - | - | - | - | - |
| Injection site erythema | 2 | - | - | 1 | - | - | 2 | - | - |
| Injection site pain | 6 | - | - | 5 | - | - | 2 | - | - |
| Injection site paraesthesia | - | - | - | 1 | - | - | - | - | - |
| Injection site pruritus | 6 | - | - | 6 | - | - | 6 | - | - |
|  | | | | | | | | | |

|  | **EDP1815 EC1 (N=13)** | | | **EDP1815 EC2 (N=13)** | | | **Placebo (N=12)** | | |
| --- | --- | --- | --- | --- | --- | --- | --- | --- | --- |
| **System Organ Class/  Preferred Term** | **Mild N** | **Moderate N** | **Severe N** | **Mild N** | **Moderate N** | **Severe N** | **Mild N** | **Moderate N** | **Severe N** |
| Injection site reaction | 3 | - | - | 1 | - | - | 2 | - | - |
| Injection site swelling | 2 | - | - | - | - | - | 1 | - | - |
| Injection site warmth | 1 | - | - | - | - | - | 1 | - | - |
| Tenderness | 1 | - | - | - | - | - | 1 | - | - |
|  |  |  |  |  |  |  |  |  |  |
| IMMUNE SYSTEM DISORDERS | 1 | - | - | - | - | - | - | - | - |
| Allergy to arthropod sting | 1 | - | - | - | - | - | - | - | - |
|  |  |  |  |  |  |  |  |  |  |
| INFECTIONS AND INFESTATIONS | 3 | - | - | 1 | 1 | - | 3 | 1 | - |
| COVID-19 | 1 | - | - | - | 1 | - | - | - | - |
| Chlamydial infection | - | - | - | - | - | - | 1 | - | - |
| Folliculitis | 1 | - | - | - | - | - | - | - | - |
| Gastroenteritis | - | - | - | 1 | - | - | 1 | 1 | - |
| Oral herpes | - | - | - | - | - | - | 1 | - | - |
| Pharyngitis | - | - | - | - | - | - | 1 | - | - |
| Rhinitis | 1 | - | - | - | - | - | 1 | - | - |
|  |  |  |  |  |  |  |  |  |  |
| INJURY, POISONING AND PROCEDURAL COMPLICATIONS | 1 | - | - | 1 | - | - | 2 | - | - |
| Contusion | - | - | - | 1 | - | - | - | - | - |
| Scratch | 1 | - | - | - | - | - | 1 | - | - |
| Sunburn | - | - | - | - | - | - | 1 | - | - |
| Traumatic haematoma | - | - | - | - | - | - | 1 | - | - |
|  |  |  |  |  |  |  |  |  |  |
| INVESTIGATIONS | 1 | - | - | 1 | - | - | - | - | - |
| Hepatic enzyme increased | 1 | - | - | 1 | - | - | - | - | - |
|  |  |  |  |  |  |  |  |  |  |
| MUSCULOSKELETAL AND CONNECTIVE TISSUE DISORDERS | 2 | - | - | 2 | - | - | 5 | - | - |
| Back pain | - | - | - | - | - | - | 2 | - | - |
| Muscle spasms | - | - | - | - | - | - | 1 | - | - |
| Musculoskeletal stiffness | - | - | - | 1 | - | - | - | - | - |
|  | | | | | | | | | |

|  | | | | | | | | | | |
| --- | --- | --- | --- | --- | --- | --- | --- | --- | --- | --- |
|  | **EDP1815 EC1 (N=13)** | | | **EDP1815 EC2 (N=13)** | | | **Placebo (N=12)** | | |  |
| **System Organ Class/  Preferred Term** | **Mild N** | **Moderate N** | **Severe N** | **Mild N** | **Moderate N** | **Severe N** | **Mild N** | **Moderate N** | **Severe N** |  |
| Myalgia | 2 | - | - | 1 | - | - | 3 | - | - |  |
|  |  |  |  |  |  |  |  |  |  |  |
| NERVOUS SYSTEM DISORDERS | 3 | - | - | 5 | - | - | 5 | - | - |  |
| Dizziness | 1 | - | - | - | - | - | 1 | - | - |  |
| Headache | 3 | - | - | 5 | - | - | 2 | - | - |  |
| Migraine | - | - | - | - | - | - | 1 | - | - |  |
| Presyncope | - | - | - | - | - | - | 1 | - | - |  |
| Tension headache | - | - | - | 1 | - | - | - | - | - |  |
|  |  |  |  |  |  |  |  |  |  |  |
| RENAL AND URINARY DISORDERS | 1 | - | - | - | - | - | - | - | - |  |
| Bladder pain | 1 | - | - | - | - | - | - | - | - |  |
|  |  |  |  |  |  |  |  |  |  |  |
| REPRODUCTIVE SYSTEM AND BREAST DISORDERS | 1 | - | - | - | - | - | - | - | - |  |
| Dysmenorrhoea | 1 | - | - | - | - | - | - | - | - |  |
|  |  |  |  |  |  |  |  |  |  |  |
| RESPIRATORY, THORACIC AND MEDIASTINAL DISORDERS | 1 | - | - | 1 | - | - | 2 | - | - |  |
| Cough | - | - | - | 1 | - | - | 1 | - | - |  |
| Oropharyngeal pain | - | - | - | - | - | - | 1 | - | - |  |
| Rhinalgia | 1 | - | - | - | - | - | - | - | - |  |
|  |  |  |  |  |  |  |  |  |  |  |
| SKIN AND SUBCUTANEOUS TISSUE DISORDERS | 5 | - | - | 2 | - | - | 1 | - | - |  |
| Acne | 1 | - | - | - | - | - | - | - | - |  |
| Alopecia | - | - | - | 1 | - | - | - | - | - |  |
| Erythema | - | - | - | 1 | - | - | 1 | - | - |  |
| Granuloma annulare | 1 | - | - | - | - | - | - | - | - |  |
| Pruritus | 1 | - | - | - | - | - | - | - | - |  |
| Rash erythematous | 1 | - | - | - | - | - | - | - | - |  |
| Scab | 2 | - | - | - | - | - | - | - | - |  |
|  |  |  |  |  |  |  |  |  |  |  |
| VASCULAR DISORDERS | - | - | - | 2 | - | - | - | - | - |  |
| Haematoma | - | - | - | 2 | - | - | - | - | - |  |
|  | | | | | | | | | |  |

**Table S5.** **Drug relatedness of adverse events.** Summary of number of subjects with treatment-emergent adverse events (TEAEs) by treatment, system organ class (SOC), preferred term (PT) and EDP1815 relatedness. Subjects with more than one TEAE within a SOC or PT are counted only once per SOC or PT. AEs known to be caused by keyhole limpet haemocyanin (KLH) or imiquimod administration are classified as unrelated.

|  | **EDP1815 EC1 (N=13)** | | | **EDP1815 EC2 (N=13)** | | | **Placebo (N=12)** | | |
| --- | --- | --- | --- | --- | --- | --- | --- | --- | --- |
| **System Organ Class/  Preferred Term** | **PROBABLE N** | **POSSIBLE N** | **UNRELATED N** | **PROBABLE N** | **POSSIBLE N** | **UNRELATED N** | **PROBABLE N** | **POSSIBLE N** | **UNRELATED N** |
| ANY EVENTS | - | 10 | 12 | - | 6 | 12 | - | 7 | 10 |
|  |  |  |  |  |  |  |  |  |  |
| EAR AND LABYRINTH DISORDERS | - | - | - | - | - | 1 | - | - | - |
| External ear inflammation | - | - | - | - | - | 1 | - | - | - |
|  |  |  |  |  |  |  |  |  |  |
| EYE DISORDERS | - | - | - | - | 1 | - | - | - | - |
| Eye pruritus | - | - | - | - | 1 | - | - | - | - |
|  |  |  |  |  |  |  |  |  |  |
| GASTROINTESTINAL DISORDERS | - | 7 | 1 | - | 2 | 1 | - | 4 | - |
| Abdominal distension | - | 1 | - | - | - | - | - | 1 | - |
| Abdominal pain | - | - | - | - | 1 | - | - | - | - |
| Abdominal pain upper | - | 2 | - | - | 1 | - | - | - | - |
| Constipation | - | 1 | - | - | - | - | - | - | - |
| Diarrhoea | - | 3 | - | - | - | - | - | 2 | - |
| Eructation | - | - | - | - | - | - | - | 1 | - |
| Mouth ulceration | - | 1 | - | - | - | - | - | - | - |
| Nausea | - | 2 | - | - | 2 | 1 | - | 2 | - |
| Toothache | - | - | 1 | - | - | - | - | - | - |
|  |  |  |  |  |  |  |  |  |  |
| GENERAL DISORDERS AND ADMINISTRATION SITE CONDITIONS | - | 1 | 10 | - | 1 | 10 | - | 2 | 10 |
| Administration site pruritus | - | - | - | - | - | 1 | - | - | - |
| Application site pruritus | - | - | 1 | - | - | 2 | - | - | 3 |
| Application site warmth | - | - | - | - | - | 1 | - | - | - |
| Asthenia | - | - | - | - | 1 | - | - | - | - |
| Fatigue | - | 1 | - | - | - | 1 | - | 2 | - |
| Feeling cold | - | - | - | - | - | - | - | 1 | - |
| Feeling hot | - | - | - | - | - | 1 | - | - | - |
| Injection site erythema | - | - | 2 | - | - | 1 | - | - | 2 |
| Injection site pain | - | - | 6 | - | - | 5 | - | - | 2 |
| Injection site paraesthesia | - | - | - | - | - | 1 | - | - | - |
| Injection site pruritus | - | - | 6 | - | - | 6 | - | - | 6 |
|  | | | | | | | | | |
| Injection site reaction | - | - | 3 | - | - | 1 | - | - | 2 |
| Injection site swelling | - | - | 2 | - | - | - | - | - | 1 |
| Injection site warmth | - | - | 1 | - | - | - | - | - | 1 |
| Tenderness | - | - | 1 | - | - | - | - | - | 1 |
|  |  |  |  |  |  |  |  |  |  |
| IMMUNE SYSTEM DISORDERS | - | - | 1 | - | - | - | - | - | - |
| Allergy to arthropod sting | - | - | 1 | - | - | - | - | - | - |
|  |  |  |  |  |  |  |  |  |  |
| INFECTIONS AND INFESTATIONS | - | 1 | 2 | - | - | 2 | - | - | 4 |
| COVID-19 | - | - | 1 | - | - | 1 | - | - | - |
| Chlamydial infection | - | - | - | - | - | - | - | - | 1 |
| Folliculitis | - | 1 | - | - | - | - | - | - | - |
| Gastroenteritis | - | - | - | - | - | 1 | - | - | 2 |
| Oral herpes | - | - | - | - | - | - | - | - | 1 |
| Pharyngitis | - | - | - | - | - | - | - | - | 1 |
| Rhinitis | - | - | 1 | - | - | - | - | - | 1 |
|  |  |  |  |  |  |  |  |  |  |
| INJURY, POISONING AND PROCEDURAL COMPLICATIONS | - | - | 1 | - | - | 1 | - | - | 2 |
| Contusion | - | - | - | - | - | 1 | - | - | - |
| Scratch | - | - | 1 | - | - | - | - | - | 1 |
| Sunburn | - | - | - | - | - | - | - | - | 1 |
| Traumatic haematoma | - | - | - | - | - | - | - | - | 1 |
|  |  |  |  |  |  |  |  |  |  |
| INVESTIGATIONS | - | 1 | - | - | 1 | - | - | - | - |
| Hepatic enzyme increased | - | 1 | - | - | 1 | - | - | - | - |
|  |  |  |  |  |  |  |  |  |  |
| MUSCULOSKELETAL AND CONNECTIVE TISSUE DISORDERS | - | 1 | 2 | - | - | 2 | - | 2 | 3 |
| Back pain | - | - | - | - | - | - | - | 1 | 1 |
| Muscle spasms | - | - | - | - | - | - | - | 1 | - |
| Musculoskeletal stiffness | - | - | - | - | - | 1 | - | - | - |
|  | | | | | | | | | |
| Myalgia | - | 1 | 2 | - | - | 1 | - | 1 | 2 |
|  |  |  |  |  |  |  |  |  |  |
| NERVOUS SYSTEM DISORDERS | - | 3 | 1 | - | 5 | 1 | - | 1 | 4 |
| Dizziness | - | - | 1 | - | - | - | - | - | 1 |
| Headache | - | 3 | - | - | 5 | 1 | - | 1 | 1 |
| Migraine | - | - | - | - | - | - | - | - | 1 |
| Presyncope | - | - | - | - | - | - | - | - | 1 |
| Tension headache | - | - | - | - | - | 1 | - | - | - |
|  |  |  |  |  |  |  |  |  |  |
| RENAL AND URINARY DISORDERS | - | - | 1 | - | - | - | - | - | - |
| Bladder pain | - | - | 1 | - | - | - | - | - | - |
|  |  |  |  |  |  |  |  |  |  |
| REPRODUCTIVE SYSTEM AND BREAST DISORDERS | - | - | 1 | - | - | - | - | - | - |
| Dysmenorrhoea | - | - | 1 | - | - | - | - | - | - |
|  |  |  |  |  |  |  |  |  |  |
| RESPIRATORY, THORACIC AND MEDIASTINAL DISORDERS | - | - | 1 | - | 1 | - | - | 2 | - |
| Cough | - | - | - | - | 1 | - | - | 1 | - |
| Oropharyngeal pain | - | - | - | - | - | - | - | 1 | - |
| Rhinalgia | - | - | 1 | - | - | - | - | - | - |
|  |  |  |  |  |  |  |  |  |  |
| SKIN AND SUBCUTANEOUS TISSUE DISORDERS | - | 3 | 2 | - | 1 | 1 | - | - | 1 |
| Acne | - | 1 | - | - | - | - | - | - | - |
| Alopecia | - | - | - | - | 1 | - | - | - | - |
| Erythema | - | - | - | - | - | 1 | - | - | 1 |
| Granuloma annulare | - | 1 | - | - | - | - | - | - | - |
| Pruritus | - | - | 1 | - | - | - | - | - | - |
| Rash erythematous | - | 1 | - | - | - | - | - | - | - |
| Scab | - | - | 2 | - | - | - | - | - | - |
|  |  |  |  |  |  |  |  |  |  |
| VASCULAR DISORDERS  Haematoma | -  - | -  - | -  - | -  - | 1  1 | 1  1 | -  - | -  - | -  - |
| Haematoma- | | | | | | | | | |

**Table S6.** **Summary of laboratory chemistry results.** Chemistry results are presented by treatment and protocol time, including change from baseline (baseline: Day -1). ALAT: alanine aminotransferase, AlkPhos: alkaline phosphatase, ASAT: aspartate aminotransferase, CRP: C-reactive protein, Conjug: conjugated, FU: follow-up visit, GammaGT: gamma-glutamyltransferase, LDH: lactate dehydrogenase, SD: standard deviation, SE: standard error, min: minimum, max: maximum, SCR: screening visit, TotProtein: total protein.

|  | | | | **Result** | | | | | | | **\| Change from baseline** | | | | | | |
| --- | --- | --- | --- | --- | --- | --- | --- | --- | --- | --- | --- | --- | --- | --- | --- | --- | --- |
| **Treatment** | **Protocol time** | **Label** | **Unit** | **N** | **mean** | **SE** | **SD** | **median** | **min** | **max** | **N** | **mean** | **SE** | **SD** | **median** | **min** | **max** |
| EDP1815 EC1 | SCR | ALAT | U/L | 13 | 23.9 | 3.1 | 11.0 | 22 | 12 | 52 |  |  |  |  |  |  |  |
|  |  | Albumin | g/L | 13 | 48.5 | 0.6 | 2.3 | 49 | 43 | 52 |  |  |  |  |  |  |  |
|  |  | AlkPhos | U/L | 13 | 66.6 | 4.1 | 15.0 | 63 | 45 | 93 |  |  |  |  |  |  |  |
|  |  | ASAT | U/L | 13 | 24.0 | 1.3 | 4.7 | 23 | 18 | 34 |  |  |  |  |  |  |  |
|  |  | CRP | mg/L | 11 | 1.691 | 0.440 | 1.460 | 1.10 | 0.30 | 4.00 |  |  |  |  |  |  |  |
|  |  | Conjug Bilirubin | umol/L | 1 | 10.0 |  |  | 10 | 10 | 10 |  |  |  |  |  |  |  |
|  |  | Total Bilirubin | umol/L | 13 | 11.4 | 3.0 | 10.8 | 8 | 5 | 46 |  |  |  |  |  |  |  |
|  |  | Calcium | mmol/L | 13 | 2.329 | 0.019 | 0.068 | 2.34 | 2.19 | 2.45 |  |  |  |  |  |  |  |
|  |  | Creatinin | umol/L | 13 | 72.2 | 4.1 | 14.9 | 68 | 56 | 108 |  |  |  |  |  |  |  |
|  |  | GammaGT | U/L | 13 | 15.7 | 2.1 | 7.6 | 14 | 8 | 38 |  |  |  |  |  |  |  |
|  |  | Glucose | mmol/L | 13 | 4.60 | 0.06 | 0.23 | 4.5 | 4.3 | 5.0 |  |  |  |  |  |  |  |
|  |  | LDH | U/L | 13 | 169.5 | 5.9 | 21.2 | 171 | 139 | 203 |  |  |  |  |  |  |  |
|  |  | Phosphate | mmol/L | 13 | 1.089 | 0.038 | 0.136 | 1.06 | 0.91 | 1.41 |  |  |  |  |  |  |  |
|  |  | Potassium | mmol/L | 13 | 4.25 | 0.06 | 0.23 | 4.3 | 3.8 | 4.6 |  |  |  |  |  |  |  |
|  |  | Sodium | mmol/L | 13 | 139.4 | 0.5 | 1.8 | 140 | 136 | 141 |  |  |  |  |  |  |  |
|  |  | TotProtein | g/L | 13 | 69.2 | 0.8 | 2.7 | 70 | 64 | 73 |  |  |  |  |  |  |  |
|  |  | Triglycerides | mmol/L | 13 | 0.818 | 0.074 | 0.266 | 0.87 | 0.42 | 1.19 |  |  |  |  |  |  |  |
|  |  | Urea | mmol/L | 13 | 4.38 | 0.28 | 1.00 | 3.9 | 3.2 | 6.3 |  |  |  |  |  |  |  |
|  |  | Serum uric acid | mmol/L | 13 | 0.255 | 0.013 | 0.048 | 0.25 | 0.19 | 0.34 |  |  |  |  |  |  |  |
|  | Day -1 | ALAT | U/L | 13 | 24.1 | 4.4 | 16.0 | 19 | 9 | 61 |  |  |  |  |  |  |  |
|  |  | Albumin | g/L | 13 | 47.5 | 0.8 | 3.0 | 47 | 43 | 54 |  |  |  |  |  |  |  |
|  |  | AlkPhos | U/L | 13 | 67.5 | 4.8 | 17.1 | 66 | 45 | 97 |  |  |  |  |  |  |  |
|  |  | ASAT | U/L | 13 | 23.5 | 2.6 | 9.4 | 20 | 15 | 52 |  |  |  |  |  |  |  |
|  |  | CRP | mg/L | 11 | 1.473 | 0.486 | 1.611 | 0.60 | 0.30 | 5.60 |  |  |  |  |  |  |  |
|  |  | Conjug Bilirubin | umol/L | 2 | 7.0 | 2.0 | 2.8 | 7 | 5 | 9 |  |  |  |  |  |  |  |
|  |  | Total Bilirubin | umol/L | 13 | 10.8 | 2.1 | 7.6 | 8 | 4 | 32 |  |  |  |  |  |  |  |
|  |  | Calcium | mmol/L | 13 | 2.310 | 0.018 | 0.066 | 2.30 | 2.23 | 2.45 |  |  |  |  |  |  |  |
|  |  | Creatinin | umol/L | 13 | 70.1 | 3.1 | 11.3 | 66 | 58 | 94 |  |  |  |  |  |  |  |
|  |  | GammaGT | U/L | 13 | 17.3 | 2.5 | 9.0 | 15 | 10 | 45 |  |  |  |  |  |  |  |
|  |  | Glucose | mmol/L | 13 | 4.72 | 0.11 | 0.40 | 4.7 | 4.1 | 5.4 |  |  |  |  |  |  |  |
|  |  | LDH | U/L | 13 | 161.5 | 5.6 | 20.0 | 157 | 137 | 208 |  |  |  |  |  |  |  |
|  |  | Phosphate | mmol/L | 13 | 1.102 | 0.030 | 0.108 | 1.07 | 0.92 | 1.25 |  |  |  |  |  |  |  |
|  |  | Potassium | mmol/L | 13 | 4.22 | 0.06 | 0.22 | 4.1 | 3.9 | 4.5 |  |  |  |  |  |  |  |
| EDP1815 EC1 | Day -1 | Sodium | mmol/L | 13 | 139.6 | 0.5 | 1.8 | 139 | 138 | 143 |  |  |  |  |  |  |  |
|  |  | TotProtein | g/L | 13 | 67.0 | 1.3 | 4.7 | 65 | 62 | 78 |  |  |  |  |  |  |  |
|  |  | Triglycerides | mmol/L | 13 | 0.780 | 0.079 | 0.284 | 0.75 | 0.39 | 1.41 |  |  |  |  |  |  |  |
|  |  | Urea | mmol/L | 13 | 4.31 | 0.32 | 1.16 | 4.2 | 2.5 | 6.6 |  |  |  |  |  |  |  |
|  |  | Serum uric acid | mmol/L | 13 | 0.256 | 0.024 | 0.085 | 0.26 | 0.14 | 0.47 |  |  |  |  |  |  |  |
|  | Day 22 | ALAT | U/L | 12 | 19.7 | 2.8 | 9.6 | 16 | 11 | 43 | 12 | -2.1 | 2.1 | 7.3 | -2 | -18 | 8 |
|  |  | Albumin | g/L | 12 | 46.7 | 0.9 | 3.1 | 47 | 43 | 52 | 12 | -0.7 | 0.9 | 3.3 | -2 | -6 | 5 |
|  |  | AlkPhos | U/L | 12 | 65.4 | 3.9 | 13.6 | 67 | 49 | 91 | 12 | 0.2 | 1.3 | 4.7 | 2 | -8 | 5 |
|  |  | ASAT | U/L | 12 | 20.7 | 1.5 | 5.3 | 20 | 14 | 33 | 12 | -2.3 | 2.7 | 9.4 | -2 | -28 | 10 |
|  |  | CRP | mg/L | 8 | 1.913 | 0.643 | 1.819 | 1.15 | 0.50 | 5.80 | 8 | 0.025 | 0.819 | 2.317 | -0.30 | -2.50 | 5.30 |
|  |  | Conjug Bilirubin | umol/L | 0 |  |  |  |  |  |  | 0 |  |  |  |  |  |  |
|  |  | Total Bilirubin | umol/L | 12 | 9.4 | 1.4 | 4.8 | 9 | 3 | 17 | 12 | -1.8 | 1.8 | 6.1 | -1 | -20 | 5 |
|  |  | Calcium | mmol/L | 12 | 2.315 | 0.022 | 0.077 | 2.33 | 2.18 | 2.43 | 12 | 0.001 | 0.022 | 0.077 | 0.02 | -0.12 | 0.12 |
|  |  | Creatinin | umol/L | 12 | 70.3 | 2.3 | 7.8 | 72 | 60 | 81 | 12 | -0.4 | 1.9 | 6.5 | 1 | -14 | 7 |
|  |  | GammaGT | U/L | 12 | 16.0 | 2.8 | 9.7 | 13 | 10 | 44 | 12 | -1.5 | 0.5 | 1.8 | -2 | -4 | 3 |
|  |  | LDH | U/L | 12 | 162.5 | 4.2 | 14.6 | 161 | 142 | 198 | 12 | 1.9 | 3.9 | 13.6 | 2 | -18 | 25 |
|  |  | Phosphate | mmol/L | 12 | 0.965 | 0.039 | 0.135 | 0.97 | 0.75 | 1.21 | 12 | -0.125 | 0.051 | 0.175 | -0.10 | -0.36 | 0.22 |
|  |  | Potassium | mmol/L | 12 | 4.18 | 0.05 | 0.18 | 4.2 | 3.8 | 4.4 | 12 | -0.04 | 0.06 | 0.22 | 0.0 | -0.3 | 0.4 |
|  |  | Sodium | mmol/L | 12 | 139.6 | 0.5 | 1.7 | 140 | 136 | 142 | 12 | -0.1 | 0.6 | 2.0 | 0 | -4 | 2 |
|  |  | TotProtein | g/L | 12 | 67.7 | 0.8 | 2.8 | 68 | 63 | 72 | 12 | 0.8 | 1.2 | 4.2 | 2 | -6 | 7 |
|  |  | Triglycerides | mmol/L | 12 | 0.901 | 0.092 | 0.318 | 0.93 | 0.44 | 1.30 | 12 | 0.088 | 0.075 | 0.261 | 0.12 | -0.28 | 0.53 |
|  |  | Urea | mmol/L | 12 | 4.34 | 0.36 | 1.26 | 4.2 | 2.9 | 7.7 | 12 | -0.12 | 0.33 | 1.14 | -0.1 | -3.2 | 1.5 |
|  |  | Serum uric acid | mmol/L | 12 | 0.279 | 0.017 | 0.058 | 0.27 | 0.19 | 0.38 | 12 | 0.018 | 0.019 | 0.068 | 0.01 | -0.09 | 0.19 |
|  | Day 57 | ALAT | U/L | 12 | 22.8 | 4.1 | 14.1 | 20 | 10 | 65 | 12 | 1.1 | 4.8 | 16.8 | 3 | -33 | 41 |
|  |  | Albumin | g/L | 12 | 48.2 | 1.0 | 3.3 | 48 | 43 | 54 | 12 | 0.8 | 1.2 | 4.3 | 1 | -7 | 7 |
|  |  | AlkPhos | U/L | 12 | 66.2 | 4.0 | 14.0 | 66 | 46 | 82 | 12 | 0.9 | 2.3 | 8.1 | 2 | -18 | 16 |
|  |  | ASAT | U/L | 12 | 21.5 | 3.0 | 10.6 | 18 | 15 | 54 | 12 | -1.5 | 4.1 | 14.2 | -2 | -34 | 32 |
|  |  | CRP | mg/L | 10 | 1.400 | 0.394 | 1.247 | 0.85 | 0.30 | 4.10 | 10 | -0.170 | 0.413 | 1.307 | 0.05 | -3.20 | 1.40 |
|  |  | Conjug Bilirubin | umol/L | 1 | 7.0 |  |  | 7 | 7 | 7 | 1 | -2.0 |  |  | -2 | -2 | -2 |
|  |  | Total Bilirubin | umol/L | 12 | 10.3 | 1.5 | 5.2 | 10 | 4 | 24 | 12 | -0.9 | 1.1 | 3.7 | 0 | -8 | 4 |
|  |  | Calcium | mmol/L | 12 | 2.306 | 0.032 | 0.111 | 2.30 | 2.15 | 2.51 | 12 | -0.008 | 0.030 | 0.105 | -0.02 | -0.17 | 0.15 |
|  |  | Creatinin | umol/L | 12 | 71.9 | 2.5 | 8.6 | 70 | 61 | 87 | 12 | 1.2 | 1.7 | 5.9 | 0 | -7 | 9 |
|  |  | GammaGT | U/L | 12 | 18.3 | 5.1 | 17.6 | 14 | 7 | 73 | 12 | 0.8 | 2.6 | 9.0 | -2 | -7 | 28 |
|  | | | | | | | | | | | | | | | | | |

|  | | | | | | | | | | | | | | | | | | |
| --- | --- | --- | --- | --- | --- | --- | --- | --- | --- | --- | --- | --- | --- | --- | --- | --- | --- | --- |
|  | | | | **Result** | | | | | | | **\| Change from baseline** | | | | | | |  |
| **Treatment** | **Protocol time** | **Label** | **Unit** | **N** | **mean** | **SE** | **SD** | **median** | **min** | **max** | **N** | **mean** | **SE** | **SD** | **median** | **min** | **max** |  |
| EDP1815 EC1 | Day 57 | LDH | U/L | 12 | 162.8 | 7.1 | 24.5 | 158 | 132 | 221 | 12 | 2.2 | 5.2 | 18.0 | 6 | -48 | 20 |  |
|  |  | Phosphate | mmol/L | 12 | 0.965 | 0.044 | 0.152 | 0.92 | 0.80 | 1.27 | 12 | -0.125 | 0.029 | 0.100 | -0.11 | -0.27 | 0.04 |  |
|  |  | Potassium | mmol/L | 12 | 4.29 | 0.06 | 0.22 | 4.3 | 4.0 | 4.7 | 12 | 0.08 | 0.06 | 0.22 | 0.2 | -0.3 | 0.3 |  |
|  |  | Sodium | mmol/L | 12 | 139.4 | 0.4 | 1.5 | 139 | 138 | 143 | 12 | -0.3 | 0.6 | 2.1 | 0 | -4 | 2 |  |
|  |  | TotProtein | g/L | 12 | 69.3 | 1.3 | 4.4 | 69 | 63 | 77 | 12 | 2.4 | 1.6 | 5.6 | 4 | -9 | 9 |  |
|  |  | Triglycerides | mmol/L | 12 | 0.917 | 0.144 | 0.499 | 0.87 | 0.42 | 2.23 | 12 | 0.104 | 0.112 | 0.389 | 0.18 | -0.56 | 0.82 |  |
|  |  | Urea | mmol/L | 12 | 4.16 | 0.31 | 1.07 | 4.1 | 2.7 | 6.2 | 12 | -0.30 | 0.14 | 0.50 | -0.4 | -1.0 | 0.7 |  |
|  |  | Serum uric acid | mmol/L | 12 | 0.266 | 0.018 | 0.064 | 0.25 | 0.17 | 0.41 | 12 | 0.005 | 0.009 | 0.030 | 0.01 | -0.06 | 0.05 |  |
|  | Day 60 | ALAT | U/L | 12 | 22.4 | 3.4 | 11.7 | 19 | 12 | 54 | 12 | 0.7 | 4.0 | 13.7 | 3 | -26 | 30 |  |
|  |  | Albumin | g/L | 12 | 47.3 | 0.8 | 2.6 | 48 | 44 | 51 | 12 | 0.0 | 0.8 | 2.8 | 0 | -6 | 4 |  |
|  |  | AlkPhos | U/L | 12 | 65.5 | 4.2 | 14.4 | 69 | 46 | 84 | 12 | 0.3 | 2.2 | 7.5 | 0 | -13 | 16 |  |
|  |  | ASAT | U/L | 12 | 20.5 | 1.7 | 6.0 | 20 | 13 | 36 | 12 | -2.5 | 3.1 | 10.6 | -2 | -30 | 14 |  |
|  |  | CRP | mg/L | 11 | 1.691 | 0.541 | 1.794 | 0.80 | 0.30 | 5.40 | 10 | 0.260 | 0.529 | 1.673 | 0.05 | -3.00 | 3.60 |  |
|  |  | Conjug Bilirubin | umol/L | 1 | 6.0 |  |  | 6 | 6 | 6 | 1 | -3.0 |  |  | -3 | -3 | -3 |  |
|  |  | Total Bilirubin | umol/L | 12 | 9.8 | 1.7 | 5.8 | 8 | 4 | 23 | 12 | -1.5 | 1.2 | 4.0 | -2 | -9 | 5 |  |
|  |  | Calcium | mmol/L | 12 | 2.309 | 0.024 | 0.085 | 2.31 | 2.14 | 2.45 | 12 | -0.005 | 0.020 | 0.068 | -0.01 | -0.12 | 0.08 |  |
|  |  | Creatinin | umol/L | 12 | 71.8 | 1.6 | 5.7 | 71 | 65 | 84 | 12 | 1.1 | 1.8 | 6.3 | 2 | -10 | 8 |  |
|  |  | GammaGT | U/L | 12 | 17.8 | 5.1 | 17.7 | 14 | 8 | 73 | 12 | 0.3 | 2.6 | 9.0 | -2 | -7 | 28 |  |
|  |  | LDH | U/L | 12 | 153.5 | 5.9 | 20.3 | 147 | 128 | 202 | 12 | -7.1 | 4.8 | 16.8 | -6 | -52 | 16 |  |
|  |  | Phosphate | mmol/L | 12 | 1.031 | 0.034 | 0.120 | 1.04 | 0.79 | 1.27 | 12 | -0.059 | 0.029 | 0.100 | -0.05 | -0.28 | 0.09 |  |
|  |  | Potassium | mmol/L | 12 | 4.25 | 0.05 | 0.19 | 4.2 | 4.0 | 4.6 | 12 | 0.03 | 0.08 | 0.28 | 0.0 | -0.4 | 0.5 |  |
|  |  | Sodium | mmol/L | 12 | 139.3 | 0.4 | 1.4 | 140 | 137 | 141 | 12 | -0.4 | 0.4 | 1.3 | -1 | -2 | 3 |  |
|  |  | TotProtein | g/L | 12 | 68.1 | 0.9 | 3.2 | 68 | 63 | 75 | 12 | 1.3 | 1.4 | 4.9 | 3 | -10 | 6 |  |
|  |  | Triglycerides | mmol/L | 12 | 0.908 | 0.081 | 0.279 | 0.85 | 0.61 | 1.38 | 12 | 0.096 | 0.084 | 0.290 | 0.12 | -0.30 | 0.63 |  |
|  |  | Urea | mmol/L | 12 | 4.36 | 0.35 | 1.22 | 3.7 | 3.1 | 6.6 | 12 | -0.10 | 0.25 | 0.85 | 0.0 | -1.4 | 1.3 |  |
|  |  | Serum uric acid | mmol/L | 12 | 0.256 | 0.016 | 0.054 | 0.25 | 0.16 | 0.35 | 12 | -0.005 | 0.013 | 0.044 | 0.01 | -0.12 | 0.05 |  |
|  | Day 60 | Glucose | mmol/L | 12 | 4.60 | 0.14 | 0.49 | 4.6 | 3.7 | 5.6 | 12 | -0.12 | 0.08 | 0.28 | -0.2 | -0.5 | 0.3 |  |
|  | FU | ALAT | U/L | 12 | 20.4 | 2.9 | 9.9 | 18 | 9 | 45 |  |  |  |  |  |  |  |  |
|  |  | Albumin | g/L | 12 | 47.7 | 0.7 | 2.5 | 48 | 44 | 53 |  |  |  |  |  |  |  |  |
|  |  | AlkPhos | U/L | 12 | 65.4 | 3.8 | 13.3 | 66 | 45 | 85 |  |  |  |  |  |  |  |  |
|  |  | ASAT | U/L | 12 | 23.0 | 2.4 | 8.4 | 22 | 13 | 47 |  |  |  |  |  |  |  |  |
|  |  | CRP | mg/L | 9 | 1.267 | 0.368 | 1.103 | 0.70 | 0.30 | 3.00 |  |  |  |  |  |  |  |  |
|  |  | Conjug Bilirubin | umol/L | 2 | 7.0 | 1.0 | 1.4 | 7 | 6 | 8 |  |  |  |  |  |  |  |  |
|  | | | | | | | | | | | | | | | | | |  |

|  | | | | | | | | | | | | | | | | | | |
| --- | --- | --- | --- | --- | --- | --- | --- | --- | --- | --- | --- | --- | --- | --- | --- | --- | --- | --- |
|  | | | | **Result** | | | | | | | **\| Change from baseline** | | | | | | |  |
| **Treatment** | **Protocol time** | **Label** | **Unit** | **N** | **mean** | **SE** | **SD** | **median** | **min** | **max** | **N** | **mean** | **SE** | **SD** | **median** | **min** | **max** |  |
| EDP1815 EC1 | FU | Total Bilirubin | umol/L | 12 | 11.6 | 2.5 | 8.5 | 9 | 4 | 36 |  |  |  |  |  |  |  |  |
|  |  | Calcium | mmol/L | 12 | 2.328 | 0.028 | 0.098 | 2.30 | 2.20 | 2.53 |  |  |  |  |  |  |  |  |
|  |  | Creatinin | umol/L | 12 | 70.3 | 3.1 | 10.8 | 69 | 52 | 89 |  |  |  |  |  |  |  |  |
|  |  | GammaGT | U/L | 12 | 18.8 | 5.6 | 19.5 | 14 | 9 | 80 |  |  |  |  |  |  |  |  |
|  |  | LDH | U/L | 12 | 182.8 | 9.2 | 32.0 | 179 | 141 | 239 |  |  |  |  |  |  |  |  |
|  |  | Phosphate | mmol/L | 12 | 1.058 | 0.027 | 0.093 | 1.03 | 0.94 | 1.23 |  |  |  |  |  |  |  |  |
|  |  | Potassium | mmol/L | 12 | 4.49 | 0.07 | 0.24 | 4.4 | 4.2 | 5.1 |  |  |  |  |  |  |  |  |
|  |  | Sodium | mmol/L | 12 | 139.7 | 0.6 | 2.1 | 140 | 136 | 142 |  |  |  |  |  |  |  |  |
|  |  | TotProtein | g/L | 12 | 69.5 | 1.1 | 3.9 | 69 | 64 | 77 |  |  |  |  |  |  |  |  |
|  |  | Triglycerides | mmol/L | 12 | 0.871 | 0.106 | 0.368 | 0.84 | 0.35 | 1.75 |  |  |  |  |  |  |  |  |
|  |  | Urea | mmol/L | 12 | 3.89 | 0.28 | 0.96 | 3.8 | 2.7 | 5.3 |  |  |  |  |  |  |  |  |
|  |  | Serum uric acid | mmol/L | 12 | 0.257 | 0.019 | 0.067 | 0.23 | 0.18 | 0.36 |  |  |  |  |  |  |  |  |
| EDP1815 EC2 | SCR | ALAT | U/L | 13 | 21.2 | 3.1 | 11.1 | 17 | 10 | 43 |  |  |  |  |  |  |  |  |
|  |  | Albumin | g/L | 13 | 49.2 | 0.9 | 3.4 | 48 | 44 | 55 |  |  |  |  |  |  |  |  |
|  |  | AlkPhos | U/L | 13 | 77.8 | 7.8 | 28.3 | 75 | 46 | 154 |  |  |  |  |  |  |  |  |
|  |  | ASAT | U/L | 13 | 21.4 | 2.2 | 7.9 | 18 | 11 | 36 |  |  |  |  |  |  |  |  |
|  |  | CRP | mg/L | 11 | 1.291 | 0.484 | 1.605 | 0.70 | 0.30 | 5.90 |  |  |  |  |  |  |  |  |
|  |  | Conjug Bilirubin | umol/L | 3 | 6.7 | 0.7 | 1.2 | 6 | 6 | 8 |  |  |  |  |  |  |  |  |
|  |  | Total Bilirubin | umol/L | 13 | 11.8 | 2.0 | 7.2 | 10 | 4 | 25 |  |  |  |  |  |  |  |  |
|  |  | Calcium | mmol/L | 13 | 2.342 | 0.029 | 0.106 | 2.32 | 2.21 | 2.54 |  |  |  |  |  |  |  |  |
|  |  | Creatinin | umol/L | 13 | 72.4 | 4.2 | 15.1 | 73 | 53 | 97 |  |  |  |  |  |  |  |  |
|  |  | GammaGT | U/L | 13 | 14.6 | 2.1 | 7.5 | 11 | 4 | 32 |  |  |  |  |  |  |  |  |
|  |  | Glucose | mmol/L | 13 | 4.76 | 0.10 | 0.38 | 4.7 | 4.3 | 5.3 |  |  |  |  |  |  |  |  |
|  |  | LDH | U/L | 13 | 164.9 | 6.8 | 24.6 | 162 | 135 | 217 |  |  |  |  |  |  |  |  |
|  |  | Phosphate | mmol/L | 13 | 1.180 | 0.038 | 0.138 | 1.19 | 0.90 | 1.35 |  |  |  |  |  |  |  |  |
|  |  | Potassium | mmol/L | 13 | 4.48 | 0.13 | 0.48 | 4.4 | 3.7 | 5.5 |  |  |  |  |  |  |  |  |
|  |  | Sodium | mmol/L | 13 | 140.1 | 0.6 | 2.1 | 140 | 137 | 144 |  |  |  |  |  |  |  |  |
|  |  | TotProtein | g/L | 13 | 70.7 | 1.0 | 3.5 | 71 | 66 | 78 |  |  |  |  |  |  |  |  |
|  |  | Triglycerides | mmol/L | 13 | 1.085 | 0.282 | 1.017 | 0.83 | 0.32 | 4.21 |  |  |  |  |  |  |  |  |
|  |  | Urea | mmol/L | 13 | 4.53 | 0.39 | 1.39 | 4.3 | 2.4 | 7.1 |  |  |  |  |  |  |  |  |
|  |  | Serum uric acid | mmol/L | 13 | 0.310 | 0.018 | 0.066 | 0.31 | 0.22 | 0.47 |  |  |  |  |  |  |  |  |
|  | Day -1 | ALAT | U/L | 13 | 22.5 | 2.4 | 8.5 | 21 | 11 | 39 |  |  |  |  |  |  |  |  |
|  |  | Albumin | g/L | 13 | 46.1 | 0.8 | 2.9 | 45 | 41 | 52 |  |  |  |  |  |  |  |  |
|  | | | | | | | | | | | | | | | | | |  |

|  | | | | | | | | | | | | | | | | | | |
| --- | --- | --- | --- | --- | --- | --- | --- | --- | --- | --- | --- | --- | --- | --- | --- | --- | --- | --- |
|  | | | | **Result** | | | | | | | **\| Change from baseline** | | | | | | |  |
| **Treatment** | **Protocol time** | **Label** | **Unit** | **N** | **mean** | **SE** | **SD** | **median** | **min** | **max** | **N** | **mean** | **SE** | **SD** | **median** | **min** | **max** |  |
| EDP1815 EC2 | Day -1 | AlkPhos | U/L | 13 | 73.3 | 7.1 | 25.8 | 74 | 41 | 143 |  |  |  |  |  |  |  |  |
|  |  | ASAT | U/L | 13 | 24.2 | 2.2 | 7.8 | 23 | 16 | 43 |  |  |  |  |  |  |  |  |
|  |  | CRP | mg/L | 11 | 0.964 | 0.223 | 0.741 | 0.60 | 0.30 | 2.80 |  |  |  |  |  |  |  |  |
|  |  | Conjug Bilirubin | umol/L | 2 | 7.0 | 1.0 | 1.4 | 7 | 6 | 8 |  |  |  |  |  |  |  |  |
|  |  | Total Bilirubin | umol/L | 12 | 11.2 | 1.7 | 5.9 | 10 | 4 | 24 |  |  |  |  |  |  |  |  |
|  |  | Calcium | mmol/L | 13 | 2.265 | 0.020 | 0.073 | 2.26 | 2.15 | 2.39 |  |  |  |  |  |  |  |  |
|  |  | Creatinin | umol/L | 13 | 68.2 | 4.1 | 14.9 | 69 | 46 | 95 |  |  |  |  |  |  |  |  |
|  |  | GammaGT | U/L | 13 | 14.0 | 1.6 | 5.7 | 13 | 6 | 24 |  |  |  |  |  |  |  |  |
|  |  | Glucose | mmol/L | 13 | 4.65 | 0.09 | 0.34 | 4.6 | 4.2 | 5.4 |  |  |  |  |  |  |  |  |
|  |  | LDH | U/L | 13 | 169.8 | 7.1 | 25.7 | 170 | 124 | 222 |  |  |  |  |  |  |  |  |
|  |  | Phosphate | mmol/L | 13 | 1.110 | 0.035 | 0.127 | 1.11 | 0.94 | 1.39 |  |  |  |  |  |  |  |  |
|  |  | Potassium | mmol/L | 13 | 4.24 | 0.06 | 0.22 | 4.3 | 3.8 | 4.7 |  |  |  |  |  |  |  |  |
|  |  | Sodium | mmol/L | 13 | 140.2 | 0.2 | 0.7 | 140 | 139 | 142 |  |  |  |  |  |  |  |  |
|  |  | TotProtein | g/L | 13 | 66.5 | 0.9 | 3.2 | 66 | 62 | 71 |  |  |  |  |  |  |  |  |
|  |  | Triglycerides | mmol/L | 13 | 0.865 | 0.146 | 0.527 | 0.69 | 0.39 | 2.02 |  |  |  |  |  |  |  |  |
|  |  | Urea | mmol/L | 13 | 5.13 | 0.35 | 1.25 | 5.3 | 3.2 | 7.0 |  |  |  |  |  |  |  |  |
|  |  | Serum uric acid | mmol/L | 13 | 0.312 | 0.021 | 0.077 | 0.31 | 0.19 | 0.50 |  |  |  |  |  |  |  |  |
|  | Day 22 | ALAT | U/L | 12 | 17.7 | 2.0 | 6.8 | 17 | 10 | 28 | 12 | -5.3 | 1.3 | 4.5 | -4 | -15 | 0 |  |
|  |  | Albumin | g/L | 12 | 47.5 | 0.7 | 2.3 | 48 | 44 | 51 | 12 | 1.4 | 0.9 | 3.1 | 1 | -5 | 7 |  |
|  |  | AlkPhos | U/L | 12 | 76.6 | 6.6 | 22.9 | 77 | 40 | 128 | 12 | 1.8 | 2.4 | 8.2 | 1 | -15 | 17 |  |
|  |  | ASAT | U/L | 12 | 20.6 | 1.5 | 5.1 | 21 | 13 | 27 | 12 | -4.3 | 2.0 | 7.0 | -3 | -25 | 1 |  |
|  |  | CRP | mg/L | 10 | 1.070 | 0.335 | 1.060 | 0.65 | 0.30 | 3.80 | 10 | 0.040 | 0.140 | 0.443 | 0.00 | -0.70 | 1.00 |  |
|  |  | Conjug Bilirubin | umol/L | 1 | 9.0 |  |  | 9 | 9 | 9 | 0 |  |  |  |  |  |  |  |
|  |  | Total Bilirubin | umol/L | 11 | 12.2 | 3.1 | 10.3 | 8 | 4 | 40 | 11 | 0.9 | 2.6 | 8.6 | -2 | -8 | 25 |  |
|  |  | Calcium | mmol/L | 12 | 2.323 | 0.026 | 0.089 | 2.34 | 2.15 | 2.46 | 12 | 0.058 | 0.025 | 0.086 | 0.07 | -0.06 | 0.22 |  |
|  |  | Creatinin | umol/L | 12 | 71.5 | 4.4 | 15.1 | 73 | 47 | 104 | 12 | 2.7 | 2.0 | 6.9 | 4 | -14 | 12 |  |
|  |  | GammaGT | U/L | 12 | 12.5 | 1.6 | 5.4 | 12 | 4 | 24 | 12 | -1.5 | 0.7 | 2.3 | -1 | -7 | 1 |  |
|  |  | LDH | U/L | 12 | 171.8 | 6.0 | 20.7 | 165 | 147 | 212 | 12 | -0.2 | 3.6 | 12.4 | -6 | -12 | 30 |  |
|  |  | Phosphate | mmol/L | 12 | 1.103 | 0.058 | 0.200 | 1.16 | 0.70 | 1.42 | 12 | -0.007 | 0.039 | 0.135 | -0.03 | -0.24 | 0.23 |  |
|  |  | Potassium | mmol/L | 12 | 4.46 | 0.11 | 0.37 | 4.5 | 3.6 | 4.9 | 12 | 0.23 | 0.11 | 0.40 | 0.2 | -0.8 | 0.8 |  |
|  |  | Sodium | mmol/L | 12 | 140.1 | 0.5 | 1.6 | 140 | 137 | 143 | 12 | -0.2 | 0.5 | 1.6 | -1 | -3 | 3 |  |
|  |  | TotProtein | g/L | 12 | 69.1 | 1.0 | 3.6 | 70 | 64 | 76 | 12 | 2.9 | 1.5 | 5.1 | 2 | -7 | 13 |  |
|  |  | Triglycerides | mmol/L | 12 | 0.982 | 0.137 | 0.475 | 0.82 | 0.63 | 2.30 | 12 | 0.078 | 0.080 | 0.277 | 0.16 | -0.49 | 0.39 |  |
|  | | | | | | | | | | | | | | | | | |  |

|  | | | | | | | | | | | | | | | | | | |
| --- | --- | --- | --- | --- | --- | --- | --- | --- | --- | --- | --- | --- | --- | --- | --- | --- | --- | --- |
|  | | | | **Result** | | | | | | | **\| Change from baseline** | | | | | | |  |
| **Treatment** | **Protocol time** | **Label** | **Unit** | **N** | **mean** | **SE** | **SD** | **median** | **min** | **max** | **N** | **mean** | **SE** | **SD** | **median** | **min** | **max** |  |
| EDP1815 EC2 | Day 22 | Urea | mmol/L | 12 | 4.96 | 0.39 | 1.36 | 5.0 | 3.2 | 7.4 | 12 | -0.27 | 0.29 | 1.01 | -0.1 | -2.5 | 1.0 |  |
|  |  | Serum uric acid | mmol/L | 12 | 0.299 | 0.021 | 0.073 | 0.30 | 0.18 | 0.46 | 12 | -0.014 | 0.011 | 0.039 | -0.02 | -0.07 | 0.08 |  |
|  | Day 57 | ALAT | U/L | 12 | 21.4 | 3.6 | 12.4 | 16 | 12 | 55 | 12 | -1.6 | 3.0 | 10.5 | -1 | -14 | 22 |  |
|  |  | Albumin | g/L | 12 | 47.3 | 0.7 | 2.3 | 48 | 43 | 51 | 12 | 1.2 | 0.8 | 2.7 | 2 | -3 | 4 |  |
|  |  | AlkPhos | U/L | 12 | 77.3 | 7.5 | 26.1 | 78 | 45 | 143 | 12 | 2.4 | 1.3 | 4.4 | 3 | -6 | 10 |  |
|  |  | ASAT | U/L | 12 | 23.7 | 3.9 | 13.3 | 21 | 12 | 62 | 12 | -1.2 | 4.2 | 14.5 | -4 | -25 | 35 |  |
|  |  | CRP | mg/L | 9 | 0.967 | 0.246 | 0.738 | 0.80 | 0.30 | 2.40 | 8 | -0.100 | 0.223 | 0.630 | -0.20 | -1.00 | 1.00 |  |
|  |  | Conjug Bilirubin | umol/L | 3 | 7.3 | 0.9 | 1.5 | 7 | 6 | 9 | 1 | -2.0 |  |  | -2 | -2 | -2 |  |
|  |  | Total Bilirubin | umol/L | 12 | 13.8 | 3.0 | 10.5 | 10 | 4 | 41 | 11 | 3.3 | 2.4 | 8.1 | 2 | -4 | 26 |  |
|  |  | Calcium | mmol/L | 12 | 2.358 | 0.022 | 0.075 | 2.36 | 2.20 | 2.47 | 12 | 0.094 | 0.025 | 0.087 | 0.08 | -0.02 | 0.27 |  |
|  |  | Creatinin | umol/L | 12 | 70.0 | 4.8 | 16.6 | 71 | 46 | 102 | 12 | 1.2 | 1.8 | 6.2 | 1 | -9 | 10 |  |
|  |  | GammaGT | U/L | 12 | 13.3 | 1.6 | 5.5 | 13 | 6 | 25 | 12 | -0.8 | 0.7 | 2.6 | -1 | -5 | 5 |  |
|  |  | LDH | U/L | 12 | 170.8 | 6.2 | 21.3 | 169 | 133 | 218 | 12 | -1.2 | 7.7 | 26.8 | -2 | -33 | 63 |  |
|  |  | Phosphate | mmol/L | 12 | 1.069 | 0.033 | 0.113 | 1.04 | 0.90 | 1.27 | 12 | -0.041 | 0.020 | 0.069 | -0.04 | -0.16 | 0.08 |  |
|  |  | Potassium | mmol/L | 12 | 4.43 | 0.07 | 0.25 | 4.4 | 4.1 | 4.8 | 12 | 0.19 | 0.11 | 0.38 | 0.3 | -0.6 | 0.7 |  |
|  |  | Sodium | mmol/L | 12 | 140.4 | 0.5 | 1.6 | 141 | 137 | 142 | 12 | 0.2 | 0.5 | 1.6 | 1 | -3 | 2 |  |
|  |  | TotProtein | g/L | 12 | 68.5 | 1.0 | 3.6 | 69 | 62 | 74 | 12 | 2.3 | 1.1 | 3.7 | 3 | -4 | 7 |  |
|  |  | Triglycerides | mmol/L | 12 | 1.438 | 0.233 | 0.809 | 1.24 | 0.47 | 2.70 | 12 | 0.533 | 0.140 | 0.484 | 0.45 | -0.06 | 1.52 |  |
|  |  | Urea | mmol/L | 12 | 4.44 | 0.30 | 1.05 | 4.5 | 2.5 | 5.9 | 12 | -0.78 | 0.36 | 1.26 | -1.0 | -2.2 | 1.7 |  |
|  |  | Serum uric acid | mmol/L | 12 | 0.282 | 0.021 | 0.073 | 0.27 | 0.19 | 0.47 | 12 | -0.032 | 0.013 | 0.044 | -0.04 | -0.10 | 0.06 |  |
|  | Day 60 | ALAT | U/L | 12 | 21.1 | 3.0 | 10.4 | 18 | 10 | 44 | 12 | -1.9 | 2.5 | 8.8 | -2 | -15 | 12 |  |
|  |  | Albumin | g/L | 12 | 47.6 | 0.8 | 2.7 | 48 | 42 | 51 | 12 | 1.5 | 0.6 | 2.2 | 2 | -3 | 6 |  |
|  |  | AlkPhos | U/L | 12 | 76.3 | 7.4 | 25.6 | 75 | 43 | 140 | 12 | 1.4 | 1.3 | 4.4 | -1 | -4 | 10 |  |
|  |  | ASAT | U/L | 12 | 21.3 | 2.1 | 7.3 | 21 | 12 | 35 | 12 | -3.5 | 2.7 | 9.3 | -4 | -26 | 8 |  |
|  |  | CRP | mg/L | 9 | 1.000 | 0.201 | 0.604 | 0.80 | 0.40 | 2.10 | 9 | -0.100 | 0.152 | 0.456 | -0.20 | -0.70 | 0.90 |  |
|  |  | Conjug Bilirubin | umol/L | 2 | 6.5 | 0.5 | 0.7 | 7 | 6 | 7 | 1 | -1.0 |  |  | -1 | -1 | -1 |  |
|  |  | Total Bilirubin | umol/L | 12 | 11.1 | 2.0 | 6.8 | 9 | 3 | 24 | 11 | 0.5 | 1.1 | 3.6 | 0 | -4 | 9 |  |
|  |  | Calcium | mmol/L | 12 | 2.323 | 0.026 | 0.090 | 2.33 | 2.14 | 2.45 | 12 | 0.058 | 0.020 | 0.070 | 0.06 | -0.05 | 0.21 |  |
|  |  | Creatinin | umol/L | 12 | 70.6 | 3.6 | 12.4 | 69 | 50 | 94 | 12 | 1.8 | 1.7 | 5.9 | 4 | -14 | 7 |  |
|  |  | GammaGT | U/L | 12 | 13.3 | 1.8 | 6.3 | 13 | 5 | 26 | 12 | -0.7 | 0.7 | 2.3 | -1 | -3 | 4 |  |
|  |  | LDH | U/L | 12 | 163.0 | 7.2 | 25.1 | 160 | 120 | 194 | 12 | -8.9 | 7.2 | 24.9 | -14 | -38 | 38 |  |
|  |  | Phosphate | mmol/L | 12 | 1.160 | 0.048 | 0.168 | 1.09 | 0.98 | 1.48 | 12 | 0.050 | 0.042 | 0.144 | 0.05 | -0.22 | 0.35 |  |
|  |  | Potassium | mmol/L | 12 | 4.34 | 0.09 | 0.31 | 4.4 | 3.6 | 4.8 | 12 | 0.11 | 0.09 | 0.32 | 0.1 | -0.5 | 0.8 |  |
|  | | | | | | | | | | | | | | | | | |  |

|  | | | | | | | | | | | | | | | | | | |
| --- | --- | --- | --- | --- | --- | --- | --- | --- | --- | --- | --- | --- | --- | --- | --- | --- | --- | --- |
|  | | | | **Result** | | | | | | | **\| Change from baseline** | | | | | | |  |
| **Treatment** | **Protocol time** | **Label** | **Unit** | **N** | **mean** | **SE** | **SD** | **median** | **min** | **max** | **N** | **mean** | **SE** | **SD** | **median** | **min** | **max** |  |
| EDP1815 EC2 | Day 60 | Sodium | mmol/L | 12 | 140.5 | 0.4 | 1.3 | 141 | 139 | 142 | 12 | 0.3 | 0.5 | 1.6 | 0 | -3 | 2 |  |
|  |  | TotProtein | g/L | 12 | 68.3 | 0.7 | 2.5 | 68 | 65 | 73 | 12 | 2.1 | 0.9 | 3.0 | 2 | -2 | 9 |  |
|  |  | Triglycerides | mmol/L | 12 | 1.063 | 0.191 | 0.663 | 0.95 | 0.38 | 2.65 | 12 | 0.159 | 0.120 | 0.414 | 0.11 | -0.69 | 0.66 |  |
|  |  | Urea | mmol/L | 12 | 4.64 | 0.35 | 1.21 | 4.6 | 2.7 | 7.4 | 12 | -0.58 | 0.32 | 1.12 | -0.7 | -2.1 | 1.0 |  |
|  |  | Serum uric acid | mmol/L | 12 | 0.283 | 0.021 | 0.074 | 0.27 | 0.19 | 0.48 | 12 | -0.030 | 0.010 | 0.035 | -0.02 | -0.10 | 0.01 |  |
|  | Day 60 | Glucose | mmol/L | 12 | 4.64 | 0.11 | 0.40 | 4.5 | 4.2 | 5.3 | 12 | -0.01 | 0.11 | 0.38 | 0.0 | -0.8 | 0.7 |  |
|  | FU | ALAT | U/L | 13 | 25.5 | 7.1 | 25.4 | 18 | 9 | 106 |  |  |  |  |  |  |  |  |
|  |  | Albumin | g/L | 13 | 48.3 | 0.9 | 3.1 | 49 | 42 | 54 |  |  |  |  |  |  |  |  |
|  |  | AlkPhos | U/L | 13 | 75.2 | 6.8 | 24.5 | 75 | 43 | 136 |  |  |  |  |  |  |  |  |
|  |  | ASAT | U/L | 13 | 45.7 | 25.3 | 91.1 | 20 | 14 | 348 |  |  |  |  |  |  |  |  |
|  |  | CRP | mg/L | 11 | 7.936 | 5.859 | 19.433 | 0.70 | 0.30 | 66.00 |  |  |  |  |  |  |  |  |
|  |  | Conjug Bilirubin | umol/L | 2 | 6.5 | 0.5 | 0.7 | 7 | 6 | 7 |  |  |  |  |  |  |  |  |
|  |  | Total Bilirubin | umol/L | 13 | 12.1 | 1.8 | 6.5 | 10 | 6 | 29 |  |  |  |  |  |  |  |  |
|  |  | Calcium | mmol/L | 13 | 2.365 | 0.031 | 0.111 | 2.41 | 2.17 | 2.54 |  |  |  |  |  |  |  |  |
|  |  | Creatinin | umol/L | 13 | 73.2 | 4.7 | 16.8 | 71 | 52 | 112 |  |  |  |  |  |  |  |  |
|  |  | GammaGT | U/L | 13 | 13.8 | 1.9 | 7.0 | 12 | 7 | 33 |  |  |  |  |  |  |  |  |
|  |  | LDH | U/L | 13 | 182.5 | 25.2 | 90.8 | 161 | 125 | 479 |  |  |  |  |  |  |  |  |
|  |  | Phosphate | mmol/L | 13 | 1.158 | 0.053 | 0.191 | 1.15 | 0.88 | 1.53 |  |  |  |  |  |  |  |  |
|  |  | Potassium | mmol/L | 13 | 4.52 | 0.09 | 0.32 | 4.5 | 4.1 | 5.1 |  |  |  |  |  |  |  |  |
|  |  | Sodium | mmol/L | 13 | 139.9 | 0.4 | 1.6 | 140 | 137 | 142 |  |  |  |  |  |  |  |  |
|  |  | TotProtein | g/L | 13 | 69.7 | 0.7 | 2.5 | 69 | 66 | 75 |  |  |  |  |  |  |  |  |
|  |  | Triglycerides | mmol/L | 13 | 0.979 | 0.138 | 0.497 | 0.86 | 0.27 | 2.03 |  |  |  |  |  |  |  |  |
|  |  | Urea | mmol/L | 13 | 4.72 | 0.45 | 1.63 | 4.4 | 2.4 | 8.0 |  |  |  |  |  |  |  |  |
|  |  | Serum uric acid | mmol/L | 13 | 0.291 | 0.016 | 0.058 | 0.28 | 0.23 | 0.45 |  |  |  |  |  |  |  |  |
| Placebo | SCR | ALAT | U/L | 12 | 24.2 | 3.6 | 12.6 | 21 | 12 | 59 |  |  |  |  |  |  |  |  |
|  |  | Albumin | g/L | 12 | 51.6 | 0.7 | 2.6 | 51 | 48 | 56 |  |  |  |  |  |  |  |  |
|  |  | AlkPhos | U/L | 12 | 61.8 | 5.7 | 19.6 | 58 | 36 | 109 |  |  |  |  |  |  |  |  |
|  |  | ASAT | U/L | 12 | 21.0 | 1.9 | 6.7 | 20 | 12 | 32 |  |  |  |  |  |  |  |  |
|  |  | CRP | mg/L | 12 | 1.350 | 0.521 | 1.805 | 0.55 | 0.30 | 6.00 |  |  |  |  |  |  |  |  |
|  |  | Conjug Bilirubin | umol/L | 1 | 6.0 |  |  | 6 | 6 | 6 |  |  |  |  |  |  |  |  |
|  |  | Total Bilirubin | umol/L | 12 | 11.3 | 1.4 | 4.8 | 10 | 5 | 21 |  |  |  |  |  |  |  |  |
|  |  | Calcium | mmol/L | 12 | 2.378 | 0.019 | 0.065 | 2.37 | 2.29 | 2.47 |  |  |  |  |  |  |  |  |
|  |  | Creatinin | umol/L | 12 | 72.6 | 3.4 | 11.9 | 74 | 53 | 99 |  |  |  |  |  |  |  |  |
|  | | | | | | | | | | | | | | | | | |  |

|  | | | | | | | | | | | | | | | | | | |
| --- | --- | --- | --- | --- | --- | --- | --- | --- | --- | --- | --- | --- | --- | --- | --- | --- | --- | --- |
|  | | | | **Result** | | | | | | | **\| Change from baseline** | | | | | | |  |
| **Treatment** | **Protocol time** | **Label** | **Unit** | **N** | **mean** | **SE** | **SD** | **median** | **min** | **max** | **N** | **mean** | **SE** | **SD** | **median** | **min** | **max** |  |
| Placebo | SCR | GammaGT | U/L | 12 | 16.7 | 3.2 | 10.9 | 14 | 9 | 50 |  |  |  |  |  |  |  |  |
|  |  | Glucose | mmol/L | 12 | 4.92 | 0.11 | 0.37 | 5.0 | 4.2 | 5.6 |  |  |  |  |  |  |  |  |
|  |  | LDH | U/L | 12 | 171.6 | 6.8 | 23.6 | 164 | 134 | 213 |  |  |  |  |  |  |  |  |
|  |  | Phosphate | mmol/L | 12 | 1.113 | 0.029 | 0.099 | 1.13 | 0.97 | 1.28 |  |  |  |  |  |  |  |  |
|  |  | Potassium | mmol/L | 12 | 4.35 | 0.11 | 0.37 | 4.3 | 3.9 | 5.0 |  |  |  |  |  |  |  |  |
|  |  | Sodium | mmol/L | 12 | 139.8 | 0.6 | 2.2 | 140 | 136 | 143 |  |  |  |  |  |  |  |  |
|  |  | TotProtein | g/L | 12 | 72.9 | 1.0 | 3.5 | 73 | 66 | 80 |  |  |  |  |  |  |  |  |
|  |  | Triglycerides | mmol/L | 12 | 1.043 | 0.176 | 0.608 | 0.82 | 0.60 | 2.61 |  |  |  |  |  |  |  |  |
|  |  | Urea | mmol/L | 12 | 4.24 | 0.25 | 0.86 | 4.3 | 3.1 | 5.8 |  |  |  |  |  |  |  |  |
|  |  | Serum uric acid | mmol/L | 12 | 0.303 | 0.023 | 0.081 | 0.32 | 0.15 | 0.40 |  |  |  |  |  |  |  |  |
|  | Day -1 | ALAT | U/L | 12 | 21.2 | 2.9 | 10.1 | 18 | 11 | 44 |  |  |  |  |  |  |  |  |
|  |  | Albumin | g/L | 12 | 47.8 | 0.4 | 1.5 | 48 | 46 | 51 |  |  |  |  |  |  |  |  |
|  |  | AlkPhos | U/L | 12 | 56.8 | 5.0 | 17.2 | 54 | 29 | 94 |  |  |  |  |  |  |  |  |
|  |  | ASAT | U/L | 12 | 19.8 | 1.5 | 5.2 | 19 | 13 | 29 |  |  |  |  |  |  |  |  |
|  |  | CRP | mg/L | 8 | 1.538 | 0.695 | 1.965 | 0.85 | 0.30 | 6.20 |  |  |  |  |  |  |  |  |
|  |  | Conjug Bilirubin | umol/L | 1 | 7.0 |  |  | 7 | 7 | 7 |  |  |  |  |  |  |  |  |
|  |  | Total Bilirubin | umol/L | 12 | 11.3 | 1.7 | 5.8 | 10 | 6 | 27 |  |  |  |  |  |  |  |  |
|  |  | Calcium | mmol/L | 12 | 2.319 | 0.021 | 0.072 | 2.33 | 2.20 | 2.41 |  |  |  |  |  |  |  |  |
|  |  | Creatinin | umol/L | 12 | 73.3 | 4.5 | 15.6 | 69 | 55 | 110 |  |  |  |  |  |  |  |  |
|  |  | GammaGT | U/L | 12 | 16.0 | 3.1 | 10.7 | 13 | 9 | 49 |  |  |  |  |  |  |  |  |
|  |  | Glucose | mmol/L | 12 | 4.72 | 0.11 | 0.39 | 4.7 | 4.2 | 5.5 |  |  |  |  |  |  |  |  |
|  |  | LDH | U/L | 12 | 160.3 | 4.8 | 16.7 | 158 | 137 | 185 |  |  |  |  |  |  |  |  |
|  |  | Phosphate | mmol/L | 12 | 1.069 | 0.036 | 0.124 | 1.08 | 0.84 | 1.24 |  |  |  |  |  |  |  |  |
|  |  | Potassium | mmol/L | 12 | 4.28 | 0.10 | 0.34 | 4.2 | 3.8 | 5.0 |  |  |  |  |  |  |  |  |
|  |  | Sodium | mmol/L | 12 | 139.9 | 0.2 | 0.7 | 140 | 139 | 141 |  |  |  |  |  |  |  |  |
|  |  | TotProtein | g/L | 12 | 68.5 | 0.8 | 2.9 | 69 | 64 | 72 |  |  |  |  |  |  |  |  |
|  |  | Triglycerides | mmol/L | 12 | 0.910 | 0.173 | 0.599 | 0.81 | 0.38 | 2.70 |  |  |  |  |  |  |  |  |
|  |  | Urea | mmol/L | 12 | 4.26 | 0.25 | 0.88 | 4.2 | 3.0 | 5.4 |  |  |  |  |  |  |  |  |
|  |  | Serum uric acid | mmol/L | 12 | 0.303 | 0.021 | 0.073 | 0.32 | 0.15 | 0.37 |  |  |  |  |  |  |  |  |
|  | Day 22 | ALAT | U/L | 12 | 21.1 | 2.6 | 8.9 | 20 | 11 | 46 | 12 | -0.1 | 1.7 | 6.0 | 1 | -17 | 8 |  |
|  |  | Albumin | g/L | 12 | 47.3 | 0.8 | 2.8 | 48 | 41 | 52 | 12 | -0.4 | 0.8 | 2.9 | -1 | -8 | 3 |  |
|  |  | AlkPhos | U/L | 12 | 59.3 | 4.8 | 16.6 | 58 | 31 | 88 | 12 | 2.5 | 1.5 | 5.3 | 1 | -6 | 12 |  |
|  |  | ASAT | U/L | 12 | 19.6 | 1.0 | 3.4 | 19 | 13 | 24 | 12 | -0.2 | 1.2 | 4.0 | 0 | -8 | 5 |  |
|  | | | | | | | | | | | | | | | | | |  |

|  | | | | | | | | | | | | | | | | | | |
| --- | --- | --- | --- | --- | --- | --- | --- | --- | --- | --- | --- | --- | --- | --- | --- | --- | --- | --- |
|  | | | | **Result** | | | | | | | **\| Change from baseline** | | | | | | |  |
| **Treatment** | **Protocol time** | **Label** | **Unit** | **N** | **mean** | **SE** | **SD** | **median** | **min** | **max** | **N** | **mean** | **SE** | **SD** | **median** | **min** | **max** |  |
| Placebo | Day 22 | CRP | mg/L | 12 | 1.033 | 0.350 | 1.212 | 0.40 | 0.30 | 4.30 | 8 | -0.138 | 0.390 | 1.102 | -0.10 | -1.90 | 1.90 |  |
|  |  | Conjug Bilirubin | umol/L | 2 | 6.5 | 0.5 | 0.7 | 7 | 6 | 7 | 1 | 0.0 |  |  | 0 | 0 | 0 |  |
|  |  | Total Bilirubin | umol/L | 12 | 13.1 | 1.8 | 6.2 | 12 | 5 | 28 | 12 | 1.8 | 1.4 | 4.9 | 1 | -4 | 11 |  |
|  |  | Calcium | mmol/L | 12 | 2.306 | 0.019 | 0.066 | 2.32 | 2.17 | 2.38 | 12 | -0.013 | 0.026 | 0.091 | -0.04 | -0.13 | 0.15 |  |
|  |  | Creatinin | umol/L | 12 | 75.2 | 4.2 | 14.6 | 73 | 53 | 110 | 12 | 1.8 | 1.3 | 4.3 | 0 | -2 | 11 |  |
|  |  | GammaGT | U/L | 12 | 14.1 | 2.4 | 8.3 | 11 | 9 | 40 | 12 | -1.9 | 0.8 | 2.9 | -2 | -9 | 3 |  |
|  |  | LDH | U/L | 12 | 160.4 | 2.5 | 8.5 | 161 | 142 | 173 | 12 | 0.1 | 3.7 | 12.7 | 0 | -16 | 28 |  |
|  |  | Phosphate | mmol/L | 12 | 1.025 | 0.044 | 0.153 | 1.01 | 0.71 | 1.33 | 12 | -0.044 | 0.043 | 0.149 | -0.01 | -0.33 | 0.16 |  |
|  |  | Potassium | mmol/L | 12 | 4.29 | 0.07 | 0.25 | 4.4 | 3.6 | 4.5 | 12 | 0.01 | 0.08 | 0.28 | 0.1 | -0.6 | 0.4 |  |
|  |  | Sodium | mmol/L | 12 | 139.9 | 0.4 | 1.4 | 140 | 138 | 142 | 12 | 0.0 | 0.3 | 1.2 | 0 | -2 | 2 |  |
|  |  | TotProtein | g/L | 12 | 68.5 | 1.2 | 4.3 | 69 | 61 | 76 | 12 | 0.0 | 1.3 | 4.3 | 1 | -11 | 5 |  |
|  |  | Triglycerides | mmol/L | 12 | 1.008 | 0.200 | 0.693 | 0.73 | 0.44 | 2.78 | 12 | 0.098 | 0.097 | 0.337 | -0.02 | -0.39 | 0.73 |  |
|  |  | Urea | mmol/L | 12 | 4.61 | 0.33 | 1.14 | 4.5 | 3.3 | 7.0 | 12 | 0.35 | 0.27 | 0.95 | 0.1 | -0.6 | 2.6 |  |
|  |  | Serum uric acid | mmol/L | 12 | 0.289 | 0.026 | 0.090 | 0.32 | 0.15 | 0.42 | 12 | -0.013 | 0.016 | 0.057 | 0.00 | -0.14 | 0.06 |  |
|  | Day 57 | ALAT | U/L | 12 | 22.3 | 2.7 | 9.2 | 20 | 12 | 43 | 12 | 1.2 | 2.0 | 6.8 | 2 | -12 | 13 |  |
|  |  | Albumin | g/L | 12 | 49.8 | 0.9 | 3.1 | 50 | 44 | 55 | 12 | 2.0 | 0.8 | 2.6 | 3 | -2 | 7 |  |
|  |  | AlkPhos | U/L | 12 | 63.1 | 5.2 | 18.2 | 60 | 34 | 98 | 12 | 6.3 | 2.3 | 8.1 | 5 | -7 | 27 |  |
|  |  | ASAT | U/L | 12 | 20.3 | 1.4 | 4.7 | 20 | 15 | 32 | 12 | 0.6 | 1.5 | 5.3 | 2 | -9 | 13 |  |
|  |  | CRP | mg/L | 11 | 2.618 | 1.112 | 3.687 | 1.10 | 0.30 | 11.90 | 8 | 1.950 | 1.393 | 3.940 | 0.55 | -0.70 | 11.50 |  |
|  |  | Conjug Bilirubin | umol/L | 1 | 5.0 |  |  | 5 | 5 | 5 | 1 | -2.0 |  |  | -2 | -2 | -2 |  |
|  |  | Total Bilirubin | umol/L | 12 | 10.0 | 1.3 | 4.6 | 9 | 3 | 21 | 12 | -1.3 | 1.1 | 3.9 | -3 | -6 | 6 |  |
|  |  | Calcium | mmol/L | 12 | 2.383 | 0.018 | 0.062 | 2.38 | 2.29 | 2.50 | 12 | 0.063 | 0.019 | 0.064 | 0.09 | -0.07 | 0.15 |  |
|  |  | Creatinin | umol/L | 12 | 73.6 | 3.8 | 13.0 | 74 | 57 | 100 | 12 | 0.3 | 2.2 | 7.6 | -3 | -10 | 19 |  |
|  |  | GammaGT | U/L | 12 | 15.9 | 2.4 | 8.3 | 14 | 10 | 41 | 12 | -0.1 | 0.9 | 3.1 | 0 | -8 | 4 |  |
|  |  | LDH | U/L | 12 | 172.1 | 6.2 | 21.5 | 170 | 125 | 216 | 12 | 11.8 | 5.3 | 18.2 | 13 | -19 | 39 |  |
|  |  | Phosphate | mmol/L | 12 | 1.034 | 0.041 | 0.141 | 1.05 | 0.80 | 1.21 | 12 | -0.035 | 0.031 | 0.106 | -0.03 | -0.24 | 0.13 |  |
|  |  | Potassium | mmol/L | 12 | 4.37 | 0.09 | 0.31 | 4.4 | 3.8 | 4.8 | 12 | 0.08 | 0.12 | 0.41 | 0.2 | -0.6 | 0.7 |  |
|  |  | Sodium | mmol/L | 12 | 138.3 | 1.1 | 3.8 | 139 | 127 | 142 | 12 | -1.6 | 1.1 | 3.8 | -1 | -13 | 2 |  |
|  |  | TotProtein | g/L | 12 | 72.3 | 1.0 | 3.3 | 73 | 66 | 77 | 12 | 3.8 | 1.0 | 3.5 | 4 | -2 | 11 |  |
|  |  | Triglycerides | mmol/L | 12 | 0.927 | 0.145 | 0.503 | 0.78 | 0.53 | 2.40 | 12 | 0.017 | 0.066 | 0.228 | 0.05 | -0.42 | 0.38 |  |
|  |  | Urea | mmol/L | 12 | 4.20 | 0.24 | 0.85 | 4.2 | 3.0 | 5.7 | 12 | -0.06 | 0.23 | 0.81 | -0.2 | -1.2 | 1.4 |  |
|  |  | Serum uric acid | mmol/L | 12 | 0.311 | 0.027 | 0.092 | 0.35 | 0.11 | 0.42 | 12 | 0.008 | 0.011 | 0.036 | 0.01 | -0.04 | 0.06 |  |
|  | Day 60 | ALAT | U/L | 12 | 21.3 | 2.7 | 9.3 | 19 | 13 | 46 | 12 | 0.2 | 2.3 | 8.0 | 1 | -20 | 11 |  |
|  | | | | | | | | | | | | | | | | | |  |

|  | | | | **Result** | | | | | | | **\| Change from baseline** | | | | | | |
| --- | --- | --- | --- | --- | --- | --- | --- | --- | --- | --- | --- | --- | --- | --- | --- | --- | --- |
| **Treatment** | **Protocol time** | **Label** | **Unit** | **N** | **mean** | **SE** | **SD** | **median** | **min** | **max** | **N** | **mean** | **SE** | **SD** | **median** | **min** | **max** |
| Placebo | Day 60 | Albumin | g/L | 12 | 48.2 | 0.8 | 2.7 | 48 | 44 | 55 | 12 | 0.4 | 0.5 | 1.6 | 1 | -3 | 4 |
|  |  | AlkPhos | U/L | 12 | 57.8 | 4.5 | 15.6 | 57 | 31 | 90 | 12 | 1.0 | 2.0 | 6.9 | 2 | -14 | 13 |
|  |  | ASAT | U/L | 12 | 20.0 | 1.2 | 4.1 | 20 | 13 | 26 | 12 | 0.3 | 1.1 | 3.7 | -1 | -7 | 7 |
|  |  | CRP | mg/L | 9 | 1.600 | 0.643 | 1.929 | 0.80 | 0.30 | 5.90 | 7 | 0.271 | 0.503 | 1.331 | -0.20 | -0.80 | 3.20 |
|  |  | Conjug Bilirubin | umol/L | 1 | 6.0 |  |  | 6 | 6 | 6 | 1 | -1.0 |  |  | -1 | -1 | -1 |
|  |  | Total Bilirubin | umol/L | 12 | 9.1 | 1.4 | 5.0 | 7 | 5 | 22 | 12 | -2.2 | 1.2 | 4.1 | -3 | -7 | 8 |
|  |  | Calcium | mmol/L | 12 | 2.323 | 0.021 | 0.072 | 2.32 | 2.22 | 2.45 | 12 | 0.003 | 0.021 | 0.072 | 0.02 | -0.15 | 0.11 |
|  |  | Creatinin | umol/L | 12 | 72.8 | 4.0 | 13.8 | 70 | 53 | 101 | 12 | -0.6 | 2.1 | 7.4 | -2 | -9 | 17 |
|  |  | GammaGT | U/L | 12 | 14.1 | 2.4 | 8.2 | 12 | 8 | 38 | 12 | -1.9 | 1.1 | 3.7 | -2 | -11 | 4 |
|  |  | LDH | U/L | 12 | 163.7 | 8.8 | 30.5 | 156 | 121 | 234 | 12 | 3.3 | 6.2 | 21.4 | 2 | -31 | 49 |
|  |  | Phosphate | mmol/L | 12 | 1.054 | 0.033 | 0.113 | 1.03 | 0.95 | 1.38 | 12 | -0.015 | 0.036 | 0.126 | -0.03 | -0.20 | 0.15 |
|  |  | Potassium | mmol/L | 12 | 4.50 | 0.07 | 0.24 | 4.5 | 4.0 | 4.9 | 12 | 0.22 | 0.12 | 0.43 | 0.4 | -1.0 | 0.6 |
|  |  | Sodium | mmol/L | 12 | 139.0 | 0.3 | 1.1 | 139 | 137 | 141 | 12 | -0.9 | 0.3 | 1.1 | -1 | -2 | 2 |
|  |  | TotProtein | g/L | 12 | 69.1 | 1.1 | 3.9 | 71 | 63 | 75 | 12 | 0.6 | 0.8 | 2.8 | 0 | -5 | 6 |
|  |  | Triglycerides | mmol/L | 12 | 0.918 | 0.159 | 0.552 | 0.72 | 0.40 | 2.30 | 12 | 0.008 | 0.115 | 0.398 | -0.06 | -0.52 | 0.74 |
|  |  | Urea | mmol/L | 12 | 4.03 | 0.30 | 1.05 | 4.0 | 2.3 | 5.5 | 12 | -0.23 | 0.32 | 1.12 | -0.1 | -1.7 | 2.5 |
|  |  | Serum uric acid | mmol/L | 12 | 0.299 | 0.025 | 0.086 | 0.32 | 0.13 | 0.40 | 12 | -0.003 | 0.013 | 0.046 | -0.01 | -0.06 | 0.11 |
|  | Day 60 | Glucose | mmol/L | 12 | 4.69 | 0.09 | 0.33 | 4.8 | 4.0 | 5.2 | 12 | -0.03 | 0.09 | 0.32 | -0.1 | -0.5 | 0.4 |
|  | FU | ALAT | U/L | 12 | 22.4 | 2.5 | 8.7 | 21 | 11 | 41 |  |  |  |  |  |  |  |
|  |  | Albumin | g/L | 12 | 48.7 | 1.0 | 3.4 | 49 | 43 | 53 |  |  |  |  |  |  |  |
|  |  | AlkPhos | U/L | 12 | 57.4 | 4.9 | 17.1 | 55 | 36 | 103 |  |  |  |  |  |  |  |
|  |  | ASAT | U/L | 12 | 21.3 | 1.6 | 5.4 | 20 | 14 | 33 |  |  |  |  |  |  |  |
|  |  | CRP | mg/L | 10 | 1.210 | 0.668 | 2.113 | 0.60 | 0.30 | 7.20 |  |  |  |  |  |  |  |
|  |  | Conjug Bilirubin | umol/L | 2 | 5.5 | 0.5 | 0.7 | 6 | 5 | 6 |  |  |  |  |  |  |  |
|  |  | Total Bilirubin | umol/L | 12 | 11.0 | 1.3 | 4.6 | 12 | 4 | 18 |  |  |  |  |  |  |  |
|  |  | Calcium | mmol/L | 12 | 2.366 | 0.020 | 0.068 | 2.37 | 2.23 | 2.51 |  |  |  |  |  |  |  |
|  |  | Creatinin | umol/L | 12 | 75.7 | 4.9 | 17.1 | 73 | 52 | 116 |  |  |  |  |  |  |  |
|  |  | GammaGT | U/L | 12 | 14.8 | 2.4 | 8.4 | 12 | 9 | 40 |  |  |  |  |  |  |  |
|  |  | LDH | U/L | 12 | 160.8 | 3.1 | 10.8 | 159 | 146 | 177 |  |  |  |  |  |  |  |
|  |  | Phosphate | mmol/L | 12 | 1.128 | 0.055 | 0.190 | 1.11 | 0.75 | 1.43 |  |  |  |  |  |  |  |
|  |  | Potassium | mmol/L | 12 | 4.46 | 0.10 | 0.34 | 4.6 | 3.9 | 5.0 |  |  |  |  |  |  |  |
|  |  | Sodium | mmol/L | 12 | 140.0 | 0.4 | 1.3 | 140 | 138 | 142 |  |  |  |  |  |  |  |
|  |  | TotProtein | g/L | 12 | 70.1 | 0.8 | 2.6 | 70 | 67 | 75 |  |  |  |  |  |  |  |
|  | | | | | | | | | | | | | | | | | |
| Placebo | FU | Triglycerides | mmol/L | 12 | 1.238 | 0.209 | 0.724 | 0.93 | 0.48 | 2.65 |  |  |  |  |  |  |  |
|  |  | Urea | mmol/L | 12 | 4.23 | 0.24 | 0.83 | 4.2 | 3.2 | 6.0 |  |  |  |  |  |  |  |
|  |  | Serum uric acid | mmol/L | 12 | 0.302 | 0.021 | 0.074 | 0.33 | 0.15 | 0.38 |  |  |  |  |  |  |  |

**Table S7.** **Summary of laboratory haematology results.** Haematology results are presented by treatment and protocol time, including change from baseline (baseline: Day -1). MCH: mean corpuscular haemoglobin, MCHC: mean corpuscular haemoglobin concentration, MCV: mean corpuscular volume, FU: follow-up visit, SD: standard deviation, SE: standard error, min: minimum, max: maximum, SCR: screening visit.

|  | | | | **Result** | | | | | | | **\| Change from baseline** | | | | | | |  |
| --- | --- | --- | --- | --- | --- | --- | --- | --- | --- | --- | --- | --- | --- | --- | --- | --- | --- | --- |
| **Treatment** | **Protocol time** | **Label** | **Unit** | **N** | **mean** | **SE** | **SD** | **median** | **min** | **max** | **N** | **mean** | **SE** | **SD** | **median** | **min** | **max** |  |
| EDP1815 EC1 | SCR | Basophils | 10E9/L | 13 | 0.047 | 0.006 | 0.022 | 0.04 | 0.03 | 0.11 |  |  |  |  |  |  |  |  |
|  |  | Eosinophils | 10E9/L | 13 | 0.191 | 0.055 | 0.198 | 0.12 | 0.03 | 0.76 |  |  |  |  |  |  |  |  |
|  |  | Erythrocytes | 10E12/L | 13 | 4.772 | 0.111 | 0.400 | 4.66 | 4.19 | 5.71 |  |  |  |  |  |  |  |  |
|  |  | Hematocrit | L/L | 13 | 0.4138 | 0.0075 | 0.0270 | 0.415 | 0.371 | 0.459 |  |  |  |  |  |  |  |  |
|  |  | Hemoglobin | mmol/L | 13 | 8.74 | 0.23 | 0.84 | 8.4 | 7.2 | 10.0 |  |  |  |  |  |  |  |  |
|  |  | Leucocytes | 10E9/L | 13 | 6.168 | 0.401 | 1.444 | 6.25 | 3.98 | 8.98 |  |  |  |  |  |  |  |  |
|  |  | Lymphocytes | 10E9/L | 13 | 1.875 | 0.103 | 0.370 | 1.80 | 1.44 | 2.55 |  |  |  |  |  |  |  |  |
|  |  | MCH | fmol | 13 | 1.835 | 0.039 | 0.142 | 1.85 | 1.51 | 2.05 |  |  |  |  |  |  |  |  |
|  |  | MCHC | mmol/L | 13 | 21.085 | 0.271 | 0.978 | 21.40 | 18.90 | 22.60 |  |  |  |  |  |  |  |  |
|  |  | MCV | fL | 13 | 86.9 | 1.2 | 4.3 | 88 | 80 | 92 |  |  |  |  |  |  |  |  |
|  |  | Monocytes | 10E9/L | 13 | 0.403 | 0.023 | 0.081 | 0.37 | 0.27 | 0.53 |  |  |  |  |  |  |  |  |
|  |  | Neutrophils | 10E9/L | 13 | 3.652 | 0.355 | 1.279 | 3.55 | 1.88 | 6.23 |  |  |  |  |  |  |  |  |
|  |  | Platelet count | 10E9/L | 13 | 239.1 | 11.9 | 42.9 | 248 | 182 | 322 |  |  |  |  |  |  |  |  |
|  | Day -1 | Basophils | 10E9/L | 13 | 0.047 | 0.006 | 0.021 | 0.04 | 0.03 | 0.08 |  |  |  |  |  |  |  |  |
|  |  | Eosinophils | 10E9/L | 13 | 0.182 | 0.035 | 0.127 | 0.15 | 0.04 | 0.48 |  |  |  |  |  |  |  |  |
|  |  | Erythrocytes | 10E12/L | 13 | 4.648 | 0.131 | 0.472 | 4.67 | 3.95 | 5.66 |  |  |  |  |  |  |  |  |
|  |  | Hematocrit | L/L | 13 | 0.4011 | 0.0107 | 0.0386 | 0.410 | 0.329 | 0.455 |  |  |  |  |  |  |  |  |
|  |  | Hemoglobin | mmol/L | 13 | 8.54 | 0.28 | 1.02 | 8.5 | 6.5 | 10.4 |  |  |  |  |  |  |  |  |
|  |  | Leucocytes | 10E9/L | 13 | 5.456 | 0.363 | 1.310 | 5.24 | 3.86 | 8.76 |  |  |  |  |  |  |  |  |
|  |  | Lymphocytes | 10E9/L | 13 | 1.796 | 0.082 | 0.295 | 1.83 | 1.22 | 2.48 |  |  |  |  |  |  |  |  |
|  |  | MCH | fmol | 13 | 1.839 | 0.038 | 0.136 | 1.85 | 1.54 | 2.05 |  |  |  |  |  |  |  |  |
|  |  | MCHC | mmol/L | 13 | 21.254 | 0.218 | 0.787 | 21.20 | 19.80 | 22.90 |  |  |  |  |  |  |  |  |
|  |  | MCV | fL | 13 | 86.5 | 1.2 | 4.3 | 87 | 78 | 91 |  |  |  |  |  |  |  |  |
|  |  | Monocytes | 10E9/L | 13 | 0.427 | 0.025 | 0.091 | 0.43 | 0.26 | 0.55 |  |  |  |  |  |  |  |  |
|  |  | Neutrophils | 10E9/L | 13 | 3.004 | 0.314 | 1.133 | 2.72 | 1.72 | 6.13 |  |  |  |  |  |  |  |  |
|  |  | Platelet count | 10E9/L | 13 | 223.5 | 10.9 | 39.4 | 224 | 161 | 317 |  |  |  |  |  |  |  |  |
|  | Day 22 | Basophils | 10E9/L | 12 | 0.038 | 0.003 | 0.012 | 0.04 | 0.02 | 0.06 | 12 | -0.010 | 0.005 | 0.016 | -0.01 | -0.04 | 0.01 |  |
|  |  | Eosinophils | 10E9/L | 12 | 0.183 | 0.039 | 0.133 | 0.14 | 0.04 | 0.49 | 12 | -0.008 | 0.031 | 0.106 | -0.01 | -0.23 | 0.14 |  |
|  |  | Erythrocytes | 10E12/L | 12 | 4.571 | 0.126 | 0.436 | 4.55 | 3.96 | 5.38 | 12 | -0.083 | 0.077 | 0.266 | -0.04 | -0.52 | 0.36 |  |
|  |  | Hematocrit | L/L | 12 | 0.3943 | 0.0096 | 0.0333 | 0.405 | 0.348 | 0.438 | 12 | -0.0073 | 0.0069 | 0.0238 | -0.004 | -0.056 | 0.027 |  |
|  |  | Hemoglobin | mmol/L | 12 | 8.42 | 0.25 | 0.86 | 8.6 | 7.0 | 9.8 | 12 | -0.17 | 0.14 | 0.48 | -0.2 | -1.1 | 0.5 |  |
|  |  | Leucocytes | 10E9/L | 12 | 4.966 | 0.316 | 1.095 | 4.72 | 3.63 | 7.74 | 12 | -0.371 | 0.234 | 0.810 | -0.30 | -1.70 | 0.85 |  |
|  |  | Lymphocytes | 10E9/L | 12 | 1.520 | 0.090 | 0.311 | 1.60 | 0.93 | 2.00 | 12 | -0.219 | 0.054 | 0.186 | -0.17 | -0.69 | -0.03 |  |
|  | | | | | | | | | | | | | | | | | |  |
| EDP1815 EC1 | Day 22 | MCH | fmol | 12 | 1.844 | 0.038 | 0.133 | 1.86 | 1.52 | 2.01 | 12 | -0.003 | 0.010 | 0.033 | -0.01 | -0.04 | 0.08 |  |
|  |  | MCHC | mmol/L | 12 | 21.325 | 0.179 | 0.621 | 21.40 | 19.70 | 22.40 | 12 | -0.008 | 0.108 | 0.375 | -0.20 | -0.50 | 0.90 |  |
|  |  | MCV | fL | 12 | 86.4 | 1.3 | 4.3 | 87 | 78 | 92 | 12 | -0.1 | 0.3 | 1.1 | 0 | -3 | 1 |  |
|  |  | Monocytes | 10E9/L | 12 | 0.363 | 0.021 | 0.074 | 0.37 | 0.23 | 0.49 | 12 | -0.056 | 0.018 | 0.061 | -0.05 | -0.13 | 0.06 |  |
|  |  | Neutrophils | 10E9/L | 12 | 2.861 | 0.306 | 1.059 | 2.60 | 1.58 | 5.21 | 12 | -0.078 | 0.233 | 0.807 | -0.09 | -1.10 | 1.33 |  |
|  |  | Platelet count | 10E9/L | 12 | 216.3 | 9.0 | 31.2 | 216 | 169 | 270 | 12 | -7.2 | 6.3 | 21.8 | -9 | -47 | 32 |  |
|  | Day 57 | Basophils | 10E9/L | 12 | 0.046 | 0.005 | 0.016 | 0.05 | 0.02 | 0.07 | 12 | -0.003 | 0.004 | 0.012 | 0.00 | -0.02 | 0.02 |  |
|  |  | Eosinophils | 10E9/L | 12 | 0.165 | 0.030 | 0.105 | 0.13 | 0.03 | 0.36 | 12 | -0.027 | 0.027 | 0.092 | -0.03 | -0.24 | 0.14 |  |
|  |  | Erythrocytes | 10E12/L | 12 | 4.715 | 0.132 | 0.458 | 4.64 | 3.96 | 5.59 | 12 | 0.061 | 0.077 | 0.266 | 0.07 | -0.29 | 0.37 |  |
|  |  | Hematocrit | L/L | 12 | 0.4097 | 0.0093 | 0.0323 | 0.411 | 0.361 | 0.454 | 12 | 0.0080 | 0.0070 | 0.0241 | 0.007 | -0.025 | 0.041 |  |
|  |  | Hemoglobin | mmol/L | 12 | 8.60 | 0.24 | 0.85 | 8.6 | 7.3 | 9.8 | 12 | 0.02 | 0.15 | 0.51 | 0.0 | -0.8 | 0.8 |  |
|  |  | Leucocytes | 10E9/L | 12 | 5.429 | 0.373 | 1.292 | 5.30 | 3.99 | 8.82 | 12 | 0.093 | 0.205 | 0.709 | 0.14 | -0.90 | 1.55 |  |
|  |  | Lymphocytes | 10E9/L | 12 | 1.746 | 0.133 | 0.460 | 1.78 | 0.88 | 2.66 | 12 | 0.007 | 0.088 | 0.305 | -0.07 | -0.34 | 0.71 |  |
|  |  | MCH | fmol | 12 | 1.828 | 0.036 | 0.124 | 1.82 | 1.59 | 2.01 | 12 | -0.019 | 0.010 | 0.034 | -0.02 | -0.07 | 0.05 |  |
|  |  | MCHC | mmol/L | 12 | 20.967 | 0.187 | 0.647 | 21.00 | 19.70 | 21.90 | 12 | -0.367 | 0.093 | 0.323 | -0.40 | -1.00 | 0.10 |  |
|  |  | MCV | fL | 12 | 87.2 | 1.1 | 3.9 | 88 | 80 | 92 | 12 | 0.7 | 0.4 | 1.3 | 1 | -2 | 3 |  |
|  |  | Monocytes | 10E9/L | 12 | 0.378 | 0.023 | 0.079 | 0.39 | 0.22 | 0.50 | 12 | -0.042 | 0.026 | 0.091 | -0.01 | -0.18 | 0.09 |  |
|  |  | Neutrophils | 10E9/L | 12 | 3.095 | 0.347 | 1.203 | 3.04 | 1.85 | 6.27 | 12 | 0.157 | 0.165 | 0.570 | 0.27 | -0.64 | 1.05 |  |
|  |  | Platelet count | 10E9/L | 12 | 229.5 | 12.2 | 42.2 | 216 | 172 | 319 | 12 | 6.0 | 6.2 | 21.6 | 4 | -22 | 48 |  |
|  | Day 60 | Basophils | 10E9/L | 12 | 0.046 | 0.005 | 0.019 | 0.05 | 0.02 | 0.07 | 12 | -0.003 | 0.004 | 0.013 | 0.00 | -0.03 | 0.02 |  |
|  |  | Eosinophils | 10E9/L | 12 | 0.206 | 0.047 | 0.164 | 0.13 | 0.03 | 0.58 | 12 | 0.014 | 0.039 | 0.136 | -0.01 | -0.16 | 0.40 |  |
|  |  | Erythrocytes | 10E12/L | 12 | 4.660 | 0.113 | 0.393 | 4.69 | 3.95 | 5.30 | 12 | 0.006 | 0.073 | 0.253 | -0.01 | -0.37 | 0.58 |  |
|  |  | Hematocrit | L/L | 12 | 0.4047 | 0.0086 | 0.0299 | 0.404 | 0.361 | 0.460 | 12 | 0.0030 | 0.0065 | 0.0226 | 0.003 | -0.030 | 0.056 |  |
|  |  | Hemoglobin | mmol/L | 12 | 8.48 | 0.23 | 0.80 | 8.6 | 7.5 | 9.9 | 12 | -0.10 | 0.13 | 0.44 | -0.2 | -0.6 | 1.0 |  |
|  |  | Leucocytes | 10E9/L | 12 | 5.261 | 0.396 | 1.372 | 4.99 | 3.41 | 8.49 | 12 | -0.076 | 0.316 | 1.096 | -0.12 | -2.26 | 1.90 |  |
|  |  | Lymphocytes | 10E9/L | 12 | 1.509 | 0.100 | 0.348 | 1.55 | 0.89 | 2.09 | 12 | -0.230 | 0.071 | 0.245 | -0.22 | -0.60 | 0.14 |  |
|  |  | MCH | fmol | 12 | 1.823 | 0.034 | 0.118 | 1.85 | 1.56 | 1.98 | 12 | -0.023 | 0.011 | 0.039 | -0.02 | -0.08 | 0.02 |  |
|  |  | MCHC | mmol/L | 12 | 20.950 | 0.169 | 0.584 | 21.10 | 19.50 | 21.60 | 12 | -0.383 | 0.153 | 0.529 | -0.30 | -1.40 | 0.30 |  |
|  |  | MCV | fL | 12 | 87.2 | 1.3 | 4.3 | 88 | 79 | 92 | 12 | 0.7 | 0.3 | 1.0 | 1 | -1 | 2 |  |
|  |  | Monocytes | 10E9/L | 12 | 0.421 | 0.038 | 0.131 | 0.41 | 0.23 | 0.63 | 12 | 0.002 | 0.021 | 0.072 | -0.03 | -0.08 | 0.14 |  |
|  |  | Neutrophils | 10E9/L | 12 | 3.122 | 0.359 | 1.243 | 2.89 | 1.61 | 5.86 | 12 | 0.183 | 0.334 | 1.156 | 0.41 | -2.26 | 1.96 |  |
|  |  | Platelet count | 10E9/L | 12 | 221.3 | 11.4 | 39.5 | 205 | 180 | 292 | 12 | -2.3 | 9.1 | 31.7 | -9 | -40 | 63 |  |
|  | FU | Basophils | 10E9/L | 13 | 0.045 | 0.005 | 0.018 | 0.04 | 0.02 | 0.08 |  |  |  |  |  |  |  |  |
|  | | | | | | | | | | | | | | | | | |  |
|  | | | | | | | | | | | | | | | | | | |

|  | | | | | | | | | | | | | | | | | | |
| --- | --- | --- | --- | --- | --- | --- | --- | --- | --- | --- | --- | --- | --- | --- | --- | --- | --- | --- |
|  | | | | **Result** | | | | | | | **\| Change from baseline** | | | | | | |  |
| **Treatment** | **Protocol time** | **Label** | **Unit** | **N** | **mean** | **SE** | **SD** | **median** | **min** | **max** | **N** | **mean** | **SE** | **SD** | **median** | **min** | **max** |  |
| EDP1815 EC1 | FU | Eosinophils | 10E9/L | 13 | 0.156 | 0.046 | 0.168 | 0.09 | 0.05 | 0.63 |  |  |  |  |  |  |  |  |
|  |  | Erythrocytes | 10E12/L | 13 | 4.709 | 0.131 | 0.471 | 4.59 | 4.10 | 5.72 |  |  |  |  |  |  |  |  |
|  |  | Hematocrit | L/L | 13 | 0.4122 | 0.0118 | 0.0426 | 0.405 | 0.366 | 0.527 |  |  |  |  |  |  |  |  |
|  |  | Hemoglobin | mmol/L | 13 | 8.64 | 0.29 | 1.06 | 8.3 | 6.9 | 11.2 |  |  |  |  |  |  |  |  |
|  |  | Leucocytes | 10E9/L | 13 | 5.615 | 0.373 | 1.344 | 5.34 | 4.15 | 9.15 |  |  |  |  |  |  |  |  |
|  |  | Lymphocytes | 10E9/L | 13 | 1.810 | 0.139 | 0.500 | 1.73 | 0.85 | 2.81 |  |  |  |  |  |  |  |  |
|  |  | MCH | fmol | 13 | 1.835 | 0.036 | 0.132 | 1.86 | 1.53 | 2.01 |  |  |  |  |  |  |  |  |
|  |  | MCHC | mmol/L | 13 | 20.938 | 0.229 | 0.825 | 21.20 | 18.90 | 22.20 |  |  |  |  |  |  |  |  |
|  |  | MCV | fL | 13 | 87.6 | 1.1 | 3.8 | 88 | 81 | 93 |  |  |  |  |  |  |  |  |
|  |  | Monocytes | 10E9/L | 13 | 0.388 | 0.033 | 0.118 | 0.36 | 0.20 | 0.58 |  |  |  |  |  |  |  |  |
|  |  | Neutrophils | 10E9/L | 13 | 3.215 | 0.305 | 1.098 | 2.89 | 1.99 | 5.83 |  |  |  |  |  |  |  |  |
|  |  | Platelet count | 10E9/L | 13 | 237.7 | 12.2 | 43.9 | 229 | 178 | 300 |  |  |  |  |  |  |  |  |
| EDP1815 EC2 | SCR | Basophils | 10E9/L | 13 | 0.045 | 0.005 | 0.017 | 0.05 | 0.02 | 0.07 |  |  |  |  |  |  |  |  |
|  |  | Eosinophils | 10E9/L | 13 | 0.195 | 0.054 | 0.193 | 0.14 | 0.03 | 0.78 |  |  |  |  |  |  |  |  |
|  |  | Erythrocytes | 10E12/L | 13 | 4.676 | 0.146 | 0.528 | 4.83 | 3.66 | 5.49 |  |  |  |  |  |  |  |  |
|  |  | Hematocrit | L/L | 13 | 0.4102 | 0.0097 | 0.0349 | 0.420 | 0.349 | 0.469 |  |  |  |  |  |  |  |  |
|  |  | Hemoglobin | mmol/L | 13 | 8.66 | 0.23 | 0.84 | 8.9 | 7.2 | 9.8 |  |  |  |  |  |  |  |  |
|  |  | Leucocytes | 10E9/L | 13 | 6.756 | 0.408 | 1.472 | 6.61 | 3.77 | 9.07 |  |  |  |  |  |  |  |  |
|  |  | Lymphocytes | 10E9/L | 13 | 1.919 | 0.125 | 0.449 | 1.81 | 1.03 | 2.83 |  |  |  |  |  |  |  |  |
|  |  | MCH | fmol | 13 | 1.856 | 0.027 | 0.097 | 1.84 | 1.74 | 2.06 |  |  |  |  |  |  |  |  |
|  |  | MCHC | mmol/L | 13 | 21.085 | 0.163 | 0.587 | 21.10 | 20.20 | 22.20 |  |  |  |  |  |  |  |  |
|  |  | MCV | fL | 13 | 87.9 | 1.2 | 4.3 | 86 | 83 | 96 |  |  |  |  |  |  |  |  |
|  |  | Monocytes | 10E9/L | 13 | 0.518 | 0.019 | 0.070 | 0.50 | 0.41 | 0.62 |  |  |  |  |  |  |  |  |
|  |  | Neutrophils | 10E9/L | 13 | 4.078 | 0.330 | 1.190 | 4.22 | 2.03 | 5.99 |  |  |  |  |  |  |  |  |
|  |  | Platelet count | 10E9/L | 13 | 256.6 | 11.1 | 39.9 | 241 | 205 | 341 |  |  |  |  |  |  |  |  |
|  | Day -1 | Basophils | 10E9/L | 13 | 0.038 | 0.004 | 0.016 | 0.04 | 0.02 | 0.07 |  |  |  |  |  |  |  |  |
|  |  | Eosinophils | 10E9/L | 13 | 0.154 | 0.031 | 0.111 | 0.13 | 0.05 | 0.48 |  |  |  |  |  |  |  |  |
|  |  | Erythrocytes | 10E12/L | 13 | 4.578 | 0.140 | 0.506 | 4.79 | 3.78 | 5.32 |  |  |  |  |  |  |  |  |
|  |  | Hematocrit | L/L | 13 | 0.4014 | 0.0071 | 0.0255 | 0.404 | 0.356 | 0.438 |  |  |  |  |  |  |  |  |
|  |  | Hemoglobin | mmol/L | 13 | 8.52 | 0.18 | 0.65 | 8.4 | 7.4 | 9.6 |  |  |  |  |  |  |  |  |
|  |  | Leucocytes | 10E9/L | 13 | 5.342 | 0.318 | 1.148 | 5.15 | 3.94 | 7.65 |  |  |  |  |  |  |  |  |
|  |  | Lymphocytes | 10E9/L | 13 | 1.644 | 0.116 | 0.419 | 1.56 | 1.12 | 2.81 |  |  |  |  |  |  |  |  |
|  |  | MCH | fmol | 13 | 1.868 | 0.029 | 0.106 | 1.85 | 1.73 | 2.08 |  |  |  |  |  |  |  |  |
|  | | | | | | | | | | | | | | | | | |  |

|  | | | | | | | | | | | | | | | | | | |
| --- | --- | --- | --- | --- | --- | --- | --- | --- | --- | --- | --- | --- | --- | --- | --- | --- | --- | --- |
|  | | | | **Result** | | | | | | | **\| Change from baseline** | | | | | | |  |
| **Treatment** | **Protocol time** | **Label** | **Unit** | **N** | **mean** | **SE** | **SD** | **median** | **min** | **max** | **N** | **mean** | **SE** | **SD** | **median** | **min** | **max** |  |
| EDP1815 EC2 | Day -1 | MCHC | mmol/L | 13 | 21.185 | 0.132 | 0.476 | 21.30 | 20.30 | 21.90 |  |  |  |  |  |  |  |  |
|  |  | MCV | fL | 13 | 88.2 | 1.4 | 5.1 | 86 | 82 | 98 |  |  |  |  |  |  |  |  |
|  |  | Monocytes | 10E9/L | 13 | 0.468 | 0.025 | 0.090 | 0.48 | 0.26 | 0.62 |  |  |  |  |  |  |  |  |
|  |  | Neutrophils | 10E9/L | 13 | 3.038 | 0.260 | 0.938 | 2.81 | 1.93 | 5.18 |  |  |  |  |  |  |  |  |
|  |  | Platelet count | 10E9/L | 13 | 239.5 | 10.3 | 37.0 | 241 | 171 | 289 |  |  |  |  |  |  |  |  |
|  | Day 22 | Basophils | 10E9/L | 12 | 0.038 | 0.005 | 0.016 | 0.04 | 0.01 | 0.07 | 12 | 0.000 | 0.003 | 0.010 | 0.00 | -0.01 | 0.02 |  |
|  |  | Eosinophils | 10E9/L | 12 | 0.167 | 0.047 | 0.162 | 0.13 | 0.04 | 0.64 | 12 | 0.013 | 0.018 | 0.061 | 0.01 | -0.10 | 0.16 |  |
|  |  | Erythrocytes | 10E12/L | 12 | 4.534 | 0.132 | 0.456 | 4.67 | 3.76 | 5.11 | 12 | -0.026 | 0.084 | 0.292 | -0.05 | -0.41 | 0.65 |  |
|  |  | Hematocrit | L/L | 12 | 0.3994 | 0.0075 | 0.0259 | 0.402 | 0.354 | 0.456 | 12 | -0.0026 | 0.0070 | 0.0242 | -0.007 | -0.032 | 0.043 |  |
|  |  | Hemoglobin | mmol/L | 12 | 8.45 | 0.14 | 0.50 | 8.6 | 7.5 | 9.4 | 12 | -0.08 | 0.16 | 0.56 | -0.1 | -0.9 | 1.2 |  |
|  |  | Leucocytes | 10E9/L | 12 | 5.248 | 0.383 | 1.326 | 4.73 | 3.68 | 7.83 | 12 | -0.201 | 0.126 | 0.438 | -0.19 | -0.96 | 0.51 |  |
|  |  | Lymphocytes | 10E9/L | 12 | 1.665 | 0.129 | 0.445 | 1.45 | 1.31 | 2.83 | 12 | 0.004 | 0.051 | 0.176 | 0.02 | -0.33 | 0.26 |  |
|  |  | MCH | fmol | 12 | 1.873 | 0.033 | 0.114 | 1.85 | 1.70 | 2.10 | 12 | -0.004 | 0.005 | 0.017 | 0.00 | -0.03 | 0.02 |  |
|  |  | MCHC | mmol/L | 12 | 21.158 | 0.154 | 0.533 | 21.20 | 20.30 | 22.20 | 12 | -0.017 | 0.153 | 0.529 | -0.10 | -1.00 | 0.80 |  |
|  |  | MCV | fL | 12 | 88.4 | 1.4 | 5.0 | 88 | 83 | 98 | 12 | -0.3 | 0.5 | 1.9 | 0 | -4 | 3 |  |
|  |  | Monocytes | 10E9/L | 12 | 0.437 | 0.025 | 0.087 | 0.43 | 0.26 | 0.57 | 12 | -0.049 | 0.025 | 0.087 | -0.09 | -0.19 | 0.09 |  |
|  |  | Neutrophils | 10E9/L | 12 | 2.942 | 0.285 | 0.988 | 2.77 | 1.78 | 5.28 | 12 | -0.168 | 0.085 | 0.295 | -0.20 | -0.50 | 0.41 |  |
|  |  | Platelet count | 10E9/L | 12 | 239.5 | 12.4 | 42.9 | 240 | 168 | 304 | 12 | 0.1 | 5.6 | 19.4 | -2 | -40 | 38 |  |
|  | Day 57 | Basophils | 10E9/L | 12 | 0.043 | 0.007 | 0.023 | 0.04 | 0.02 | 0.10 | 12 | 0.006 | 0.004 | 0.014 | 0.00 | -0.02 | 0.03 |  |
|  |  | Eosinophils | 10E9/L | 12 | 0.180 | 0.031 | 0.109 | 0.14 | 0.09 | 0.44 | 12 | 0.026 | 0.022 | 0.075 | 0.03 | -0.07 | 0.18 |  |
|  |  | Erythrocytes | 10E12/L | 12 | 4.538 | 0.145 | 0.501 | 4.62 | 3.63 | 5.31 | 12 | -0.022 | 0.072 | 0.248 | -0.14 | -0.29 | 0.46 |  |
|  |  | Hematocrit | L/L | 12 | 0.4034 | 0.0094 | 0.0326 | 0.396 | 0.346 | 0.469 | 12 | 0.0014 | 0.0066 | 0.0227 | -0.007 | -0.025 | 0.053 |  |
|  |  | Hemoglobin | mmol/L | 12 | 8.40 | 0.21 | 0.72 | 8.5 | 7.2 | 9.8 | 12 | -0.13 | 0.13 | 0.46 | -0.4 | -0.6 | 0.8 |  |
|  |  | Leucocytes | 10E9/L | 12 | 5.159 | 0.287 | 0.994 | 4.91 | 4.19 | 7.37 | 12 | -0.289 | 0.148 | 0.511 | -0.25 | -1.47 | 0.32 |  |
|  |  | Lymphocytes | 10E9/L | 12 | 1.648 | 0.126 | 0.435 | 1.64 | 0.91 | 2.69 | 12 | -0.013 | 0.051 | 0.177 | 0.00 | -0.36 | 0.25 |  |
|  |  | MCH | fmol | 12 | 1.858 | 0.028 | 0.098 | 1.84 | 1.70 | 2.00 | 12 | -0.020 | 0.011 | 0.037 | -0.02 | -0.08 | 0.06 |  |
|  |  | MCHC | mmol/L | 12 | 20.817 | 0.127 | 0.439 | 20.80 | 19.90 | 21.40 | 12 | -0.358 | 0.104 | 0.360 | -0.40 | -1.10 | 0.30 |  |
|  |  | MCV | fL | 12 | 89.2 | 1.2 | 4.2 | 89 | 83 | 96 | 12 | 0.4 | 0.6 | 2.2 | 0 | -3 | 4 |  |
|  |  | Monocytes | 10E9/L | 12 | 0.418 | 0.023 | 0.078 | 0.42 | 0.30 | 0.53 | 12 | -0.068 | 0.025 | 0.087 | -0.06 | -0.20 | 0.05 |  |
|  |  | Neutrophils | 10E9/L | 12 | 2.871 | 0.241 | 0.836 | 2.60 | 1.97 | 4.90 | 12 | -0.239 | 0.149 | 0.517 | -0.13 | -1.22 | 0.70 |  |
|  |  | Platelet count | 10E9/L | 12 | 238.8 | 11.1 | 38.6 | 232 | 179 | 291 | 12 | -0.6 | 4.0 | 14.0 | 2 | -30 | 21 |  |
|  | Day 60 | Basophils | 10E9/L | 11 | 0.039 | 0.007 | 0.024 | 0.04 | 0.01 | 0.10 | 11 | 0.002 | 0.004 | 0.014 | 0.00 | -0.01 | 0.03 |  |
|  |  | Eosinophils | 10E9/L | 11 | 0.187 | 0.029 | 0.096 | 0.19 | 0.06 | 0.36 | 11 | 0.025 | 0.028 | 0.093 | 0.04 | -0.12 | 0.12 |  |
|  | | | | | | | | | | | | | | | | | |  |

|  | | | | | | | | | | | | | | | | | | |
| --- | --- | --- | --- | --- | --- | --- | --- | --- | --- | --- | --- | --- | --- | --- | --- | --- | --- | --- |
|  | | | | **Result** | | | | | | | **\| Change from baseline** | | | | | | |  |
| **Treatment** | **Protocol time** | **Label** | **Unit** | **N** | **mean** | **SE** | **SD** | **median** | **min** | **max** | **N** | **mean** | **SE** | **SD** | **median** | **min** | **max** |  |
| EDP1815 EC2 | Day 60 | Erythrocytes | 10E12/L | 11 | 4.601 | 0.158 | 0.523 | 4.67 | 3.72 | 5.25 | 11 | -0.005 | 0.057 | 0.191 | -0.04 | -0.29 | 0.30 |  |
|  |  | Hematocrit | L/L | 11 | 0.4064 | 0.0092 | 0.0307 | 0.409 | 0.350 | 0.451 | 11 | 0.0009 | 0.0052 | 0.0173 | -0.001 | -0.023 | 0.035 |  |
|  |  | Hemoglobin | mmol/L | 11 | 8.62 | 0.19 | 0.63 | 8.5 | 7.4 | 9.8 | 11 | -0.01 | 0.12 | 0.41 | -0.1 | -0.5 | 0.8 |  |
|  |  | Leucocytes | 10E9/L | 11 | 4.752 | 0.404 | 1.340 | 4.71 | 2.87 | 6.76 | 11 | -0.496 | 0.273 | 0.906 | -0.25 | -2.19 | 0.54 |  |
|  |  | Lymphocytes | 10E9/L | 11 | 1.652 | 0.125 | 0.416 | 1.68 | 1.04 | 2.46 | 11 | 0.013 | 0.054 | 0.180 | 0.01 | -0.35 | 0.28 |  |
|  |  | MCH | fmol | 11 | 1.885 | 0.037 | 0.124 | 1.87 | 1.70 | 2.12 | 11 | 0.002 | 0.008 | 0.028 | 0.00 | -0.03 | 0.04 |  |
|  |  | MCHC | mmol/L | 11 | 21.209 | 0.170 | 0.563 | 21.30 | 20.30 | 22.10 | 11 | -0.045 | 0.128 | 0.425 | -0.10 | -0.60 | 0.80 |  |
|  |  | MCV | fL | 11 | 88.9 | 1.3 | 4.4 | 88 | 83 | 96 | 11 | 0.3 | 0.4 | 1.5 | 0 | -2 | 3 |  |
|  |  | Monocytes | 10E9/L | 11 | 0.435 | 0.026 | 0.085 | 0.42 | 0.33 | 0.62 | 11 | -0.053 | 0.025 | 0.081 | -0.07 | -0.15 | 0.06 |  |
|  |  | Neutrophils | 10E9/L | 11 | 2.438 | 0.262 | 0.870 | 2.62 | 1.19 | 3.48 | 11 | -0.484 | 0.243 | 0.805 | -0.27 | -1.96 | 0.96 |  |
|  |  | Platelet count | 10E9/L | 11 | 240.6 | 12.0 | 39.7 | 234 | 181 | 314 | 11 | 3.6 | 5.7 | 18.9 | 12 | -28 | 26 |  |
|  | FU | Basophils | 10E9/L | 13 | 0.045 | 0.004 | 0.014 | 0.04 | 0.02 | 0.07 |  |  |  |  |  |  |  |  |
|  |  | Eosinophils | 10E9/L | 13 | 0.190 | 0.044 | 0.158 | 0.18 | 0.06 | 0.68 |  |  |  |  |  |  |  |  |
|  |  | Erythrocytes | 10E12/L | 13 | 4.588 | 0.152 | 0.548 | 4.71 | 3.82 | 5.43 |  |  |  |  |  |  |  |  |
|  |  | Hematocrit | L/L | 13 | 0.4053 | 0.0099 | 0.0357 | 0.414 | 0.338 | 0.461 |  |  |  |  |  |  |  |  |
|  |  | Hemoglobin | mmol/L | 13 | 8.52 | 0.20 | 0.74 | 8.7 | 7.2 | 10.0 |  |  |  |  |  |  |  |  |
|  |  | Leucocytes | 10E9/L | 13 | 6.733 | 0.616 | 2.220 | 6.26 | 3.81 | 11.21 |  |  |  |  |  |  |  |  |
|  |  | Lymphocytes | 10E9/L | 13 | 1.802 | 0.125 | 0.451 | 1.82 | 1.21 | 2.79 |  |  |  |  |  |  |  |  |
|  |  | MCH | fmol | 13 | 1.865 | 0.033 | 0.119 | 1.86 | 1.67 | 2.10 |  |  |  |  |  |  |  |  |
|  |  | MCHC | mmol/L | 13 | 21.038 | 0.152 | 0.547 | 21.00 | 20.10 | 22.10 |  |  |  |  |  |  |  |  |
|  |  | MCV | fL | 13 | 88.8 | 1.2 | 4.4 | 88 | 83 | 96 |  |  |  |  |  |  |  |  |
|  |  | Monocytes | 10E9/L | 13 | 0.558 | 0.046 | 0.166 | 0.55 | 0.33 | 0.88 |  |  |  |  |  |  |  |  |
|  |  | Neutrophils | 10E9/L | 13 | 4.138 | 0.529 | 1.908 | 3.32 | 1.82 | 8.36 |  |  |  |  |  |  |  |  |
|  |  | Platelet count | 10E9/L | 13 | 258.1 | 13.1 | 47.4 | 248 | 180 | 338 |  |  |  |  |  |  |  |  |
| Placebo | SCR | Basophils | 10E9/L | 12 | 0.048 | 0.004 | 0.014 | 0.05 | 0.03 | 0.07 |  |  |  |  |  |  |  |  |
|  |  | Eosinophils | 10E9/L | 12 | 0.204 | 0.036 | 0.125 | 0.19 | 0.05 | 0.44 |  |  |  |  |  |  |  |  |
|  |  | Erythrocytes | 10E12/L | 12 | 4.844 | 0.127 | 0.438 | 4.82 | 4.12 | 5.73 |  |  |  |  |  |  |  |  |
|  |  | Hematocrit | L/L | 12 | 0.4269 | 0.0107 | 0.0372 | 0.422 | 0.374 | 0.488 |  |  |  |  |  |  |  |  |
|  |  | Hemoglobin | mmol/L | 12 | 9.06 | 0.23 | 0.79 | 9.0 | 7.9 | 10.5 |  |  |  |  |  |  |  |  |
|  |  | Leucocytes | 10E9/L | 12 | 6.723 | 0.433 | 1.501 | 6.44 | 4.87 | 10.05 |  |  |  |  |  |  |  |  |
|  |  | Lymphocytes | 10E9/L | 12 | 1.997 | 0.146 | 0.507 | 1.95 | 1.43 | 3.35 |  |  |  |  |  |  |  |  |
|  |  | MCH | fmol | 12 | 1.872 | 0.021 | 0.074 | 1.87 | 1.76 | 2.00 |  |  |  |  |  |  |  |  |
|  |  | MCHC | mmol/L | 12 | 21.225 | 0.162 | 0.561 | 21.20 | 20.50 | 22.40 |  |  |  |  |  |  |  |  |
|  | | | | | | | | | | | | | | | | | |  |

|  | | | | | | | | | | | | | | | | | | | |
| --- | --- | --- | --- | --- | --- | --- | --- | --- | --- | --- | --- | --- | --- | --- | --- | --- | --- | --- | --- |
|  | | | | **Result** | | | | | | | **\| Change from baseline** | | | | | | |  |  |
| **Treatment** | **Protocol time** | **Label** | **Unit** | **N** | **mean** | **SE** | **SD** | **median** | **min** | **max** | **N** | **mean** | **SE** | **SD** | **median** | **min** | **max** |  |  |
| Placebo | SCR | MCV | fL | 12 | 88.1 | 0.8 | 2.6 | 88 | 85 | 94 |  |  |  |  |  |  |  |  |  |
|  |  | Monocytes | 10E9/L | 12 | 0.547 | 0.030 | 0.104 | 0.54 | 0.34 | 0.76 |  |  |  |  |  |  |  |  |  |
|  |  | Neutrophils | 10E9/L | 12 | 3.928 | 0.420 | 1.453 | 3.72 | 2.23 | 7.12 |  |  |  |  |  |  |  |  |  |
|  |  | Platelet count | 10E9/L | 12 | 275.3 | 13.8 | 47.7 | 283 | 183 | 345 |  |  |  |  |  |  |  |  |  |
|  | Day -1 | Basophils | 10E9/L | 12 | 0.038 | 0.004 | 0.015 | 0.04 | 0.01 | 0.06 |  |  |  |  |  |  |  |  |  |
|  |  | Eosinophils | 10E9/L | 12 | 0.200 | 0.047 | 0.164 | 0.16 | 0.01 | 0.50 |  |  |  |  |  |  |  |  |  |
|  |  | Erythrocytes | 10E12/L | 12 | 4.683 | 0.125 | 0.433 | 4.56 | 4.04 | 5.39 |  |  |  |  |  |  |  |  |  |
|  |  | Hematocrit | L/L | 12 | 0.4133 | 0.0087 | 0.0302 | 0.410 | 0.362 | 0.462 |  |  |  |  |  |  |  |  |  |
|  |  | Hemoglobin | mmol/L | 12 | 8.80 | 0.21 | 0.71 | 8.9 | 7.5 | 10.0 |  |  |  |  |  |  |  |  |  |
|  |  | Leucocytes | 10E9/L | 12 | 5.552 | 0.499 | 1.729 | 4.94 | 3.90 | 9.07 |  |  |  |  |  |  |  |  |  |
|  |  | Lymphocytes | 10E9/L | 12 | 1.835 | 0.149 | 0.517 | 1.67 | 1.19 | 2.87 |  |  |  |  |  |  |  |  |  |
|  |  | MCH | fmol | 12 | 1.883 | 0.020 | 0.071 | 1.88 | 1.77 | 2.00 |  |  |  |  |  |  |  |  |  |
|  |  | MCHC | mmol/L | 12 | 21.275 | 0.171 | 0.593 | 21.40 | 20.30 | 22.30 |  |  |  |  |  |  |  |  |  |
|  |  | MCV | fL | 12 | 88.5 | 1.0 | 3.3 | 89 | 84 | 94 |  |  |  |  |  |  |  |  |  |
|  |  | Monocytes | 10E9/L | 12 | 0.500 | 0.033 | 0.113 | 0.47 | 0.38 | 0.80 |  |  |  |  |  |  |  |  |  |
|  |  | Neutrophils | 10E9/L | 12 | 2.979 | 0.384 | 1.329 | 2.45 | 1.83 | 5.70 |  |  |  |  |  |  |  |  |  |
|  |  | Platelet count | 10E9/L | 12 | 253.1 | 14.2 | 49.1 | 248 | 163 | 329 |  |  |  |  |  |  |  |  |  |
|  | Day 22 | Basophils | 10E9/L | 12 | 0.040 | 0.006 | 0.020 | 0.04 | 0.02 | 0.08 | 12 | 0.003 | 0.004 | 0.012 | 0.01 | -0.02 | 0.02 |  |  |
|  |  | Eosinophils | 10E9/L | 12 | 0.175 | 0.031 | 0.107 | 0.16 | 0.01 | 0.35 | 12 | -0.025 | 0.034 | 0.117 | 0.00 | -0.25 | 0.21 |  |  |
|  |  | Erythrocytes | 10E12/L | 12 | 4.699 | 0.113 | 0.392 | 4.67 | 3.81 | 5.28 | 12 | 0.016 | 0.061 | 0.211 | 0.05 | -0.32 | 0.35 |  |  |
|  |  | Hematocrit | L/L | 12 | 0.4131 | 0.0079 | 0.0273 | 0.417 | 0.358 | 0.460 | 12 | -0.0002 | 0.0052 | 0.0181 | 0.006 | -0.023 | 0.029 |  |  |
|  |  | Hemoglobin | mmol/L | 12 | 8.80 | 0.19 | 0.65 | 9.0 | 7.4 | 9.6 | 12 | 0.00 | 0.11 | 0.37 | 0.1 | -0.5 | 0.5 |  |  |
|  |  | Leucocytes | 10E9/L | 12 | 5.288 | 0.362 | 1.253 | 4.88 | 3.67 | 7.95 | 12 | -0.263 | 0.266 | 0.921 | -0.19 | -2.38 | 1.58 |  |  |
|  |  | Lymphocytes | 10E9/L | 12 | 1.697 | 0.107 | 0.369 | 1.66 | 1.07 | 2.17 | 12 | -0.138 | 0.096 | 0.334 | -0.03 | -0.84 | 0.36 |  |  |
|  |  | MCH | fmol | 12 | 1.875 | 0.019 | 0.067 | 1.87 | 1.78 | 1.98 | 12 | -0.008 | 0.005 | 0.018 | -0.01 | -0.04 | 0.01 |  |  |
|  |  | MCHC | mmol/L | 12 | 21.283 | 0.145 | 0.501 | 21.30 | 20.40 | 22.20 | 12 | 0.008 | 0.119 | 0.412 | -0.10 | -0.40 | 1.10 |  |  |
|  |  | MCV | fL | 12 | 88.1 | 0.9 | 3.2 | 89 | 83 | 94 | 12 | -0.4 | 0.5 | 1.7 | 0 | -4 | 1 |  |  |
|  |  | Monocytes | 10E9/L | 12 | 0.458 | 0.026 | 0.089 | 0.44 | 0.34 | 0.69 | 12 | -0.042 | 0.035 | 0.121 | -0.05 | -0.29 | 0.22 |  |  |
|  |  | Neutrophils | 10E9/L | 12 | 2.918 | 0.288 | 0.998 | 2.53 | 1.98 | 5.02 | 12 | -0.061 | 0.198 | 0.686 | -0.07 | -1.23 | 1.63 |  |  |
|  |  | Platelet count | 10E9/L | 12 | 252.2 | 12.4 | 42.9 | 248 | 171 | 321 | 12 | -0.9 | 8.1 | 28.1 | 3 | -54 | 49 |  |  |
|  | Day 57 | Basophils | 10E9/L | 12 | 0.042 | 0.005 | 0.019 | 0.04 | 0.02 | 0.07 | 12 | 0.004 | 0.004 | 0.015 | 0.01 | -0.02 | 0.03 |  |  |
|  |  | Eosinophils | 10E9/L | 12 | 0.229 | 0.049 | 0.171 | 0.21 | 0.00 | 0.63 | 12 | 0.029 | 0.021 | 0.072 | 0.01 | -0.06 | 0.15 |  |  |
|  |  | Erythrocytes | 10E12/L | 12 | 4.766 | 0.113 | 0.390 | 4.75 | 3.98 | 5.51 | 12 | 0.083 | 0.044 | 0.152 | 0.10 | -0.16 | 0.33 |  |  |
|  | | | | | | | | | | | | | | | | | |  |  |
|  | | | | **Result** | | | | | | | **\| Change from baseline** | | | | | | |  |  |
| **Treatment** | **Protocol time** | **Label** | **Unit** | **N** | **mean** | **SE** | **SD** | **median** | **min** | **max** | **N** | **mean** | **SE** | **SD** | **median** | **min** | **max** |  |  |
| Placebo | Day 57 | Hematocrit | L/L | 12 | 0.4233 | 0.0075 | 0.0259 | 0.430 | 0.373 | 0.460 | 12 | 0.0101 | 0.0033 | 0.0115 | 0.010 | -0.008 | 0.031 |  |  |
|  |  | Hemoglobin | mmol/L | 12 | 8.90 | 0.19 | 0.67 | 9.1 | 7.8 | 10.2 | 12 | 0.10 | 0.09 | 0.30 | 0.2 | -0.4 | 0.6 |  |  |
|  |  | Leucocytes | 10E9/L | 12 | 5.831 | 0.505 | 1.749 | 5.54 | 3.47 | 8.87 | 12 | 0.279 | 0.349 | 1.208 | 0.21 | -1.56 | 3.05 |  |  |
|  |  | Lymphocytes | 10E9/L | 12 | 1.837 | 0.139 | 0.483 | 1.71 | 1.26 | 2.99 | 12 | 0.002 | 0.073 | 0.252 | 0.10 | -0.62 | 0.29 |  |  |
|  |  | MCH | fmol | 12 | 1.869 | 0.021 | 0.074 | 1.87 | 1.72 | 1.96 | 12 | -0.013 | 0.012 | 0.042 | -0.01 | -0.08 | 0.05 |  |  |
|  |  | MCHC | mmol/L | 12 | 21.000 | 0.173 | 0.598 | 21.10 | 19.80 | 22.20 | 12 | -0.275 | 0.179 | 0.621 | -0.20 | -1.60 | 0.60 |  |  |
|  |  | MCV | fL | 12 | 89.1 | 0.9 | 3.1 | 90 | 84 | 94 | 12 | 0.6 | 0.5 | 1.6 | 1 | -2 | 3 |  |  |
|  |  | Monocytes | 10E9/L | 12 | 0.516 | 0.034 | 0.119 | 0.54 | 0.30 | 0.73 | 12 | 0.016 | 0.048 | 0.167 | 0.04 | -0.24 | 0.26 |  |  |
|  |  | Neutrophils | 10E9/L | 12 | 3.208 | 0.395 | 1.368 | 2.90 | 1.48 | 6.15 | 12 | 0.228 | 0.353 | 1.224 | 0.01 | -1.15 | 3.44 |  |  |
|  |  | Platelet count | 10E9/L | 12 | 258.3 | 15.5 | 53.8 | 262 | 157 | 335 | 12 | 5.3 | 8.2 | 28.5 | 8 | -46 | 46 |  |  |
|  | Day 60 | Basophils | 10E9/L | 12 | 0.040 | 0.006 | 0.021 | 0.04 | 0.02 | 0.09 | 12 | 0.003 | 0.005 | 0.017 | 0.00 | -0.02 | 0.04 |  |  |
|  |  | Eosinophils | 10E9/L | 12 | 0.203 | 0.043 | 0.148 | 0.19 | 0.04 | 0.51 | 12 | 0.003 | 0.012 | 0.043 | 0.00 | -0.06 | 0.08 |  |  |
|  |  | Erythrocytes | 10E12/L | 12 | 4.738 | 0.106 | 0.366 | 4.65 | 4.26 | 5.54 | 12 | 0.055 | 0.064 | 0.221 | 0.09 | -0.29 | 0.56 |  |  |
|  |  | Hematocrit | L/L | 12 | 0.4201 | 0.0070 | 0.0241 | 0.414 | 0.393 | 0.467 | 12 | 0.0068 | 0.0050 | 0.0173 | 0.005 | -0.017 | 0.049 |  |  |
|  |  | Hemoglobin | mmol/L | 12 | 8.85 | 0.18 | 0.64 | 8.9 | 8.1 | 10.2 | 12 | 0.05 | 0.11 | 0.37 | 0.1 | -0.8 | 0.7 |  |  |
|  |  | Leucocytes | 10E9/L | 12 | 5.413 | 0.396 | 1.371 | 5.31 | 3.49 | 7.74 | 12 | -0.139 | 0.395 | 1.369 | -0.17 | -3.08 | 1.97 |  |  |
|  |  | Lymphocytes | 10E9/L | 12 | 1.715 | 0.117 | 0.404 | 1.75 | 0.98 | 2.27 | 12 | -0.120 | 0.075 | 0.259 | -0.09 | -0.60 | 0.28 |  |  |
|  |  | MCH | fmol | 12 | 1.870 | 0.021 | 0.074 | 1.87 | 1.74 | 1.96 | 12 | -0.013 | 0.012 | 0.040 | -0.02 | -0.07 | 0.07 |  |  |
|  |  | MCHC | mmol/L | 12 | 21.058 | 0.179 | 0.619 | 21.20 | 20.00 | 21.80 | 12 | -0.217 | 0.126 | 0.437 | -0.20 | -1.00 | 0.30 |  |  |
|  |  | MCV | fL | 12 | 88.8 | 0.8 | 2.9 | 89 | 84 | 94 | 12 | 0.3 | 0.4 | 1.4 | 0 | -2 | 3 |  |  |
|  |  | Monocytes | 10E9/L | 12 | 0.475 | 0.025 | 0.087 | 0.46 | 0.33 | 0.63 | 12 | -0.025 | 0.031 | 0.108 | -0.03 | -0.17 | 0.11 |  |  |
|  |  | Neutrophils | 10E9/L | 12 | 2.979 | 0.281 | 0.972 | 2.75 | 1.98 | 4.81 | 12 | 0.000 | 0.335 | 1.159 | 0.05 | -2.44 | 2.10 |  |  |
|  |  | Platelet count | 10E9/L | 12 | 255.9 | 14.3 | 49.7 | 237 | 199 | 356 | 12 | 2.8 | 10.2 | 35.4 | 1 | -71 | 65 |  |  |
|  | FU | Basophils | 10E9/L | 12 | 0.047 | 0.004 | 0.015 | 0.04 | 0.03 | 0.09 |  |  |  |  |  |  |  |  |  |
|  |  | Eosinophils | 10E9/L | 12 | 0.231 | 0.050 | 0.173 | 0.18 | 0.04 | 0.70 |  |  |  |  |  |  |  |  |  |
|  |  | Erythrocytes | 10E12/L | 12 | 4.725 | 0.124 | 0.429 | 4.72 | 3.95 | 5.38 |  |  |  |  |  |  |  |  |  |
|  |  | Hematocrit | L/L | 12 | 0.4203 | 0.0089 | 0.0307 | 0.424 | 0.356 | 0.458 |  |  |  |  |  |  |  |  |  |
|  |  | Hemoglobin | mmol/L | 12 | 8.80 | 0.23 | 0.80 | 8.9 | 7.2 | 9.9 |  |  |  |  |  |  |  |  |  |
|  |  | Leucocytes | 10E9/L | 12 | 6.350 | 0.608 | 2.106 | 5.80 | 3.86 | 11.40 |  |  |  |  |  |  |  |  |  |
|  |  | Lymphocytes | 10E9/L | 12 | 1.921 | 0.198 | 0.687 | 1.80 | 0.92 | 3.61 |  |  |  |  |  |  |  |  |  |
|  |  | MCH | fmol | 12 | 1.863 | 0.024 | 0.082 | 1.88 | 1.71 | 2.00 |  |  |  |  |  |  |  |  |  |
|  |  | MCHC | mmol/L | 12 | 20.917 | 0.178 | 0.616 | 20.90 | 20.00 | 21.90 |  |  |  |  |  |  |  |  |  |
|  |  | MCV | fL | 12 | 89.0 | 0.9 | 3.1 | 90 | 85 | 94 |  |  |  |  |  |  |  |  |  |
|  | | | | | | | | | | | | | | | | | |  |  |
|  | | | | **Result** | | | | | | | **\| Change from baseline** | | | | | | |  |  |
| **Treatment** | **Protocol time** | **Label** | **Unit** | **N** | **mean** | **SE** | **SD** | **median** | **min** | **max** | **N** | **mean** | **SE** | **SD** | **median** | **min** | **max** |  |  |
| Placebo | FU | Monocytes | 10E9/L | 12 | 0.555 | 0.037 | 0.127 | 0.55 | 0.39 | 0.79 |  |  |  |  |  |  |  |  |  |
|  |  | Neutrophils | 10E9/L | 12 | 3.597 | 0.460 | 1.594 | 3.20 | 1.90 | 7.03 |  |  |  |  |  |  |  |  |  |
|  |  | Platelet count | 10E9/L | 12 | 266.8 | 14.0 | 48.5 | 261 | 189 | 346 |  |  |  |  |  |  |  |  |  |
|  | | | | | | | | | | | | | | | | | | |  |

**Table S8.** **Summary of vital signs results.** Vital signs measurements are presented by treatment and protocol time, including change from baseline (baseline: Day 1 pre dosing). BPM: beats per minute, br/min: breaths per minute, C: degrees Celsius, DiastBPsup: diastolic blood pressure (supine), FU: follow-up visit, PRE: pre dosing, SD: standard deviation, SE: standard error, M: minutes after dosing, min: minimum, max: maximum, SCR: screening visit, SystBPsup: systolic blood pressure (supine).

|  | | | | **Result** | | | | | | | **\| Change from baseline** | | | | | | |
| --- | --- | --- | --- | --- | --- | --- | --- | --- | --- | --- | --- | --- | --- | --- | --- | --- | --- |
| **Treatment** | **Protocol time** | **Label** | **Unit** | **N** | **mean** | **SE** | **SD** | **median** | **min** | **max** | **N** | **mean** | **SE** | **SD** | **median** | **min** | **max** |
| EDP1815 EC1 | SCR | Respiration Rate | br/min | 13 | 14.8 | 0.7 | 2.4 | 14 | 12 | 20 |  |  |  |  |  |  |  |
|  |  | SystBPsup | mmHg | 13 | 116.6 | 2.6 | 9.3 | 115 | 104 | 136 |  |  |  |  |  |  |  |
|  |  | DiastBPsup | mmHg | 13 | 67.9 | 1.9 | 6.9 | 68 | 51 | 81 |  |  |  |  |  |  |  |
|  |  | Pulse Rate supine | bpm | 13 | 57.2 | 1.7 | 6.2 | 56 | 51 | 69 |  |  |  |  |  |  |  |
|  |  | Body temperature | C | 13 | 36.75 | 0.07 | 0.25 | 36.7 | 36.3 | 37.1 |  |  |  |  |  |  |  |
|  | Day -1 | Respiration Rate | br/min | 13 | 14.0 | 0.9 | 3.4 | 14 | 10 | 20 |  |  |  |  |  |  |  |
|  |  | SystBPsup | mmHg | 13 | 112.9 | 3.4 | 12.2 | 109 | 100 | 138 |  |  |  |  |  |  |  |
|  |  | DiastBPsup | mmHg | 13 | 64.5 | 2.0 | 7.1 | 62 | 56 | 78 |  |  |  |  |  |  |  |
|  |  | Pulse Rate supine | bpm | 13 | 60.2 | 1.8 | 6.6 | 60 | 50 | 71 |  |  |  |  |  |  |  |
|  |  | Body temperature | C | 13 | 36.52 | 0.15 | 0.55 | 36.5 | 35.5 | 37.4 |  |  |  |  |  |  |  |
|  | Day 1 PRE | Respiration Rate | br/min | 13 | 14.6 | 1.0 | 3.7 | 14 | 12 | 24 |  |  |  |  |  |  |  |
|  |  | SystBPsup | mmHg | 13 | 111.9 | 2.5 | 8.9 | 112 | 92 | 122 |  |  |  |  |  |  |  |
|  |  | DiastBPsup | mmHg | 13 | 64.9 | 1.8 | 6.7 | 63 | 56 | 77 |  |  |  |  |  |  |  |
|  |  | Pulse Rate supine | bpm | 13 | 64.1 | 2.5 | 8.8 | 62 | 54 | 77 |  |  |  |  |  |  |  |
|  |  | Body temperature | C | 13 | 36.41 | 0.12 | 0.43 | 36.5 | 35.6 | 37.0 |  |  |  |  |  |  |  |
|  | Day 8 PRE | Respiration Rate | br/min | 3 | 12.0 | 1.2 | 2.0 | 12 | 10 | 14 | 3 | -5.3 | 3.3 | 5.8 | -2 | -12 | -2 |
|  |  | SystBPsup | mmHg | 3 | 115.0 | 5.3 | 9.2 | 117 | 105 | 123 | 3 | 0.7 | 2.0 | 3.5 | 1 | -3 | 4 |
|  |  | DiastBPsup | mmHg | 3 | 68.0 | 1.7 | 3.0 | 68 | 65 | 71 | 3 | 4.3 | 6.8 | 11.7 | 9 | -9 | 13 |
|  |  | Pulse Rate supine | bpm | 3 | 57.3 | 8.1 | 14.0 | 53 | 46 | 73 | 3 | -3.3 | 2.6 | 4.5 | -3 | -8 | 1 |
|  |  | Body temperature | C | 13 | 36.45 | 0.15 | 0.54 | 36.3 | 35.4 | 37.2 | 13 | 0.04 | 0.17 | 0.63 | 0.0 | -0.8 | 1.6 |
|  | Day 8 30M | Respiration Rate | br/min | 13 | 14.3 | 0.7 | 2.4 | 14 | 10 | 18 | 13 | -0.3 | 1.1 | 3.8 | 0 | -10 | 4 |
|  |  | SystBPsup | mmHg | 13 | 112.4 | 2.0 | 7.1 | 109 | 103 | 126 | 13 | 0.5 | 1.9 | 7.0 | 2 | -12 | 15 |
|  |  | DiastBPsup | mmHg | 13 | 65.3 | 2.0 | 7.0 | 64 | 55 | 77 | 13 | 0.4 | 2.3 | 8.2 | 0 | -12 | 18 |
|  |  | Pulse Rate supine | bpm | 13 | 58.1 | 2.7 | 9.8 | 57 | 44 | 79 | 13 | -6.0 | 2.6 | 9.3 | -8 | -20 | 9 |
|  | Day 22 PRE | Respiration Rate | br/min | 12 | 13.2 | 0.4 | 1.3 | 13 | 12 | 16 | 12 | -1.7 | 1.1 | 3.8 | 0 | -12 | 2 |
|  |  | SystBPsup | mmHg | 12 | 110.8 | 3.0 | 10.3 | 111 | 94 | 130 | 12 | -0.4 | 1.9 | 6.4 | -1 | -12 | 8 |
|  |  | DiastBPsup | mmHg | 12 | 64.0 | 2.1 | 7.1 | 65 | 50 | 76 | 12 | -0.1 | 2.0 | 6.9 | -1 | -12 | 13 |
|  |  | Pulse Rate supine | bpm | 12 | 60.4 | 2.2 | 7.6 | 63 | 43 | 69 | 12 | -2.8 | 2.0 | 7.0 | -4 | -13 | 8 |
|  |  | Body temperature | C | 12 | 36.48 | 0.15 | 0.53 | 36.5 | 35.6 | 37.2 | 12 | 0.08 | 0.17 | 0.58 | 0.0 | -0.8 | 1.3 |
|  | Day 22 30M | Respiration Rate | br/min | 12 | 13.6 | 0.6 | 2.2 | 14 | 9 | 16 | 12 | -1.3 | 1.4 | 4.7 | -1 | -12 | 4 |
|  |  | SystBPsup | mmHg | 12 | 113.1 | 4.0 | 13.9 | 110 | 94 | 144 | 12 | 1.8 | 2.7 | 9.5 | 2 | -14 | 22 |
|  |  | DiastBPsup | mmHg | 12 | 62.0 | 2.0 | 7.0 | 63 | 51 | 72 | 12 | -2.1 | 2.2 | 7.6 | -2 | -17 | 10 |
|  |  | Pulse Rate supine | bpm | 12 | 56.6 | 2.3 | 8.1 | 57 | 43 | 68 | 12 | -6.7 | 2.6 | 9.1 | -9 | -20 | 10 |
|  | | | | | | | | | | | | | | | | | |
| EDP1815 EC1 | Day 36 PRE | Respiration Rate | br/min | 12 | 13.8 | 0.7 | 2.5 | 14 | 10 | 18 | 12 | -1.0 | 1.3 | 4.6 | 0 | -12 | 6 |
|  |  | SystBPsup | mmHg | 12 | 115.8 | 3.4 | 11.8 | 116 | 92 | 138 | 12 | 4.6 | 1.4 | 5.0 | 3 | -1 | 16 |
|  |  | DiastBPsup | mmHg | 12 | 67.0 | 1.7 | 5.8 | 68 | 56 | 75 | 12 | 2.9 | 1.5 | 5.3 | 2 | -7 | 14 |
|  |  | Pulse Rate supine | bpm | 12 | 62.8 | 2.2 | 7.6 | 60 | 53 | 75 | 12 | -0.4 | 2.2 | 7.5 | 0 | -18 | 12 |
|  |  | Body temperature | C | 12 | 36.67 | 0.11 | 0.37 | 36.8 | 36.0 | 37.2 | 12 | 0.27 | 0.14 | 0.49 | 0.2 | -0.6 | 1.3 |
|  | Day 36 30M | Respiration Rate | br/min | 12 | 14.5 | 0.4 | 1.5 | 14 | 12 | 16 | 12 | -0.3 | 1.0 | 3.4 | 1 | -8 | 4 |
|  |  | SystBPsup | mmHg | 12 | 113.4 | 3.4 | 11.6 | 111 | 101 | 134 | 12 | 2.2 | 2.6 | 9.0 | 2 | -18 | 18 |
|  |  | DiastBPsup | mmHg | 12 | 67.8 | 2.2 | 7.8 | 69 | 50 | 83 | 12 | 3.8 | 1.5 | 5.2 | 5 | -6 | 12 |
|  |  | Pulse Rate supine | bpm | 12 | 56.5 | 1.8 | 6.3 | 56 | 47 | 66 | 12 | -6.8 | 2.5 | 8.7 | -5 | -21 | 7 |
|  | Day 57 PRE | Respiration Rate | br/min | 12 | 15.2 | 0.7 | 2.5 | 16 | 12 | 20 | 12 | 0.3 | 1.5 | 5.0 | 0 | -10 | 8 |
|  |  | SystBPsup | mmHg | 12 | 115.3 | 3.4 | 11.9 | 110 | 102 | 136 | 12 | 4.1 | 2.5 | 8.6 | 3 | -15 | 14 |
|  |  | DiastBPsup | mmHg | 12 | 67.4 | 2.3 | 8.0 | 67 | 57 | 88 | 12 | 3.3 | 2.5 | 8.5 | 2 | -10 | 20 |
|  |  | Pulse Rate supine | bpm | 12 | 60.8 | 2.3 | 7.9 | 59 | 50 | 80 | 12 | -2.5 | 2.5 | 8.7 | -3 | -17 | 9 |
|  |  | Body temperature | C | 12 | 36.48 | 0.08 | 0.29 | 36.5 | 36.0 | 36.9 | 12 | 0.08 | 0.13 | 0.46 | -0.1 | -0.5 | 1.1 |
|  | Day 57 1H | Respiration Rate | br/min | 12 | 16.0 | 0.8 | 2.7 | 16 | 12 | 20 | 12 | 1.2 | 1.5 | 5.1 | 3 | -12 | 6 |
|  |  | SystBPsup | mmHg | 12 | 114.3 | 4.3 | 14.8 | 114 | 90 | 138 | 12 | 3.1 | 3.1 | 10.9 | 4 | -17 | 22 |
|  |  | DiastBPsup | mmHg | 12 | 67.0 | 2.6 | 9.1 | 66 | 57 | 86 | 12 | 2.9 | 2.2 | 7.8 | 3 | -13 | 18 |
|  |  | Pulse Rate supine | bpm | 12 | 61.2 | 2.2 | 7.7 | 63 | 46 | 72 | 12 | -2.1 | 1.8 | 6.3 | -3 | -10 | 10 |
|  | Day 58 | Body temperature | C | 12 | 36.65 | 0.08 | 0.27 | 36.6 | 36.3 | 37.2 | 12 | 0.25 | 0.11 | 0.38 | 0.3 | -0.2 | 1.1 |
|  | Day 59 | Body temperature | C | 12 | 36.75 | 0.09 | 0.32 | 36.7 | 36.3 | 37.3 | 12 | 0.35 | 0.14 | 0.47 | 0.2 | -0.5 | 1.5 |
|  | Day 60 | Respiration Rate | br/min | 12 | 14.4 | 1.2 | 4.2 | 14 | 8 | 24 | 12 | -0.4 | 1.6 | 5.5 | 0 | -16 | 7 |
|  |  | SystBPsup | mmHg | 12 | 114.4 | 3.5 | 12.0 | 113 | 99 | 140 | 12 | 3.2 | 2.5 | 8.7 | 5 | -11 | 18 |
|  |  | DiastBPsup | mmHg | 12 | 65.4 | 2.3 | 8.0 | 66 | 51 | 80 | 12 | 1.3 | 2.1 | 7.4 | 1 | -12 | 12 |
|  |  | Pulse Rate supine | bpm | 12 | 61.0 | 2.1 | 7.4 | 59 | 48 | 77 | 12 | -2.3 | 1.8 | 6.2 | -1 | -14 | 5 |
|  |  | Body temperature | C | 12 | 36.46 | 0.11 | 0.38 | 36.6 | 35.9 | 37.2 | 12 | 0.06 | 0.12 | 0.43 | -0.1 | -0.4 | 1.1 |
|  | FU | Respiration Rate | br/min | 13 | 15.4 | 0.9 | 3.2 | 16 | 12 | 20 |  |  |  |  |  |  |  |
|  |  | SystBPsup | mmHg | 13 | 115.6 | 3.4 | 12.4 | 116 | 94 | 138 |  |  |  |  |  |  |  |
|  |  | DiastBPsup | mmHg | 13 | 68.9 | 1.4 | 5.1 | 68 | 62 | 78 |  |  |  |  |  |  |  |
|  |  | Pulse Rate supine | bpm | 13 | 59.9 | 2.1 | 7.7 | 63 | 42 | 69 |  |  |  |  |  |  |  |
|  |  | Body temperature | C | 13 | 36.52 | 0.10 | 0.37 | 36.4 | 36.0 | 37.4 |  |  |  |  |  |  |  |
| EDP1815 EC2 | SCR | Respiration Rate | br/min | 13 | 13.4 | 0.5 | 1.7 | 14 | 10 | 16 |  |  |  |  |  |  |  |
|  |  | SystBPsup | mmHg | 13 | 112.4 | 2.6 | 9.3 | 109 | 103 | 127 |  |  |  |  |  |  |  |
|  |  | DiastBPsup | mmHg | 13 | 67.9 | 1.9 | 6.7 | 68 | 56 | 77 |  |  |  |  |  |  |  |
|  | | | | | | | | | | | | | | | | | |

|  | | | | | | | | | | | | | | | | | | |
| --- | --- | --- | --- | --- | --- | --- | --- | --- | --- | --- | --- | --- | --- | --- | --- | --- | --- | --- |
|  | | | | **Result** | | | | | | | **\| Change from baseline** | | | | | | |  |
| **Treatment** | **Protocol time** | **Label** | **Unit** | **N** | **mean** | **SE** | **SD** | **median** | **min** | **max** | **N** | **mean** | **SE** | **SD** | **median** | **min** | **max** |  |
| EDP1815 EC2 | SCR | Pulse Rate supine | bpm | 13 | 57.3 | 2.1 | 7.7 | 55 | 45 | 67 |  |  |  |  |  |  |  |  |
|  |  | Body temperature | C | 13 | 36.58 | 0.07 | 0.27 | 36.6 | 36.1 | 36.9 |  |  |  |  |  |  |  |  |
|  | Day -1 | Respiration Rate | br/min | 13 | 14.3 | 0.6 | 2.3 | 14 | 12 | 18 |  |  |  |  |  |  |  |  |
|  |  | SystBPsup | mmHg | 13 | 108.9 | 2.7 | 9.6 | 108 | 95 | 135 |  |  |  |  |  |  |  |  |
|  |  | DiastBPsup | mmHg | 13 | 63.5 | 1.9 | 6.9 | 62 | 52 | 75 |  |  |  |  |  |  |  |  |
|  |  | Pulse Rate supine | bpm | 13 | 59.5 | 2.2 | 7.9 | 59 | 46 | 74 |  |  |  |  |  |  |  |  |
|  |  | Body temperature | C | 13 | 36.45 | 0.09 | 0.34 | 36.5 | 35.6 | 36.9 |  |  |  |  |  |  |  |  |
|  | Day 1 PRE | Respiration Rate | br/min | 13 | 15.8 | 1.3 | 4.9 | 14 | 12 | 28 |  |  |  |  |  |  |  |  |
|  |  | SystBPsup | mmHg | 13 | 108.8 | 2.1 | 7.5 | 106 | 100 | 122 |  |  |  |  |  |  |  |  |
|  |  | DiastBPsup | mmHg | 13 | 66.8 | 1.7 | 6.1 | 69 | 57 | 77 |  |  |  |  |  |  |  |  |
|  |  | Pulse Rate supine | bpm | 13 | 57.9 | 2.7 | 9.7 | 60 | 43 | 75 |  |  |  |  |  |  |  |  |
|  |  | Body temperature | C | 13 | 36.40 | 0.12 | 0.45 | 36.5 | 35.7 | 37.1 |  |  |  |  |  |  |  |  |
|  | Day 8 PRE | Respiration Rate | br/min | 13 | 14.3 | 0.5 | 2.0 | 14 | 12 | 18 | 13 | -1.5 | 1.5 | 5.2 | -2 | -12 | 6 |  |
|  |  | SystBPsup | mmHg | 13 | 111.8 | 3.3 | 11.9 | 110 | 94 | 137 | 13 | 3.0 | 2.4 | 8.6 | 3 | -9 | 16 |  |
|  |  | DiastBPsup | mmHg | 13 | 69.2 | 2.2 | 7.8 | 70 | 54 | 83 | 13 | 2.3 | 1.9 | 6.8 | 2 | -7 | 14 |  |
|  |  | Pulse Rate supine | bpm | 13 | 60.2 | 3.0 | 11.0 | 59 | 45 | 85 | 13 | 2.3 | 1.4 | 5.2 | 3 | -5 | 10 |  |
|  |  | Body temperature | C | 13 | 36.45 | 0.10 | 0.35 | 36.5 | 36.0 | 37.2 | 13 | 0.05 | 0.13 | 0.45 | 0.1 | -0.6 | 1.1 |  |
|  | Day 8 30M | Respiration Rate | br/min | 12 | 13.5 | 0.7 | 2.6 | 14 | 10 | 16 | 12 | -2.7 | 1.5 | 5.1 | -2 | -12 | 4 |  |
|  |  | SystBPsup | mmHg | 12 | 111.0 | 3.0 | 10.3 | 111 | 94 | 131 | 12 | 1.5 | 2.8 | 9.8 | 4 | -12 | 15 |  |
|  |  | DiastBPsup | mmHg | 12 | 67.9 | 2.7 | 9.5 | 70 | 49 | 79 | 12 | 1.3 | 1.9 | 6.5 | 3 | -9 | 12 |  |
|  |  | Pulse Rate supine | bpm | 12 | 52.3 | 2.5 | 8.6 | 51 | 40 | 71 | 12 | -5.1 | 1.7 | 5.7 | -6 | -13 | 6 |  |
|  | Day 22 PRE | Respiration Rate | br/min | 12 | 15.0 | 0.8 | 2.6 | 14 | 12 | 20 | 12 | -1.2 | 1.2 | 4.0 | 0 | -10 | 4 |  |
|  |  | SystBPsup | mmHg | 12 | 107.4 | 2.6 | 8.8 | 108 | 96 | 129 | 12 | -2.1 | 2.4 | 8.2 | -3 | -14 | 13 |  |
|  |  | DiastBPsup | mmHg | 12 | 63.9 | 2.2 | 7.5 | 62 | 51 | 75 | 12 | -2.8 | 2.1 | 7.4 | -2 | -15 | 9 |  |
|  |  | Pulse Rate supine | bpm | 12 | 57.1 | 2.7 | 9.4 | 57 | 45 | 74 | 12 | -0.3 | 1.7 | 5.8 | -2 | -10 | 8 |  |
|  |  | Body temperature | C | 12 | 36.44 | 0.13 | 0.45 | 36.5 | 35.9 | 37.2 | 12 | -0.01 | 0.11 | 0.37 | -0.1 | -0.5 | 0.7 |  |
|  | Day 22 30M | Respiration Rate | br/min | 12 | 15.0 | 1.2 | 4.2 | 14 | 10 | 26 | 12 | -1.2 | 0.8 | 2.8 | 0 | -8 | 2 |  |
|  |  | SystBPsup | mmHg | 12 | 109.5 | 2.3 | 7.9 | 110 | 96 | 120 | 12 | 0.0 | 2.3 | 7.9 | 4 | -15 | 9 |  |
|  |  | DiastBPsup | mmHg | 12 | 64.5 | 2.8 | 9.6 | 61 | 55 | 79 | 12 | -2.2 | 2.2 | 7.6 | -3 | -14 | 12 |  |
|  |  | Pulse Rate supine | bpm | 12 | 51.8 | 3.4 | 11.7 | 48 | 39 | 80 | 12 | -5.6 | 2.0 | 7.1 | -6 | -19 | 5 |  |
|  | Day 36 PRE | Respiration Rate | br/min | 12 | 14.3 | 1.0 | 3.5 | 14 | 10 | 24 | 12 | -1.8 | 1.1 | 3.8 | -2 | -8 | 2 |  |
|  |  | SystBPsup | mmHg | 12 | 110.7 | 2.4 | 8.4 | 109 | 100 | 125 | 12 | 1.2 | 1.1 | 3.7 | 1 | -4 | 9 |  |
|  |  | DiastBPsup | mmHg | 12 | 65.0 | 2.5 | 8.6 | 65 | 53 | 85 | 12 | -1.7 | 1.7 | 6.0 | -1 | -16 | 8 |  |
|  | | | | | | | | | | | | | | | | | |  |

|  | | | | | | | | | | | | | | | | | | |
| --- | --- | --- | --- | --- | --- | --- | --- | --- | --- | --- | --- | --- | --- | --- | --- | --- | --- | --- |
|  | | | | **Result** | | | | | | | **\| Change from baseline** | | | | | | |  |
| **Treatment** | **Protocol time** | **Label** | **Unit** | **N** | **mean** | **SE** | **SD** | **median** | **min** | **max** | **N** | **mean** | **SE** | **SD** | **median** | **min** | **max** |  |
| EDP1815 EC2 | Day 36 PRE | Pulse Rate supine | bpm | 12 | 59.7 | 3.7 | 12.7 | 56 | 45 | 83 | 12 | 2.3 | 2.2 | 7.8 | 3 | -8 | 15 |  |
|  |  | Body temperature | C | 12 | 36.39 | 0.10 | 0.35 | 36.4 | 35.9 | 37.2 | 12 | -0.06 | 0.11 | 0.38 | -0.1 | -0.7 | 0.6 |  |
|  | Day 36 30M | Respiration Rate | br/min | 12 | 14.0 | 0.7 | 2.4 | 14 | 10 | 18 | 12 | -2.2 | 1.5 | 5.1 | -3 | -10 | 4 |  |
|  |  | SystBPsup | mmHg | 12 | 109.2 | 3.4 | 11.9 | 106 | 97 | 138 | 12 | -0.3 | 2.4 | 8.2 | -2 | -12 | 16 |  |
|  |  | DiastBPsup | mmHg | 12 | 65.5 | 2.3 | 7.9 | 65 | 57 | 84 | 12 | -1.2 | 1.7 | 6.0 | -3 | -12 | 12 |  |
|  |  | Pulse Rate supine | bpm | 12 | 53.1 | 1.7 | 5.8 | 54 | 44 | 60 | 12 | -4.3 | 2.0 | 6.8 | -6 | -15 | 9 |  |
|  | Day 57 PRE | Respiration Rate | br/min | 12 | 14.9 | 0.6 | 2.1 | 16 | 10 | 17 | 12 | -1.3 | 1.6 | 5.4 | 1 | -12 | 4 |  |
|  |  | SystBPsup | mmHg | 12 | 111.6 | 2.4 | 8.4 | 110 | 102 | 129 | 12 | 2.1 | 1.5 | 5.3 | 3 | -7 | 13 |  |
|  |  | DiastBPsup | mmHg | 12 | 65.3 | 1.9 | 6.6 | 64 | 56 | 76 | 12 | -1.3 | 2.2 | 7.6 | -1 | -13 | 16 |  |
|  |  | Pulse Rate supine | bpm | 12 | 58.3 | 3.4 | 11.7 | 57 | 37 | 78 | 12 | 1.0 | 1.9 | 6.7 | 3 | -12 | 11 |  |
|  |  | Body temperature | C | 12 | 36.16 | 0.11 | 0.38 | 36.2 | 35.6 | 36.6 | 12 | -0.29 | 0.09 | 0.33 | -0.3 | -0.8 | 0.3 |  |
|  | Day 57 1H | Respiration Rate | br/min | 12 | 14.1 | 0.5 | 1.6 | 14 | 12 | 16 | 12 | -2.1 | 1.2 | 4.2 | 0 | -12 | 2 |  |
|  |  | SystBPsup | mmHg | 12 | 111.8 | 3.2 | 10.9 | 111 | 96 | 133 | 12 | 2.3 | 2.2 | 7.4 | 2 | -11 | 17 |  |
|  |  | DiastBPsup | mmHg | 12 | 64.8 | 2.1 | 7.4 | 65 | 53 | 76 | 12 | -1.8 | 2.2 | 7.7 | -4 | -10 | 17 |  |
|  |  | Pulse Rate supine | bpm | 12 | 53.2 | 2.7 | 9.2 | 52 | 40 | 73 | 12 | -4.2 | 2.3 | 7.9 | -5 | -16 | 12 |  |
|  | Day 58 | Body temperature | C | 12 | 36.10 | 0.12 | 0.42 | 36.1 | 35.5 | 36.8 | 12 | -0.35 | 0.07 | 0.24 | -0.4 | -0.7 | 0.2 |  |
|  | Day 59 | Body temperature | C | 12 | 36.21 | 0.16 | 0.55 | 36.3 | 35.5 | 37.3 | 12 | -0.24 | 0.12 | 0.40 | -0.3 | -0.7 | 0.7 |  |
|  | Day 60 | Respiration Rate | br/min | 12 | 13.2 | 0.3 | 1.0 | 14 | 12 | 14 | 12 | -3.0 | 1.6 | 5.5 | -2 | -16 | 2 |  |
|  |  | SystBPsup | mmHg | 12 | 110.3 | 2.3 | 8.1 | 109 | 103 | 131 | 12 | 0.8 | 1.5 | 5.3 | 0 | -8 | 9 |  |
|  |  | DiastBPsup | mmHg | 12 | 65.0 | 2.2 | 7.6 | 65 | 52 | 78 | 12 | -1.7 | 1.8 | 6.2 | -2 | -12 | 10 |  |
|  |  | Pulse Rate supine | bpm | 12 | 56.1 | 2.7 | 9.3 | 56 | 41 | 72 | 12 | -1.3 | 1.8 | 6.1 | 1 | -13 | 7 |  |
|  |  | Body temperature | C | 12 | 36.26 | 0.11 | 0.38 | 36.5 | 35.6 | 36.7 | 12 | -0.19 | 0.18 | 0.61 | -0.2 | -1.3 | 0.8 |  |
|  | FU | Respiration Rate | br/min | 13 | 14.8 | 0.8 | 2.8 | 15 | 8 | 20 |  |  |  |  |  |  |  |  |
|  |  | SystBPsup | mmHg | 13 | 113.5 | 3.0 | 10.9 | 113 | 97 | 133 |  |  |  |  |  |  |  |  |
|  |  | DiastBPsup | mmHg | 13 | 67.0 | 2.9 | 10.3 | 64 | 56 | 86 |  |  |  |  |  |  |  |  |
|  |  | Pulse Rate supine | bpm | 13 | 58.5 | 2.8 | 10.1 | 59 | 45 | 78 |  |  |  |  |  |  |  |  |
|  |  | Body temperature | C | 13 | 36.55 | 0.13 | 0.47 | 36.5 | 35.8 | 37.5 |  |  |  |  |  |  |  |  |
| Placebo | SCR | Respiration Rate | br/min | 12 | 14.2 | 0.4 | 1.5 | 14 | 12 | 16 |  |  |  |  |  |  |  |  |
|  |  | SystBPsup | mmHg | 12 | 125.4 | 3.2 | 11.1 | 127 | 106 | 142 |  |  |  |  |  |  |  |  |
|  |  | DiastBPsup | mmHg | 12 | 71.4 | 2.1 | 7.4 | 72 | 58 | 82 |  |  |  |  |  |  |  |  |
|  |  | Pulse Rate supine | bpm | 12 | 59.7 | 2.5 | 8.7 | 58 | 48 | 77 |  |  |  |  |  |  |  |  |
|  |  | Body temperature | C | 12 | 36.80 | 0.11 | 0.40 | 36.8 | 36.0 | 37.6 |  |  |  |  |  |  |  |  |
|  | Day -1 | Respiration Rate | br/min | 12 | 13.7 | 0.8 | 2.8 | 14 | 8 | 18 |  |  |  |  |  |  |  |  |
|  | | | | | | | | | | | | | | | | | |  |

|  | | | | | | | | | | | | | | | | | | |
| --- | --- | --- | --- | --- | --- | --- | --- | --- | --- | --- | --- | --- | --- | --- | --- | --- | --- | --- |
|  | | | | **Result** | | | | | | | **\| Change from baseline** | | | | | | |  |
| **Treatment** | **Protocol time** | **Label** | **Unit** | **N** | **mean** | **SE** | **SD** | **median** | **min** | **max** | **N** | **mean** | **SE** | **SD** | **median** | **min** | **max** |  |
| Placebo | Day -1 | SystBPsup | mmHg | 12 | 116.3 | 2.7 | 9.3 | 117 | 100 | 139 |  |  |  |  |  |  |  |  |
|  |  | DiastBPsup | mmHg | 12 | 67.8 | 1.0 | 3.6 | 69 | 62 | 73 |  |  |  |  |  |  |  |  |
|  |  | Pulse Rate supine | bpm | 12 | 56.8 | 2.0 | 7.0 | 55 | 47 | 72 |  |  |  |  |  |  |  |  |
|  |  | Body temperature | C | 12 | 36.41 | 0.13 | 0.44 | 36.4 | 35.8 | 37.3 |  |  |  |  |  |  |  |  |
|  | Day 1 PRE | Respiration Rate | br/min | 12 | 13.3 | 1.0 | 3.4 | 13 | 6 | 18 |  |  |  |  |  |  |  |  |
|  |  | SystBPsup | mmHg | 12 | 119.3 | 3.1 | 10.8 | 119 | 99 | 144 |  |  |  |  |  |  |  |  |
|  |  | DiastBPsup | mmHg | 12 | 64.7 | 1.5 | 5.4 | 64 | 56 | 74 |  |  |  |  |  |  |  |  |
|  |  | Pulse Rate supine | bpm | 12 | 61.8 | 2.6 | 8.9 | 62 | 48 | 79 |  |  |  |  |  |  |  |  |
|  |  | Body temperature | C | 12 | 36.57 | 0.17 | 0.60 | 36.7 | 35.5 | 37.2 |  |  |  |  |  |  |  |  |
|  | Day 8 PRE | Respiration Rate | br/min | 8 | 14.0 | 0.7 | 1.9 | 14 | 12 | 16 | 8 | 1.5 | 1.3 | 3.8 | 1 | -4 | 8 |  |
|  |  | SystBPsup | mmHg | 8 | 120.1 | 6.0 | 16.9 | 115 | 101 | 146 | 8 | 1.4 | 3.3 | 9.3 | 2 | -12 | 17 |  |
|  |  | DiastBPsup | mmHg | 8 | 66.8 | 1.3 | 3.6 | 66 | 63 | 74 | 8 | 2.6 | 2.0 | 5.7 | 4 | -9 | 11 |  |
|  |  | Pulse Rate supine | bpm | 8 | 58.9 | 2.3 | 6.4 | 59 | 48 | 69 | 8 | -1.0 | 2.9 | 8.1 | 0 | -16 | 8 |  |
|  |  | Body temperature | C | 12 | 36.24 | 0.18 | 0.63 | 36.3 | 35.3 | 37.6 | 12 | -0.33 | 0.11 | 0.38 | -0.5 | -0.8 | 0.4 |  |
|  | Day 8 30M | Respiration Rate | br/min | 12 | 13.7 | 0.6 | 2.2 | 14 | 10 | 18 | 12 | 0.3 | 1.2 | 4.0 | 0 | -4 | 8 |  |
|  |  | SystBPsup | mmHg | 12 | 115.3 | 4.4 | 15.3 | 116 | 93 | 144 | 12 | -4.0 | 2.2 | 7.7 | -3 | -21 | 12 |  |
|  |  | DiastBPsup | mmHg | 12 | 66.3 | 1.9 | 6.5 | 65 | 57 | 80 | 12 | 1.7 | 2.1 | 7.2 | 1 | -11 | 12 |  |
|  |  | Pulse Rate supine | bpm | 12 | 54.7 | 2.7 | 9.3 | 55 | 42 | 72 | 12 | -7.2 | 2.4 | 8.3 | -6 | -25 | 4 |  |
|  | Day 22 PRE | Respiration Rate | br/min | 12 | 13.8 | 0.5 | 1.8 | 14 | 12 | 16 | 12 | 0.5 | 1.3 | 4.5 | 0 | -6 | 10 |  |
|  |  | SystBPsup | mmHg | 12 | 114.1 | 2.2 | 7.5 | 114 | 99 | 128 | 12 | -5.3 | 2.1 | 7.2 | -5 | -16 | 5 |  |
|  |  | DiastBPsup | mmHg | 12 | 63.1 | 1.8 | 6.4 | 61 | 55 | 75 | 12 | -1.6 | 2.1 | 7.1 | -2 | -13 | 10 |  |
|  |  | Pulse Rate supine | bpm | 12 | 60.6 | 2.7 | 9.4 | 58 | 49 | 82 | 12 | -1.3 | 2.5 | 8.7 | 1 | -20 | 11 |  |
|  |  | Body temperature | C | 12 | 36.49 | 0.15 | 0.51 | 36.5 | 35.8 | 37.3 | 12 | -0.08 | 0.17 | 0.60 | 0.1 | -1.1 | 0.7 |  |
|  | Day 22 30M | Respiration Rate | br/min | 12 | 12.2 | 0.6 | 2.2 | 12 | 8 | 16 | 12 | -1.2 | 0.9 | 3.0 | -1 | -6 | 4 |  |
|  |  | SystBPsup | mmHg | 12 | 115.3 | 2.9 | 10.0 | 114 | 95 | 131 | 12 | -4.1 | 2.0 | 7.0 | -4 | -17 | 10 |  |
|  |  | DiastBPsup | mmHg | 12 | 65.0 | 2.1 | 7.3 | 65 | 56 | 79 | 12 | 0.3 | 2.5 | 8.6 | -1 | -11 | 14 |  |
|  |  | Pulse Rate supine | bpm | 12 | 54.2 | 2.5 | 8.6 | 51 | 45 | 75 | 12 | -7.7 | 2.1 | 7.1 | -6 | -24 | 1 |  |
|  | Day 36 PRE | Respiration Rate | br/min | 12 | 13.5 | 0.4 | 1.2 | 14 | 12 | 16 | 12 | 0.2 | 1.0 | 3.6 | 0 | -6 | 6 |  |
|  |  | SystBPsup | mmHg | 12 | 116.6 | 2.2 | 7.5 | 117 | 108 | 132 | 12 | -2.8 | 2.3 | 8.1 | -5 | -12 | 12 |  |
|  |  | DiastBPsup | mmHg | 12 | 66.2 | 2.5 | 8.7 | 67 | 56 | 84 | 12 | 1.5 | 2.7 | 9.3 | 0 | -12 | 18 |  |
|  |  | Pulse Rate supine | bpm | 12 | 59.0 | 2.8 | 9.7 | 56 | 42 | 80 | 12 | -2.8 | 2.2 | 7.6 | -3 | -21 | 8 |  |
|  |  | Body temperature | C | 12 | 36.47 | 0.11 | 0.37 | 36.4 | 35.9 | 37.1 | 12 | -0.10 | 0.13 | 0.44 | -0.2 | -0.6 | 0.9 |  |
|  | Day 36 30M | Respiration Rate | br/min | 12 | 13.0 | 0.5 | 1.6 | 13 | 10 | 16 | 12 | -0.3 | 0.8 | 2.9 | 0 | -6 | 4 |  |
|  | | | | | | | | | | | | | | | | | |  |
|  | | | | **Result** | | | | | | | **\| Change from baseline** | | | | | | |  |
| **Treatment** | **Protocol time** | **Label** | **Unit** | **N** | **mean** | **SE** | **SD** | **median** | **min** | **max** | **N** | **mean** | **SE** | **SD** | **median** | **min** | **max** |  |
| Placebo | Day 36 30M | SystBPsup | mmHg | 12 | 114.0 | 2.4 | 8.4 | 115 | 100 | 134 | 12 | -5.3 | 2.2 | 7.5 | -8 | -18 | 5 |  |
|  |  | DiastBPsup | mmHg | 12 | 64.5 | 2.6 | 9.1 | 63 | 51 | 82 | 12 | -0.2 | 3.1 | 10.8 | -3 | -23 | 21 |  |
|  |  | Pulse Rate supine | bpm | 12 | 52.8 | 2.4 | 8.2 | 53 | 40 | 72 | 12 | -9.1 | 1.7 | 6.1 | -8 | -22 | 1 |  |
|  | Day 57 PRE | Respiration Rate | br/min | 12 | 14.1 | 0.7 | 2.6 | 14 | 11 | 18 | 12 | 0.8 | 1.2 | 4.0 | 1 | -6 | 8 |  |
|  |  | SystBPsup | mmHg | 12 | 118.8 | 2.9 | 10.0 | 117 | 108 | 146 | 12 | -0.5 | 1.6 | 5.6 | -1 | -10 | 9 |  |
|  |  | DiastBPsup | mmHg | 12 | 68.3 | 1.9 | 6.6 | 68 | 58 | 82 | 12 | 3.6 | 2.0 | 6.9 | 4 | -6 | 13 |  |
|  |  | Pulse Rate supine | bpm | 12 | 60.1 | 2.5 | 8.7 | 59 | 47 | 77 | 12 | -1.8 | 2.5 | 8.8 | 0 | -19 | 9 |  |
|  |  | Body temperature | C | 12 | 36.46 | 0.16 | 0.57 | 36.6 | 35.4 | 37.6 | 12 | -0.11 | 0.18 | 0.63 | -0.2 | -1.0 | 1.3 |  |
|  | Day 57 1H | Respiration Rate | br/min | 12 | 14.5 | 0.7 | 2.6 | 15 | 10 | 18 | 12 | 1.2 | 0.9 | 3.1 | 1 | -4 | 6 |  |
|  |  | SystBPsup | mmHg | 12 | 116.0 | 3.1 | 10.7 | 116 | 100 | 138 | 12 | -3.3 | 1.8 | 6.2 | -4 | -13 | 7 |  |
|  |  | DiastBPsup | mmHg | 12 | 68.3 | 2.2 | 7.5 | 69 | 53 | 84 | 12 | 3.6 | 2.0 | 7.0 | 5 | -11 | 13 |  |
|  |  | Pulse Rate supine | bpm | 12 | 55.7 | 3.1 | 10.9 | 54 | 42 | 80 | 12 | -6.2 | 2.7 | 9.5 | -6 | -24 | 12 |  |
|  | Day 58 | Body temperature | C | 12 | 36.31 | 0.16 | 0.54 | 36.4 | 35.3 | 37.0 | 12 | -0.26 | 0.13 | 0.46 | -0.3 | -1.1 | 0.6 |  |
|  | Day 59 | Body temperature | C | 12 | 36.38 | 0.12 | 0.42 | 36.4 | 35.5 | 37.1 | 12 | -0.19 | 0.18 | 0.62 | -0.3 | -1.0 | 1.3 |  |
|  | Day 60 | Respiration Rate | br/min | 12 | 13.3 | 0.6 | 2.0 | 14 | 10 | 18 | 12 | 0.0 | 1.3 | 4.3 | 0 | -6 | 8 |  |
|  |  | SystBPsup | mmHg | 12 | 114.2 | 2.0 | 6.9 | 114 | 100 | 127 | 12 | -5.2 | 1.7 | 5.8 | -4 | -20 | 1 |  |
|  |  | DiastBPsup | mmHg | 12 | 66.1 | 2.3 | 7.9 | 65 | 59 | 87 | 12 | 1.4 | 2.4 | 8.4 | 1 | -8 | 16 |  |
|  |  | Pulse Rate supine | bpm | 12 | 57.8 | 2.5 | 8.6 | 57 | 47 | 77 | 12 | -4.1 | 2.3 | 8.0 | -2 | -22 | 5 |  |
|  |  | Body temperature | C | 12 | 36.34 | 0.14 | 0.48 | 36.4 | 35.7 | 37.1 | 12 | -0.23 | 0.13 | 0.44 | -0.3 | -0.8 | 0.8 |  |
|  | FU | Respiration Rate | br/min | 12 | 13.2 | 0.5 | 1.8 | 13 | 10 | 16 |  |  |  |  |  |  |  |  |
|  |  | SystBPsup | mmHg | 12 | 116.1 | 3.7 | 12.9 | 115 | 95 | 149 |  |  |  |  |  |  |  |  |
|  |  | DiastBPsup | mmHg | 12 | 66.8 | 1.5 | 5.2 | 69 | 56 | 73 |  |  |  |  |  |  |  |  |
|  |  | Pulse Rate supine | bpm | 12 | 57.0 | 3.1 | 10.7 | 56 | 38 | 73 |  |  |  |  |  |  |  |  |
|  |  | Body temperature | C | 12 | 36.76 | 0.12 | 0.41 | 36.7 | 36.2 | 37.5 |  |  |  |  |  |  |  |  |

**Table S9.** **Summary of electrocardiography (ECG) results.** ECG results are presented by treatment and protocol time, including change from baseline (baseline: Day -1). BPM: beats per minute, FU: follow-up visit, HR: heart rate, PRE: pre dosing, SD: standard deviation, SE: standard error, min: minimum, max: maximum, SCR: screening visit.

|  | | | | **Result** | | | | | | | **\| Change from baseline** | | | | | | |
| --- | --- | --- | --- | --- | --- | --- | --- | --- | --- | --- | --- | --- | --- | --- | --- | --- | --- |
| **Treatment** | **Protocol time** | **Label** | **Unit** | **N** | **mean** | **SE** | **SD** | **median** | **min** | **max** | **N** | **mean** | **SE** | **SD** | **median** | **min** | **max** |
| EDP1815 EC1 | SCR | HR | bpm | 13 | 59.5 | 2.0 | 7.2 | 58 | 48 | 71 |  |  |  |  |  |  |  |
|  |  | PR interval | msec | 13 | 150.6 | 6.4 | 23.1 | 150 | 112 | 204 |  |  |  |  |  |  |  |
|  |  | QRS duration | msec | 13 | 90.5 | 3.1 | 11.2 | 88 | 74 | 108 |  |  |  |  |  |  |  |
|  |  | QT interval | msec | 13 | 412.5 | 3.8 | 13.8 | 414 | 376 | 430 |  |  |  |  |  |  |  |
|  |  | QTcF | msec | 13 | 410.2 | 4.2 | 15.1 | 413 | 382 | 427 |  |  |  |  |  |  |  |
|  | Day -1 | HR | bpm | 13 | 61.5 | 1.7 | 6.2 | 61 | 53 | 73 |  |  |  |  |  |  |  |
|  |  | PR interval | msec | 13 | 153.4 | 6.2 | 22.3 | 150 | 116 | 192 |  |  |  |  |  |  |  |
|  |  | QRS duration | msec | 13 | 90.6 | 3.2 | 11.6 | 90 | 72 | 108 |  |  |  |  |  |  |  |
|  |  | QT interval | msec | 13 | 409.7 | 5.7 | 20.7 | 410 | 360 | 446 |  |  |  |  |  |  |  |
|  |  | QTcF | msec | 13 | 411.8 | 4.3 | 15.4 | 411 | 382 | 434 |  |  |  |  |  |  |  |
|  | Day 22 | HR | bpm | 12 | 61.1 | 2.1 | 7.2 | 63 | 47 | 71 | 12 | -0.2 | 2.2 | 7.7 | -2 | -8 | 17 |
|  |  | PR interval | msec | 12 | 156.7 | 4.5 | 15.6 | 154 | 138 | 192 | 12 | 0.2 | 3.7 | 12.7 | 1 | -30 | 18 |
|  |  | QRS duration | msec | 12 | 92.2 | 3.7 | 12.7 | 90 | 72 | 112 | 12 | 0.8 | 0.9 | 3.1 | 2 | -6 | 4 |
|  |  | QT interval | msec | 12 | 405.5 | 7.1 | 24.4 | 402 | 356 | 454 | 12 | -5.3 | 5.1 | 17.6 | -4 | -44 | 30 |
|  |  | QTcF | msec | 12 | 406.3 | 5.2 | 18.1 | 411 | 363 | 428 | 12 | -6.3 | 3.3 | 11.4 | -5 | -24 | 7 |
|  | Day 60 | HR | bpm | 12 | 62.0 | 1.9 | 6.7 | 62 | 50 | 70 | 12 | 0.8 | 2.0 | 7.0 | 0 | -10 | 17 |
|  |  | PR interval | msec | 12 | 153.8 | 4.6 | 15.8 | 150 | 130 | 182 | 12 | -2.7 | 2.3 | 8.0 | -3 | -10 | 18 |
|  |  | QRS duration | msec | 12 | 90.7 | 3.2 | 11.0 | 92 | 72 | 108 | 12 | -0.7 | 0.8 | 2.9 | 0 | -4 | 6 |
|  |  | QT interval | msec | 12 | 404.2 | 6.1 | 21.1 | 406 | 358 | 436 | 12 | -6.7 | 4.9 | 16.9 | -2 | -40 | 18 |
|  |  | QTcF | msec | 12 | 407.3 | 4.5 | 15.6 | 411 | 377 | 428 | 12 | -5.3 | 2.7 | 9.5 | -6 | -27 | 7 |
|  | FU | HR | bpm | 13 | 58.5 | 2.0 | 7.1 | 60 | 47 | 69 |  |  |  |  |  |  |  |
|  |  | PR interval | msec | 13 | 147.2 | 5.5 | 19.7 | 144 | 110 | 194 |  |  |  |  |  |  |  |
|  |  | QRS duration | msec | 13 | 90.5 | 2.8 | 10.0 | 90 | 76 | 108 |  |  |  |  |  |  |  |
|  |  | QT interval | msec | 13 | 414.0 | 4.2 | 15.1 | 414 | 392 | 444 |  |  |  |  |  |  |  |
|  |  | QTcF | msec | 13 | 409.2 | 5.0 | 17.9 | 407 | 369 | 444 |  |  |  |  |  |  |  |
| EDP1815 EC2 | SCR | HR | bpm | 13 | 58.6 | 2.1 | 7.4 | 57 | 48 | 72 |  |  |  |  |  |  |  |
|  |  | PR interval | msec | 13 | 149.8 | 7.3 | 26.4 | 150 | 114 | 200 |  |  |  |  |  |  |  |
|  |  | QRS duration | msec | 13 | 95.1 | 2.2 | 7.8 | 96 | 78 | 106 |  |  |  |  |  |  |  |
|  |  | QT interval | msec | 13 | 418.9 | 6.5 | 23.4 | 416 | 384 | 462 |  |  |  |  |  |  |  |
|  |  | QTcF | msec | 13 | 414.1 | 5.0 | 18.0 | 409 | 388 | 443 |  |  |  |  |  |  |  |
|  | Day -1 | HR | bpm | 13 | 60.2 | 2.6 | 9.4 | 60 | 44 | 80 |  |  |  |  |  |  |  |
|  |  | PR interval | msec | 13 | 159.8 | 7.9 | 28.3 | 160 | 114 | 208 |  |  |  |  |  |  |  |
|  |  | QRS duration | msec | 13 | 94.6 | 2.1 | 7.7 | 96 | 80 | 108 |  |  |  |  |  |  |  |
|  | | | | | | | | | | | | | | | | | |
| EDP1815 EC2 | Day -1 | QT interval | msec | 13 | 418.9 | 6.9 | 25.0 | 420 | 366 | 466 |  |  |  |  |  |  |  |
|  |  | QTcF | msec | 13 | 417.3 | 5.2 | 18.8 | 420 | 378 | 448 |  |  |  |  |  |  |  |
|  | Day 22 | HR | bpm | 12 | 56.6 | 3.1 | 10.9 | 56 | 41 | 78 | 12 | -3.3 | 2.2 | 7.7 | -4 | -18 | 7 |
|  |  | PR interval | msec | 12 | 156.7 | 6.7 | 23.3 | 158 | 120 | 206 | 12 | -3.8 | 3.3 | 11.5 | 0 | -26 | 12 |
|  |  | QRS duration | msec | 12 | 94.5 | 2.5 | 8.5 | 95 | 76 | 104 | 12 | 0.0 | 1.5 | 5.2 | -2 | -4 | 10 |
|  |  | QT interval | msec | 12 | 421.0 | 8.4 | 29.0 | 421 | 366 | 462 | 12 | 0.8 | 6.4 | 22.2 | 5 | -32 | 26 |
|  |  | QTcF | msec | 12 | 409.5 | 4.4 | 15.3 | 407 | 383 | 446 | 12 | -8.1 | 3.1 | 10.7 | -6 | -25 | 5 |
|  | Day 60 | HR | bpm | 12 | 56.6 | 2.3 | 8.1 | 55 | 46 | 70 | 12 | -3.3 | 2.5 | 8.8 | -4 | -19 | 10 |
|  |  | PR interval | msec | 12 | 153.8 | 7.2 | 25.1 | 157 | 110 | 208 | 12 | -6.7 | 3.5 | 12.0 | -3 | -32 | 10 |
|  |  | QRS duration | msec | 12 | 93.5 | 2.7 | 9.5 | 94 | 76 | 106 | 12 | -1.0 | 1.4 | 4.8 | 0 | -10 | 6 |
|  |  | QT interval | msec | 12 | 418.2 | 7.2 | 25.1 | 416 | 372 | 464 | 12 | -2.0 | 5.8 | 20.3 | -1 | -34 | 30 |
|  |  | QTcF | msec | 12 | 408.2 | 6.0 | 20.8 | 409 | 373 | 445 | 12 | -9.4 | 4.1 | 14.2 | -4 | -40 | 4 |
|  | FU | HR | bpm | 13 | 57.5 | 2.9 | 10.6 | 57 | 43 | 76 |  |  |  |  |  |  |  |
|  |  | PR interval | msec | 13 | 154.3 | 8.4 | 30.2 | 150 | 118 | 216 |  |  |  |  |  |  |  |
|  |  | QRS duration | msec | 13 | 95.2 | 2.0 | 7.3 | 96 | 82 | 106 |  |  |  |  |  |  |  |
|  |  | QT interval | msec | 13 | 418.5 | 6.8 | 24.6 | 414 | 380 | 464 |  |  |  |  |  |  |  |
|  |  | QTcF | msec | 13 | 409.8 | 5.6 | 20.2 | 409 | 370 | 447 |  |  |  |  |  |  |  |
| Placebo | SCR | HR | bpm | 12 | 59.6 | 2.8 | 9.5 | 58 | 48 | 76 |  |  |  |  |  |  |  |
|  |  | PR interval | msec | 12 | 148.2 | 5.7 | 19.9 | 149 | 112 | 174 |  |  |  |  |  |  |  |
|  |  | QRS duration | msec | 12 | 95.5 | 2.1 | 7.2 | 97 | 82 | 104 |  |  |  |  |  |  |  |
|  |  | QT interval | msec | 12 | 425.3 | 12.0 | 41.6 | 426 | 356 | 510 |  |  |  |  |  |  |  |
|  |  | QTcF | msec | 12 | 421.2 | 7.1 | 24.7 | 414 | 385 | 473 |  |  |  |  |  |  |  |
|  | Day -1 | HR | bpm | 12 | 57.3 | 2.3 | 8.0 | 56 | 45 | 74 |  |  |  |  |  |  |  |
|  |  | PR interval | msec | 12 | 151.2 | 5.7 | 19.6 | 156 | 116 | 178 |  |  |  |  |  |  |  |
|  |  | QRS duration | msec | 12 | 95.2 | 3.1 | 10.8 | 93 | 82 | 114 |  |  |  |  |  |  |  |
|  |  | QT interval | msec | 12 | 424.3 | 11.1 | 38.3 | 422 | 356 | 496 |  |  |  |  |  |  |  |
|  |  | QTcF | msec | 12 | 415.5 | 6.6 | 23.0 | 412 | 371 | 458 |  |  |  |  |  |  |  |
|  | Day 22 | HR | bpm | 12 | 59.3 | 2.6 | 9.0 | 58 | 47 | 80 | 12 | 2.0 | 1.4 | 4.8 | 2 | -10 | 8 |
|  |  | PR interval | msec | 12 | 152.5 | 4.8 | 16.7 | 154 | 126 | 176 | 12 | 1.3 | 3.4 | 11.9 | -1 | -14 | 26 |
|  |  | QRS duration | msec | 12 | 95.8 | 2.3 | 8.1 | 96 | 84 | 108 | 12 | 0.7 | 1.6 | 5.6 | 0 | -8 | 10 |
|  |  | QT interval | msec | 12 | 419.0 | 12.1 | 41.8 | 419 | 352 | 508 | 12 | -5.3 | 5.2 | 18.2 | -9 | -24 | 42 |
|  |  | QTcF | msec | 12 | 414.3 | 6.8 | 23.5 | 413 | 381 | 468 | 12 | -1.2 | 3.1 | 10.9 | -3 | -17 | 18 |
|  | Day 60 | HR | bpm | 12 | 59.1 | 2.4 | 8.3 | 58 | 47 | 76 | 12 | 1.8 | 1.5 | 5.2 | 2 | -6 | 11 |
|  | | | | | | | | | | | | | | | | | |

|  | | | | | | | | | | | | | | | | | | |
| --- | --- | --- | --- | --- | --- | --- | --- | --- | --- | --- | --- | --- | --- | --- | --- | --- | --- | --- |
|  | | | | **Result** | | | | | | | **\| Change from baseline** | | | | | | |  |
| **Treatment** | **Protocol time** | **Label** | **Unit** | **N** | **mean** | **SE** | **SD** | **median** | **min** | **max** | **N** | **mean** | **SE** | **SD** | **median** | **min** | **max** |  |
| Placebo | Day 60 | PR interval | msec | 12 | 154.8 | 4.8 | 16.5 | 153 | 132 | 184 | 12 | 3.7 | 3.8 | 13.2 | 4 | -26 | 20 |  |
|  |  | QRS duration | msec | 12 | 90.5 | 2.1 | 7.3 | 89 | 80 | 106 | 12 | -4.7 | 2.1 | 7.3 | -2 | -26 | 4 |  |
|  |  | QT interval | msec | 12 | 414.7 | 10.2 | 35.4 | 411 | 360 | 478 | 12 | -9.7 | 3.3 | 11.4 | -10 | -24 | 12 |  |
|  |  | QTcF | msec | 12 | 410.0 | 5.6 | 19.3 | 408 | 387 | 449 | 12 | -5.5 | 3.0 | 10.5 | -8 | -23 | 18 |  |
|  | FU | HR | bpm | 12 | 59.8 | 3.1 | 10.6 | 61 | 44 | 78 |  |  |  |  |  |  |  |  |
|  |  | PR interval | msec | 12 | 149.5 | 4.6 | 16.0 | 150 | 128 | 176 |  |  |  |  |  |  |  |  |
|  |  | QRS duration | msec | 12 | 93.0 | 2.2 | 7.7 | 93 | 80 | 108 |  |  |  |  |  |  |  |  |
|  |  | QT interval | msec | 12 | 422.0 | 13.6 | 47.3 | 409 | 364 | 518 |  |  |  |  |  |  |  |  |
|  |  | QTcF | msec | 12 | 417.6 | 7.2 | 25.1 | 410 | 390 | 467 |  |  |  |  |  |  |  |  |

**Table S10.** Least square mean (LSM) differences, 95% confidence intervals (CIs) and p-values of differences between EDP1815-treated participants and placebo participants, for all pharmacodynamic outcomes of the Keyhole Limpet Haemocyanin (KLH) challenge.

|  | **EDP1815-EC1 - Placebo** | | | **EDP1815-EC2 - Placebo** | | |
| --- | --- | --- | --- | --- | --- | --- |
|  | **LSM difference** | **95% CI** | **p-value** | **LSM difference** | **95% CI** | **p-value** |
| **LSCI – basal flow (AU)** | -0.992 | -7.841 – 5.858 | 0.7700 | 0.898 | -6.005 – 7.801 | 0.7928 |
| **LSCI – flare (AU)** | -62.517 | -280.898 – 155.864 | 0.5639 | -22.779 | -240.191 –194.633 | 0.8324 |
| **Multispectral imaging – erythema (AU)** | 0.2401 | -0.4761 – 0.9562 | 0.4996 | 0.0680 | -0.6577 – 0.7937 | 0.8498 |
| **Anti-KLH IgG (%)** | 164.61 | -36.63 – 365.85 | 0.1056 | 53.27 | -146.48 – 253.02 | 0.5909 |
| **Anti-KLH IgM (%)** | 7.30 | -109.90 –124.51 | 0.8999 | -45.46 | -162.95 – 72.03 | 0.4369 |

AU: arbitrary units; CI: confidence interval; LSCI: laser speckle contrast imaging; LSM: least square mean

**Table S11.** Least square mean (LSM) differences, 95% confidence intervals (CIs) and p-values of differences between EDP1815-treated participants and placebo participants, for all imaging outcomes of the imiquimod challenge.

|  | **EDP1815-EC1 - Placebo** | | | **EDP1815-EC2 - Placebo** | | |
| --- | --- | --- | --- | --- | --- | --- |
|  | **LSM difference** | **95% CI** | **p-value** | **LSM difference** | **95% CI** | **p-value** |
| **LSCI – basal flow (AU)** | -6.040 | -57.660 – 45.580 | 0.8131 | -12.605 | -62.770 – 37.560 | 0.6123 |
| **LSCI – flare (AU)** | 4.234 | -25.718 – 34.187 | 0.7752 | 8.832 | -21.544 – 39.208 | 0.5579 |
| **Multispectral imaging – erythema (AU)** | -0.224 | -2.5531 – 2.1046 | 0.8457 | -1.042 | -3.3691 – 1.2856 | 0.3688 |

AU: arbitrary units; CI: confidence interval; LSCI: laser speckle contrast imaging; LSM: least square mean

**Table S12.** Least square mean (LSM) differences, 95% confidence intervals (CIs) and p-values of differences between EDP1815-treated participants and placebo participants, for all cell subsets measured in blister fluid during the imiquimod challenge. LOG absolute numbers of cells were used and back transformed. LSMs and p-values could not be calculated for intermediate monocytes, non classical monocytes and plasmacytoid dendritic cells due to a non-normal distribution of measurements.

|  | **EDP1815-EC1 - Placebo** | | | **EDP1815-EC2 - Placebo** | | |
| --- | --- | --- | --- | --- | --- | --- |
|  | **LSM difference** | **95% CI** | **p-value** | **LSM difference** | **95% CI** | **p-value** |
| **Number of total cells** | -38.7% | -62.0 – 1.1% | 0.0460* | -18.1% | -48.8 – 31.2% | 0.4352 |
| **Number of B cells** | -40.9% | -67.7 – 7.9% | 0.0854 | -11.1% | -51.9 – 64.3% | 0.7431 |
| **Number of T cells** | -38.9% | -62.7 – 0.2% | 0.0523 | -8.9% | -44.0 – 48.0% | 0.7030 |
| **Number of Natural Killer cells** | -46.6% | -71.1 – 1.4% | 0.0460* | -33.5% | -63.8 – 21.9% | 0.1899 |
| **Number of neutrophils** | -58.6% | -78.3 – -20.0% | 0.0107* | -10.8% | -53.4 – 70.9% | 0.8016 |
| **Number of cytotoxic T cells** | -38.3% | -70.3 – 28.0 | 0.1858 | -38.7% | -70.1 – 25.8% | 0.1990 |
| **Number of T helper cells** | -38.4% | -63.7 – 4.5% | 0.07110 | 0.3% | -40.5 – 69.0% | 0.9958 |
| **Number of classical monocytes** | -20.4% | -57.3 – 48.4% | 0.4689 | -8.0% | -50.4 – 70.9% | 0.7317 |
| **Number of granulocytes** | -48.4% | -69.4 – 13.1% | 0.0143* | -9.0% | -45.7 – 52.3% | 0.8085 |
| **Number of myeloid dendritic cells** | -40.7% | -69.4 – 14.9% | 0.1181 | 13.9% | -40.8 – 119.2% | 0.6047 |

CI: confidence interval; LSM: least square mean

**Table S13.** Least square mean (LSM) differences, 95% confidence intervals (CIs) and p-values of differences between EDP1815-treated participants and placebo participants, for cytokine levels in blister fluid during the imiquimod challenge. These values could only be calculated for interleukin (IL)-8 and CXC-motif chemokine ligand 10 (CXCL-10) due to the high number of measurements below limit of quantification of the other cytokines. For IL-8 LOG concentrations were used and back transformed.

|  | **EDP1815-EC1 - Placebo** | | | **EDP1815-EC2 - Placebo** | | |
| --- | --- | --- | --- | --- | --- | --- |
|  | **LSM difference** | **95% CI** | **p-value** | **LSM difference** | **95% CI** | **p-value** |
| **IL-8** | -50.1% | -79.4 – 20.7% | 0.1181 | -54.2% | -80.9 – 9.6% | 0.0775 |
| **CXCL-10 (pg/mL)** | 13908.9 | -261841 – 234023.1 | 0.9094 | 74460.29 | -175773 – 324693.7 | 0.5481 |

CI: confidence interval; CXCL: CXC-motif chemokine ligand; IL: interleukin

Assessed for eligibility (n=67)

Excluded (n=29)

♦  Not meeting inclusion criteria (n=23)

♦  Declined to participate (n=2)

♦  Other reasons (n=4)

Analysed EDP1815-EC1 (n=12) or EDP1815-EC2 (n=12)
♦ Excluded from analysis (n=0)

Discontinued intervention (n=1)*

Lost to follow-up (n=1)**

Allocated to intervention EDP1815-EC1 (n=13) or EDP1815-EC2 (n=13)

♦ Received allocated intervention (n=26)

♦ Did not receive allocated intervention (n=0)

Lost to follow-up (n=0)

Discontinued intervention (n=0)

Allocated to placebo (n=12)

♦ Received allocated intervention (n=12)

♦ Did not receive allocated intervention (n=0)

## Allocation

## Analysis

## Follow-Up

Randomized (n=38)

## Enrollment

Analysed (n=12)
♦ Excluded from analysis (n=0)

**Figure S2.** CONSORT Flow Diagram. The study was conducted in two Cohorts. In both cohorts, 12 participants received active treatment (Cohort 1: EDP1815-EC1, Cohort 2: EDP1815-EC2) and 6 participants received placebo. *In Cohort 1 (EDP1815-EC1), one participant discontinued intervention because of an adverse event (erythema annulare). **In Cohort 2 (EDP1815-EC2), one participant withdrew from the study. In both Cohorts the participant that discontinued the study was replaced by another participant, therefore in total 13 participants on EDP1815 treatment per Cohort were included.
